# Membrane-anchored DNA nanodevice with allosteric aptamer arms enables parallel probing and on-site drug delivery

**DOI:** 10.1038/s41467-026-75300-5

**Published:** 2026-07-07

**Authors:** Meiqin Zhang, Yinghao Cheng, Nan Chen, Wenbin Huang, Linwen Lan, Yi Yuan, Jiangchuan Du, Linjun Zhang, Xuefen Chen, Zhifa Shen, Chang Xue

**Affiliations:** 1 Key Laboratory of Laboratory Medicine, Ministry of Education, Zhejiang Provincial Key Laboratory of Medical Genetics, School of Laboratory Medicine and Life Sciences, Wenzhou Medical University, Wenzhou, Zhejiang 325035 China; 2 Cixi Biomedical Research Institute, Wenzhou Medical University, Wenzhou, Zhejiang 325035 China

**Keywords:** Bioinspired materials, DNA and RNA, Nanobiotechnology

## Abstract

DNA-based molecular computation enables targeting of cells via multiple surface receptors. However, existing platforms are often limited to single-receptor detection per operation or rely on freely diffusing components, a common source of off-target interference. Here we show an intelligent DNA nanodevice (DND) that integrates multivalent recognition, logic‑gated computation, and spatially precise drug delivery in a single nanostructure. The DND comprises a doxorubicin-loaded tetrahedral DNA framework (tFNA@Dox) connected via three allosteric aptamer arms to a cholesterol-modified membrane anchor. Simultaneous binding of all three aptamers triggers an AND‑logic gate, releasing tFNA@Dox specifically at the target cell membrane. This localized sense‑and‑act mechanism maximizes therapeutic specificity and minimizes systemic exposure. In tumor-bearing mice, DND@Dox effectively accumulated in tumors, significantly inhibited tumor growth under local and systemic administration, and reduced systemic toxicity. This work establishes a versatile platform for logic‑controlled, multimarker‑guided cancer theranostics.

## Abstract (web summary)

DNA device-based targeting has huge potential but can be limited by single target recognition resulting in off target effects. Here, the authors develop a DNA nanodevice that anchors to cell membranes and releases chemotherapy only when three specific cancer markers are detected simultaneously.

## Introduction

Cancer remains a leading cause of global mortality, with ~35 million new cases and 19 million related deaths estimated annually by 2050^1,2^. The clinical management of cancer continues to be limited by the insufficient selectivity of conventional chemotherapeutics, which often fail to achieve effective intratumoral drug accumulation while inducing severe off-target toxicity in healthy tissues^3,4^. Dose escalation exacerbates these systemic side effects and can accelerate the emergence of drug resistance, further limiting therapeutic efficacy^5^. To address these limitations, targeted delivery strategies such as antibody‒drug conjugates (ADCs) and antibody-functionalized nanocarriers have been developed to enhance tumor-specific drug delivery^6,7^. Despite significant advances, these antibody-based platforms are limited by complex manufacturing processes, batch-to-batch heterogeneity, and high production costs, hindering their widespread clinical translation^8,9^. More fundamentally, their reliance on single-antigen recognition renders them intrinsically vulnerable to tumor heterogeneity and prevents adequate discrimination between malignant cells and normal tissues that express the same antigen, often leading to dose-limiting toxicity^6,10^. This challenge is exemplified by the clinical use of anti-GD2 immunotherapy for high-risk neuroblastoma: Although GD2-targeting antibodies such as dinutuximab demonstrate efficacy, their binding to GD2 expressed on peripheral sensory nerves induces severe neuropathic pain, a major treatment-limiting side effect^11,12^. Collectively, these shortcomings underscore an urgent need for a new generation of therapeutic platforms capable of performing logical operations to specifically trigger drug delivery only upon the concurrent recognition of multiple disease‑specific biomarkers while also remaining structurally defined, synthetically tractable, and operationally robust under physiological conditions.

DNA nanotechnology offers a uniquely programmable foundation for constructing such intelligent systems. The predictable nature of Watson–Crick base-pairing enables the precise, sequence-directed self-assembly of well-defined two- and three-dimensional nanostructures with spatially addressable functionalization sites^13,14^. These DNA architectures are inherently biocompatible, biodegradable, and readily modified with a variety of functional moieties, making them exceptionally suitable for biomedical applications^15,16^. When combined with biomimetic design principles that extract structural and functional inspiration from natural organisms, DNA nanotechnology offers a powerful pathway for creating sophisticated bio‑inspired nanodevices^17,18^. For instance, researchers have engineered DNA-based assemblies that emulate membrane channels to regulate molecular transport, as well as dynamic nanomechanical systems capable of enhanced pathogen capture for diagnostic applications^19,20^. Despite these advances, most current biomimetic DNA constructs remain relatively simplistic, replicating isolated structural features or singular functions rather than integrating the coordinated perception-decision-action cycle characteristics of living systems. To bridge this gap and enable true multi-input biological sensing, DNA scaffolds must be coupled with programmable molecular recognition elements capable of interpreting complex cellular signatures with high specificity and logical control.

DNA aptamers, which are single-stranded oligonucleotides selected in vitro to bind molecular targets with high affinity and specificity, represent an ideal recognition component for such integrated systems^21^. Early monovalent aptamer designs, exemplified by assays targeting prostate-specific antigen, often suffer from limited diagnostic specificity because of the elevated expression of such biomarkers in benign conditions^22^. Subsequent multivalent architectures that incorporate multiple copies of the same aptamer can increase binding avidity, as exemplified by the viral-capturing DNA nanoGripper^19^. However, such valency-based strategies remain fundamentally unable to overcome the challenge of target coexpression in healthy tissues. For example, HER2-targeted therapies, while effective in treating breast cancer, carry a well-documented risk of cardiotoxicity because of the physiological expression of HER2 in cardiomyocytes^23^. Thus, simply increasing aptamer valency does not confer the discriminant power required for safe targeting in heterogeneous biological environments. To achieve higher specificity, recent efforts have focused on integrating multiple distinct aptamers within a single nanostructure to enable the concurrent detection of several biomarkers. Notable examples include a tri-aptamer-guided DNA missile that releases doxorubicin upon decoding cancer cell surface signatures^24^ and a multi-aptamer proximity ligation platform for one-step tumor cell isolation and subtyping^25^. While these systems represent important progress, they predominantly rely on the diffusive interaction of multiple free DNA components that undergo sequential toehold-mediated strand displacements at or near the cell membrane. In complex biological fluids, such multi-component architectures are prone to kinetic delays, signal leakage, and nonspecific activation, limiting their robustness and reliability in vivo. Although an integrated aptamer-gated DNA nanophage was recently reported to confine logic operations within a single particle^26,27^, its signaling mechanism still depends on a cascade of free DNA strand-displacement reactions, making it susceptible to interference in serum-rich environments. Therefore, a fully integrated nanoplatform that confines both sensing and actuation within a single, structurally coordinated entity is needed to eliminate the dependence on diffusible intermediates and perform robust logic-gated operations directly on target cell surfaces.

Here, we report a DNA nanodevice (DND) designed to meet this need. The DND integrates parallel multi-target sensing, Boolean computation, and spatially precise drug delivery within a single membrane-anchored nanostructure. By co-localizing sensing, computation, and actuation within one rigid nanoscale framework, the DND operates at high effective local concentrations with minimal signal transduction distance and an architecturally enforced reaction sequence. This integrated design suppresses off-target activation and signal crosstalk while preserving the benefits of multivalent binding, thereby achieving high specificity without compromising affinity or kinetic efficiency. By implementing the perception-decision-action paradigm of a living organism in a synthetic nanomachine, this work establishes a versatile and reprogrammable platform for logic-controlled cancer theranostics, offering a generalizable blueprint for next-generation bio-inspired intelligent drug delivery systems.

## Results

### Working principle of a DNA nanodevice for parallel probing and logic‑gated drug delivery

**Fig. 1:**
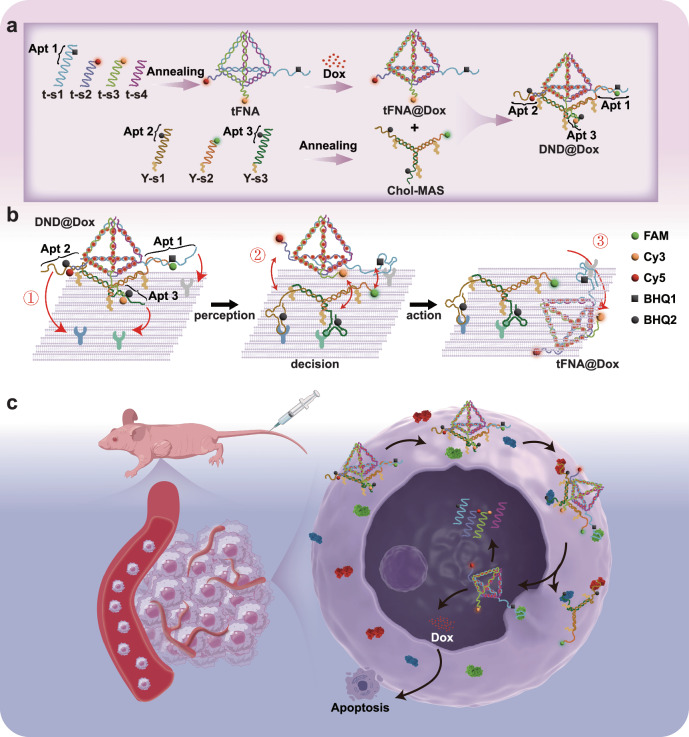
Design and working mechanism of the DNA Nanodevice (DND). **a** Modular assembly of the DND from a doxorubicin-loaded DNA tetrahedron (tFNA@Dox) and a cholesterol‑modified Y‑shaped scaffold (Chol-MAS) bearing aptamer locks. **b** Parallel, multivalent recognition of cell surface receptors triggers AND-gated disassembly and simultaneous fluorescence activation. **c** In vivo logic-guided targeting leads to tumor-specific internalization and programmed drug delivery, inducing apoptosis.

By integrating sensing, decision-making, and actuation capabilities, we designed a DND that performs cell-surface molecular computation to enable spatially controlled chemotherapy. As illustrated in Fig. 1a, the DND is modularly assembled from two DNA components: a doxorubicin-loaded tetrahedral framework nucleic acid (tFNA@Dox) serving as the head, and a cholesterol-modified Y-shaped membrane-anchoring scaffold (Chol-MAS) that functions as the base. One aptamer (Apt 1) is integrated into the tFNA@Dox, while two additional aptamers (Apt 2 and Apt 3) are connected to the Chol-MAS. Through sequence-defined partial complementarity, each aptamer strand hybridizes with a short complementary lock strand extending from the opposing module, thereby forming three bridging duplex arms that connect the head to the base. This design ensures that the molecular recognition elements are pre-positioned across both modules yet are functionally integrated via programmable DNA hybridization. The complete, drug-loaded construct is denoted DND@Dox. Detailed schematics and the full nucleotide sequences are provided in Supplementary Fig. 1 and Supplementary Table 1, respectively. In its pre-activated state, each aptamer is locked in an inactive conformation by its complementary strand. Upon binding to its cognate cell-surface protein, the aptamer undergoes a conformation-specific switch that displaces the lock strand and destabilizes the corresponding DNA bridge. The system was designed as a three-input AND gate with respect to its functional output (payload release), which is generated exclusively when all three inputs are present simultaneously, whereas each fluorescence channel independently reports the binding status of its corresponding aptamer arm. Critically, the three aptamers operate independently, enabling parallel, real-time interrogation of multiple membrane biomarkers. The three allosteric aptamer arms were introduced not simply to enhance binding, but to convert receptor recognition into a combinatorial decision process, so that release of the tetrahedral module occurs only when the predefined multi-marker condition is satisfied. Specifically, allosteric refers to the conformational switching behavior of the aptamer arms, wherein target binding to the aptamer recognition domain induces a structural rearrangement that displaces the complementary lock strand, thereby destabilizing the bridging duplex. Exploratory computational modeling provides supportive evidence for the proposed activation mechanism (Supplementary Figs. 2–4). Only when all three targets are simultaneously engaged, where the system meets a three-input AND logic condition, do the bridging duplexes cooperatively dissociate. This triggers the release of tFNA@Dox from the membrane-anchored base, allowing for the liberated tetrahedron, via its resident aptamer (Apt 1), to mediate efficient cellular internalization and subsequent intracellular drug delivery.

To visually monitor the activation dynamics, we engineered three spectrally distinct fluorescence resonance energy transfer (FRET) channels into DND@Dox (Fig. 1b). Fluorophore–quencher pairs (FAM/BHQ1, Cy3/BHQ2, and Cy5/BHQ2) were conjugated to the three duplex arms. In the locked state, the fluorescence is quenched. Target-induced strand-displacement separates each fluorophore–quencher pair, restoring fluorescence in the corresponding channel. Thus, each arm independently reports its recognition event as an independent recognition signal. Only when all three target proteins are co-expressed on the same cell do the three channels simultaneously switch to the on state, activating the AND gate and triggering drug carrier release. This multi-channel readout provides real-time, spatially resolved verification of logic-gated activation, ensuring highly specific drug delivery while minimizing off-target effects.

In tumor-bearing mice, intravenously administered DND@Dox accumulates in tumor tissue through enhanced permeability and retention effects (Fig. 1c). The cholesterol moieties serve two critical functions: first, they prevent premature endocytosis of the planar structure, as reported in prior studies, thereby avoiding nonspecific intracellular toxicity; second, they prolong the residence time of the nanostructure on the cell membrane, providing an essential temporal window for parallel aptamer recognition and subsequent logic‑gated computation. At the membrane interface, the aptamer arms perform parallel computation of the local receptor profile. Only in cells expressing all three targets are the FRET channels fully activated, leading to the release and internalization of tFNA@Dox. Once inside the cell, the nuclease-rich, mildly acidic environment accelerates degradation of the DNA tetrahedron, resulting in release of intercalated doxorubicin. The drug subsequently accumulates in the nucleus, induces DNA damage, and initiates the apoptosis of target tumor cells. Together, DND@Dox functions as a membrane-anchored logic-gated nanodevice that performs parallel molecular sensing and Boolean computation directly on the cell surface. Its sense-and-act mechanism ensures that therapeutic delivery occurs only when stringent multi-marker logic conditions are met, thereby achieving precise, intelligent, and low-toxicity cancer theranostics.

### Assembly and interfacial behavior of the DNA nanodevice on living cell membranes

**Fig. 2:**
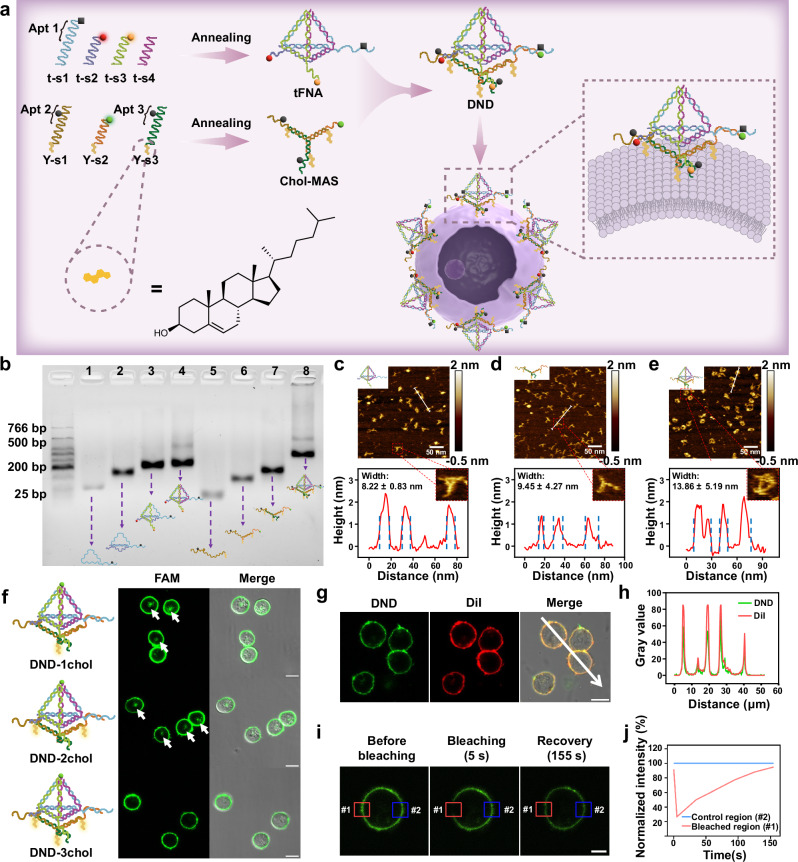
Design and characterization of the membrane-anchored DNA Nanodevice (DND). **a** Self-assembly of the DND from a DNA tetrahedron head (tFNA) and a cholesterol-modified Y-shaped scaffold (Chol-MAS) that mediates cell membrane anchoring. **b** Agarose gel electrophoresis verifying the stepwise assembly of tFNA (Lanes 1–4), Chol-MAS (Lanes 5–7) and the assembled DND (Lane 8). The experiment was repeated independently three times with similar results. **c**–**e** Atomic force microscopy (AFM) images and height profiles of tFNA (**c**), Chol-MAS (**d**), and DND (**e**). Scale bars, 50 nm. **f** Confocal laser scanning microscopy (CLSM) images of Ramos cells incubated with FAM-labeled DND variants (DND-1chol, DND-2chol, and DND-3chol) at 37 °C for 20 min. The white arrows indicate intracellular puncta. Scale bars, 10 μm. **g**, **h** Colocalization of FAM-labeled DND with DiI-stained plasma membranes, as shown by the merged CLSM image (**g**) and the line scan fluorescence profile (**h**). Scale bars, 10 μm. The experiment was repeated independently three times with similar results. **i** Representative CLSM images acquired fluorescence before bleaching, immediately after bleaching (5 s), and during recovery (155 s). Region #1 indicates the bleached area, whereas Region #2 serves as the control. Scale bars, 5 μm. The experiment was repeated independently three times with similar results. **j** Normalized fluorescence recovery curves for the bleached region (#1) and control region (#2). Source data are provided as a Source Data file.

Building on the working principle outlined above, we first assembled and characterized the membrane‑anchored DND structure. As illustrated in Fig. 2a, the DND is constructed by hybridizing a preassembled tetrahedral framework nucleic acid (tFNA) with a cholesterol‑modified Y‑shaped scaffold (Chol‑MAS) via sequence‑complementary sticky ends, a design in which the cholesterol moieties enable the nanostructure to uniformly anchor across the cell membrane surface. Electrophoretic analysis confirmed the stepwise assembly of the nanostructure. Lanes 1–4 and 5–7 show the progressive retardation of tFNA and Chol‑MAS intermediates, respectively. The hybridization of these two modules in Lane 8 produced a single, dominant band with the slowest mobility, confirming the formation of intact DND (Fig. 2b).

Following the confirmation of successful assembly by electrophoresis, we next employed theoretical modeling and high-resolution microscopy to determine the physical dimensions of the nanostructure. Theoretical modeling based on DNA contour length predicted dimensions of ~7.1–31.3 nm for tFNA, 10.2–41.8 nm for Chol‑MAS, and an overall diameter of 10.2–43.9 nm for the assembled DND (Supplementary Fig. 5). These ranges account for the spatial anisotropy inherent to three‑dimensional nucleic acid structures, which present variable widths when measured along different axes, thereby providing a realistic estimate of their physical dimensions. To experimentally validate these predicted dimensions and directly visualize the nanostructures, we performed atomic force microscopy (AFM) and cryo-electron microscopy (Cryo-EM). AFM height analysis, performed under ambient conditions, revealed values of ~2 nm for tFNA (Fig. 2c), ~1.3 nm for Chol‑MAS (Fig. 2d), and ~2 nm for the assembled DND (Fig. 2e). Liquid-mode AFM directly visualized the hydrated nanostructures and yielded lateral widths of 8.22 ± 0.83 nm for tFNA, 9.45 ± 4.27 nm for Chol-MAS, and 13.86 ± 5.19 nm for DND (Fig. 2c–e). In addition, representative Cryo-EM micrographs provided complementary morphological evidence for tFNA, Chol-MAS, and DND in the frozen-hydrated state (Supplementary Fig. 6). Collectively, the complementary data from modeling, liquid-mode AFM, and Cryo-EM support the successful assembly of the DND nanostructure and its expected overall morphology.

Having established the successful assembly and nanoscale dimensions of the DND, we next characterized its interfacial behavior on living cell membranes, beginning with the optimization of its cholesterol-mediated anchoring. Cholesterol-modified DNA inserts into lipid bilayers via hydrophobic and van der Waals interactions, providing a nonspecific anchoring mechanism^28–31^. We functionalized the DND with cholesterol groups to exploit this principle. A critical finding was that DND constructs bearing one or two cholesterol moieties (DND-1chol, DND-2chol) were prone to non-specific endocytosis (Fig. 2f). Confocal imaging in serum‑containing medium revealed remarkable fluorescence decay for DND constructs with zero, one, or two cholesterol anchors (DND‑0/1/2chol) (Supplementary Fig. 7). This signal loss likely results from a combination of nonspecific endocytosis, dissociation from the membrane during processing, and enzymatic degradation. In contrast, the triple‑cholesterol variant (DND‑3chol) formed a planar arrangement that was stably anchored to the cell surface, effectively preventing internalization while maintaining robust membrane association (Fig. 2f), even in the presence of 10% serum (Supplementary Fig. 7). This optimized DND-3chol construct was used in all subsequent studies. Under standardized conditions, confocal imaging with the membrane dye DiI confirmed extensive colocalization of DND-3chol with the plasma membrane across multiple cell lines, and line-scan profiles revealed precise overlap at the cell periphery, confirming exclusive surface localization (Fig. 2g, h, and Supplementary Fig. 8). Treatment with extracellular DNase I abolished fluorescence, verifying an outward orientation with the nucleic acid framework exposed to the extracellular milieu (Supplementary Fig. 9). Importantly, fluorescence recovery after photobleaching (FRAP) experiments demonstrated that membrane-anchored DND-3chol retained significant lateral mobility (Fig. 2i, j). This mobility enables the nanostructure to diffuse across the membrane surface, providing a spatial scanning mechanism that allows for its three-aptamer arms to efficiently survey and engage multiple target receptors on the same cell. This dynamic, surface-confined state is essential for executing the parallel probing and integrated logic-gated recognition that defines the DND’s function.

### In vitro validation of logic‑gated activation in the DNA nanodevice

**Fig. 3:**
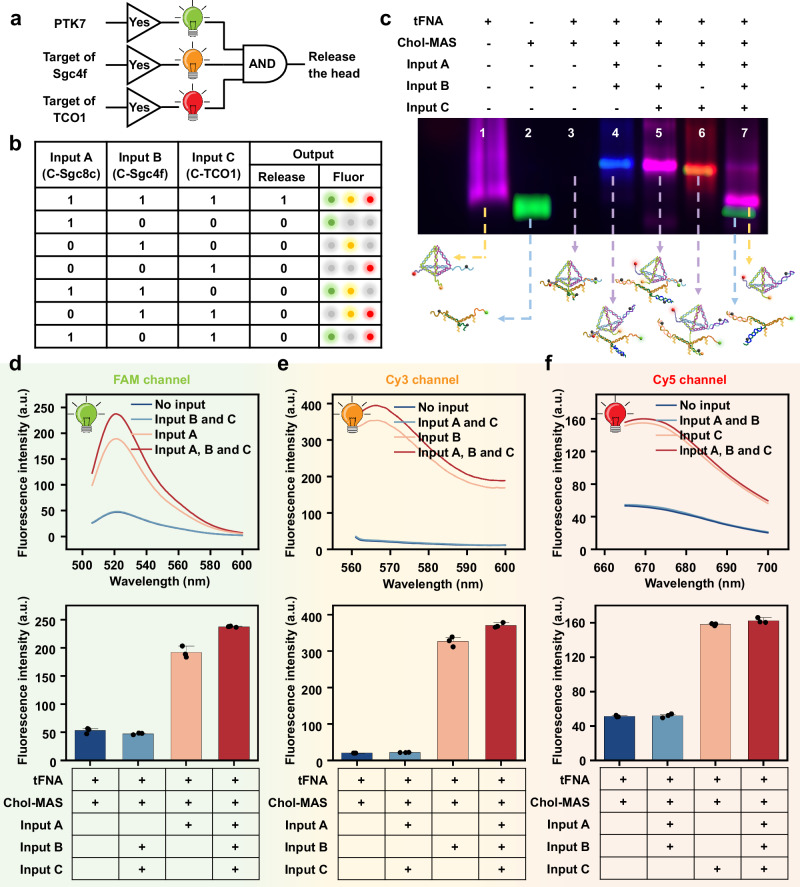
In vitro validation of logic‑gated activation in the DNA Nanodevice (DND). **a** Schematic of a three-input AND logic operation based on aptamer recognition that cooperatively triggers release of the tFNA. PTK7 (FAM), target of Sgc4f (Cy3), and target of TCO1 (Cy5). **b** Truth table for the three‑input AND gate. The functional output (Output = 1, defined as tFNA release) occurs only when all three inputs (A, B, C) are simultaneously present (1,1,1). The fluorescence channels (FAM, Cy3, Cy5) independently report the binding status of each aptamer arm and do not constitute the logic gate output themselves. **c** Agarose gel electrophoresis showing input-dependent disassembly of the DND and release of structural modules. Lane 1, tFNA. Lane 2, Chol-MAS. Lane 3, intact DND. Lanes 4–7, DND incubated with the indicated complementary inputs (C-Sgc8c, C-Sgc4f and/or C-TCO1), yielding target-responsive disassembly patterns. The experiment was repeated independently three times with similar results. **d**–**f** Fluorescence spectra and quantitative analyses showing selective signal outputs in the FAM (**d**), Cy3 (**e**), and Cy5 (**f**) channels under different input combinations. Data are presented as mean ± SD (*n* = 3 from three independent reaction samples). Source data are provided as a Source Data file.

Following the structural and interfacial characterization, we next sought to rigorously validate the core intelligence function of the DND: its ability to execute logic-gated operations and release its payload only upon receiving the correct multi‑input signature. Using CCRF-CEM cells as a model, we configured the three DND arms with the aptamers Sgc8c, Sgc4f, and TCO1, which target PTK7 and two other cell-surface markers (although their molecular identities remain uncharacterized) (Fig. 3a). To precisely mimic target binding and decouple the activation from complex cellular environments, we employed fully complementary DNA strands (C‑Sgc8c, C‑Sgc4f, and C‑TCO1) as synthetic inputs to trigger allosteric switches in vitro^32,33^. The system was designed as a three‑input AND gate, where the logic output 1 (payload release) is generated exclusively when all three inputs are present simultaneously (Fig. 3b). Agarose gel electrophoresis confirmed this gated disassembly: while single or pairwise inputs failed to dissociate the structure, the presence of all three inputs successfully cleaved the DND into its constituent tetrahedral head (tFNA) and Y‑shaped scaffold (Chol‑MAS) (Supplementary Fig. 10). Fluorescence imaging afforded a direct visual readout of this logic (Fig. 3c). Despite the slight smearing, which likely arose from labeling-related electrophoretic broadening rather than intrinsic instability (as indicated by the clear migration of unlabeled tFNA in Fig. 2b and Supplementary Fig. 10)^34^. Lane 3, which contained the fully assembled DND in the absence of any input, served as the negative control, and all three fluorophores (FAM, Cy3, Cy5) remained efficiently quenched, confirming the intact, OFF‑state DND. In Lanes 4, 5, and 6, where only two of the three requisite inputs were present, we observed the recovery of fluorescence exclusively in the two corresponding channels. Critically, under these sub‑saturating conditions, the tetrahedral head (tFNA) remained tethered to the membrane‑anchoring scaffold (Chol-MAS), as evidenced by the persistence of a single composite band whose color resulted from the additive emission of the two activated fluorophores. These results confirm that any two‑input combination is insufficient to trigger nanostructure disassembly. In Lane 7, the presence of all three inputs induced complete dissociation of the DND. This event is unambiguously demonstrated by the disappearance of the composite band and the concomitant appearance of two new bands whose positions and fluorescence profiles matched precisely those of the isolated tetrahedral head (Lane 1) and scaffold (Lane 2) controls. Together, this stepwise fluorescence readout and band shift pattern provide direct visual and spectroscopic proof of a stringent, three‑input AND‑gated activation mechanism.

To quantitatively validate the orthogonality and absence of signal cross‑talk, we recorded fluorescence spectra for all three channels across every possible input combination. The data confirmed that each output was strictly controlled by a single, designated input: the FAM signal was gated exclusively by input A (Fig. 3d), Cy3 by input B (Fig. 3e), and Cy5 by input C (Fig. 3f). Notably, no significant leaky activation or interference between channels was detected under any other input condition. These data collectively demonstrate that the three-aptamer arms function as independent, parallel‑processing sensors. Their conformational switches are highly specific, and the integrated optical outputs provide a digital‑like, non‑interfering readout that faithfully reports the execution of the intended AND‑logic operation. This in vitro validation establishes the foundational reliability of the DND’s computational core, a prerequisite for its subsequent application in complex biological settings.

### Membrane‑anchored logic‑gated operation enables parallel protein profiling and conditional internalization in target cancer cells

**Fig. 4:**
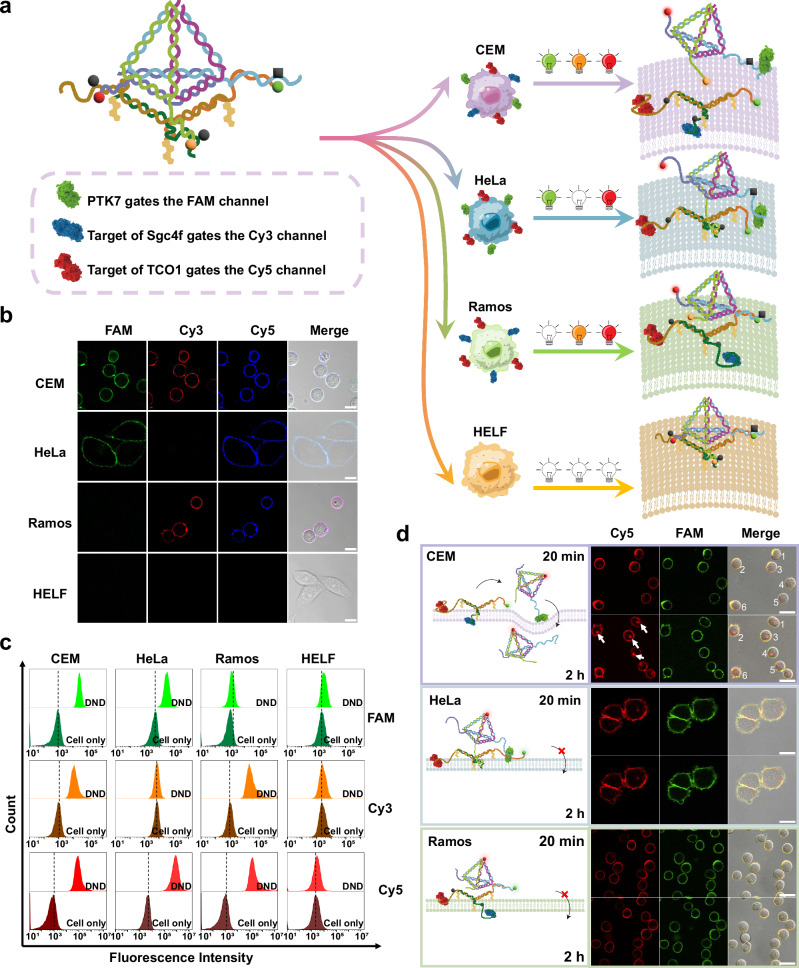
Membrane‑anchored logic‑gated operation enables parallel protein profiling and conditional internalization in target cancer cells. **a** Schematic diagram of the DND responses to different cell lines. **b** CLSM images and **c** flow cytometry histograms showing different fluorescence signals after four cell types were incubated with the DND for 20 min. The experiment was repeated independently three times with similar results. **d** Schematic illustration and CLSM images of the cellular distribution of Cy5-labeled tFNA and FAM-labeled Chol-MAS in different cell types. Cells were incubated with the constructs for 20 min or 2 h before imaging. Arrows indicate representative internalized Cy5-labeled tFNA signals after target-triggered separation, and numbered labels mark representative cells shown in the merged images. The experiment was repeated independently for three times with similar results. Scale bar, 20 μm.

Having validated the AND‑gate mechanism in vitro, we next investigated whether DND could execute its logic‑gated program on living cells to discriminate phenotypes and initiate targeted action. We selected four cell lines that naturally expressed distinct combinations of the three-aptamer targets: CCRF‑CEM (CEM, expressing all three), HeLa (expressing targets of Sgc8c and TCO1), Ramos (expressing targets of Sgc4f and TCO1), and normal human embryonic lung fibroblasts (HELF, negligible expression of all) (Fig. 4a)^25,32^. Prior to logic‑gating experiments, competitive binding assays confirmed that the three aptamers (Sgc8c, Sgc4f, and TCO1) bind to independent, non‑competing membrane receptors, ensuring three orthogonal recognition channels on the same cell (Supplementary Fig. 11). First, we assessed the ability of the DND to perform single‑cell, multiparameter phenotyping. Confocal laser scanning microscopy (CLSM) and flow cytometry revealed cell‑type‑specific fluorescence signatures that precisely mirrored known receptor profiles (Fig. 4b, c). CEM cells exhibited strong FAM, Cy3, and Cy5 signals. HeLa cells presented high FAM and Cy5 but low Cy3 signals, whereas Ramos cells presented high Cy3 and Cy5 but low FAM signals. All three signals remained at background levels in HELF cells. This precise correlation demonstrates that the three fluorescence outputs of the membrane‑anchored DND encode a digital‑like readout of the multitarget status of individual cells, enabling real‑time phenotypic discrimination without external amplification. A key advantage of a programmable platform is its generality. We readily reconfigured the DND by replacing the original aptamers with a new set targeting nucleolin (AS1411), EpCAM, and MUC1 (Supplementary Fig. 12a, b), which are markers abundantly coexpressed on A549 cells but not on the control lines^35–40^. The redesigned DND recapitulated the stringent three-input AND gate behavior: gel electrophoresis confirmed specific disassembly only upon presentation of all three inputs (Supplementary Fig. 12c), and imaging/flow cytometry on A549, HeLa, and HepG2 cells yielded fluorescence signatures that accurately revealed each cell line’s distinct marker profile (Supplementary Fig. 12d, e). This successful reprogramming underscores the platform’s modularity and broad applicability across different biomarker panels and disease models.

The critical functional question was whether this logical reading step was directly coupled to the conditional acting step, namely, the release and internalization of the drug‑loaded module. To visualize this, the tetrahedral head (tFNA) was labeled with Cy5 (red), and the Y‑scaffold (Chol‑MAS) was labeled with FAM (green). Time‑course CLSM imaging revealed a cell‑type‑dependent outcome (Fig. 4d). In CEM cells (full three‑input condition), the FAM and Cy5 signals were initially colocalized at the plasma membrane. After 2 h, a distinct intracellular Cy5 signal emerged, while FAM remained exclusively on the membrane, indicating that the Cy5‑labeled tFNA had dissociated from the membrane‑anchored, FAM‑labeled Chol‑MAS and was internalized. In stark contrast, in HeLa and Ramos cells, which lack one of the required inputs, both the FAM and Cy5 signals remained persistently colocalized at the cell periphery with no significant intracellular Cy5 accumulation, confirming that the nanostructure remained intact and surface‑bound. These results collectively demonstrate that the DND functions as an integrated bio‑inspired nanomachine. It performs parallel, multi‑channel protein profiling on the cell membrane and directly couples this sensing step to spatially controlled actuation: the logic‑gated, conditional release and internalization of its therapeutic cargo. This sense‑and‑act cycle is confined to a single nanostructure, operates without auxiliary amplification or diffusible components, and executes a stringent AND‑gated program that translates complex cellular signatures into specific therapeutic delivery with high fidelity.

### Multiscale analysis of cell‑specific targeting in mixed-cell cohorts: from single‑cell visualization to population‑wide statistical profiling

**Fig. 5:**
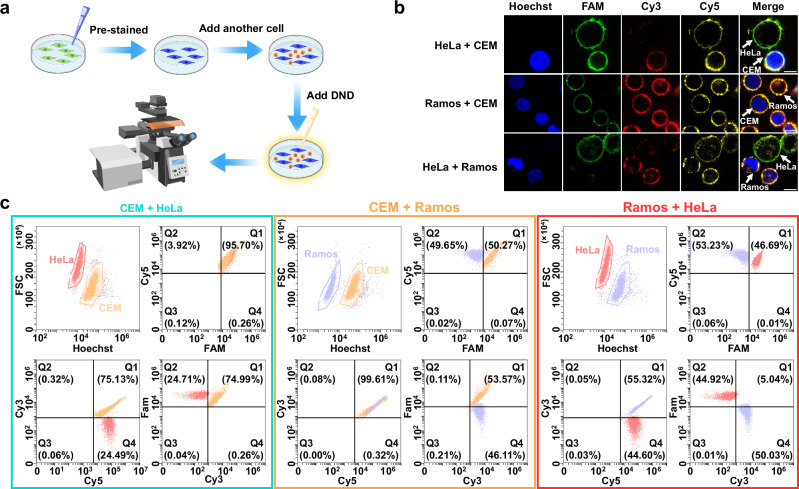
Performance of the DND in complex cell mixtures. **a** Schematic of the confocal imaging assay. The nuclei of one cell type were prestained for identification and mixed with a second cell type, and the resulting cell mixtures were treated with the DND and imaged by CLSM. **b** CLSM images and **c** flow cytometry of mixed cell populations incubated with the DND at 37 °C for 20 min. The experiment was repeated independently for three times with similar results. Scale bar, 10 μm.

After validating the logic-gating mechanism in homogeneous cell lines, we investigated the system’s performance in biologically relevant, heterogeneous environments. The analysis proceeded at multiple scales, from verifying single-cell specificity in mixed populations to population-wide statistical profiling. We first confirmed that each aptamer arm in the fully assembled DND retains its functional independence and selectivity. Competitive binding assays on living cells demonstrated that pre-incubation with an excess of any one free aptamer (e.g., Sgc8c) selectively suppressed only its corresponding fluorescence channel (FAM), while the signals from the other two channels (Cy3 and Cy5) were not affected (Supplementary Fig. 13). This orthogonal blocking pattern, which was consistent across different cell lines, further confirms negligible cross-talk and validates the capacity of the DND for parallel, multi-input recognition on the cell surface. With this molecular specificity verified, we assessed the ability of the DND to discriminate cell types within mixed co-cultures. Following the workflow in Fig. 5a, we pre-stained one cell population with Hoechst 33342, mixed it 1:1 with another unstained population, and incubated the mixture with the DND. Confocal imaging (Fig. 5b) revealed that each cell type maintained its characteristic fluorescence signature with high fidelity and no observable signal transfer between adjacent distinct cells: CEM cells were triple‑positive, HeLa cells were FAM‑/Cy5‑positive, and Ramos cells were Cy3‑/Cy5‑positive. Flow cytometric analysis (Fig. 5c) provided population-level validation. By gating on Hoechst-positive and -negative populations, we could classify cells on the basis solely of their three-channel DND fluorescence, achieving clear separation that matched the known receptor profiles. These multiscale data demonstrate the precise phenotypic discrimination of the DND in complex settings. The observed spatial precision is underpinned by a membrane-confined diffusion trap mechanism^26^. Cholesterol anchoring restricts the intact DND to the membrane of the initial binding cell. Upon AND-gate activation and release, the liberated tFNA, which retains its own targeting aptamer, experiences restricted diffusion within the membrane-adjacent microenvironment. This confinement favors re-binding to receptors on the same cell over escape into the bulk medium, thereby minimizing off-target bystander activation and ensuring that logical computation and therapeutic action are tightly coupled to the correct target cell. This multiscale analysis, spanning from molecular specificity to population‑level statistics, demonstrates that the DND executes precise, logic‑gated cell targeting within heterogeneous mixtures. Quantitative analysis in a mouse blood-cell background further showed reliable detection of low-abundance CEM cells, with the lowest tested quantifiable level being 50 cells per 10,000 analyzed events and recovery values generally ~90% (Supplementary Fig. 14 and Supplementary Table 2).

### Cellular‑level validation of selective cytotoxicity and minimized side effects by the DNA nanodevice

The therapeutic utility of the DND depends on its ability to selectively deliver a cytotoxic payload to target cells. For this purpose, we intercalated the anthracycline drug doxorubicin (Dox) into the GC-rich duplex regions of the tetrahedral module (tFNA), yielding the drug-loaded construct (DND@Dox). Spectral analysis confirmed successful drug loading via significant fluorescence quenching of Dox upon its intercalation into the DNA double helix, a result of the constrained molecular environment and altered electronic interactions within the duplex structure (Supplementary Fig. 15a–d)^41,42^. The assembled DND carrier demonstrated high loading capacity, structural stability, and effective drug retention, which are essential for in vivo applications. Using the fluorescence spectra of free Dox standards and the derived calibration curve (Supplementary Fig. 15e–f), quantitative analysis revealed that each tFNA could load ~30 Dox molecules (Supplementary Fig. 15g). Structurally, compared with its individual components, the DND architecture markedly enhanced nuclease resistance, which is critical for maintaining integrity under physiological conditions (Supplementary Fig. 16). Furthermore, systematic evaluation under three experimental conditions, specifically treatment with excess DNase I, confirmed that the loaded Dox remained stable within the tFNA framework over time, with minimal premature leakage observed (Supplementary Fig. 17). Flow cytometry analysis across concentrations and incubation times indicated that 250 nM DND effectively anchored to the cell membrane within 20 min (Supplementary Fig. 18). However, significant intracellular Dox fluorescence and subsequent apoptosis in target cells required ~2 h of total incubation (Supplementary Fig. 19). This ~100-minute interval represents the essential time for the complete execution of the sense‑and‑act cycle: after anchoring, the DND performs logic‑gated recognition, triggers the dissociation of the drug‑loaded module, facilitates its endocytosis (with trafficking to lysosomes, Supplementary Fig. 20), and culminates in nuclease‑triggered drug release within the cell.

**Fig. 6:**
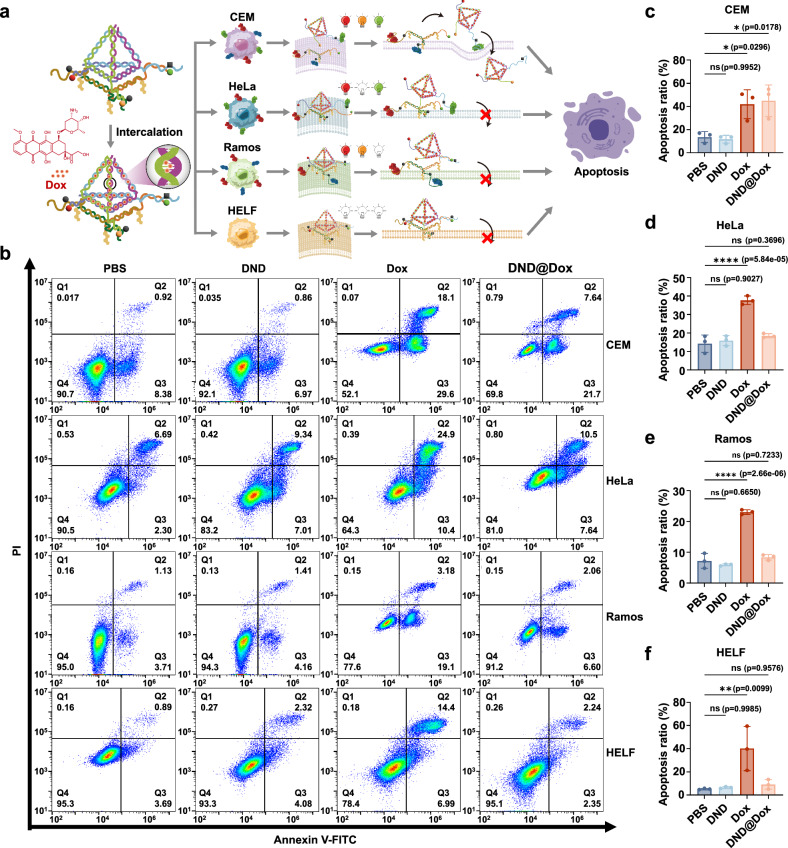
Dox loading and targeted anticancer activity of the DND. **a** Schematic illustration of selective Dox delivery from DND@Dox in target cells. **b** Flow cytometry dot plots of Annexin V-FITC/PI staining for CEM, HeLa, Ramos, and HELF cells after 48 h of treatment with PBS, DND, free Dox, and DND@Dox. **c**–**f** Quantification of apoptosis ratios for CEM (**c**), HeLa (**d**), Ramos (**e**), and HELF (**f**) cells based on the flow cytometry results. Data are presented as mean ± SD (*n* = 3 from three independent cell samples); ns, not significant; *P < 0.05, **P < 0.01, ****P < 0.0001. Statistical significance was evaluated using ordinary one-way ANOVA followed by Tukey’s multiple comparisons test; all tests were two-sided, and *P* values were adjusted for multiple comparisons. Source data are provided as a Source Data file.

Prior to evaluating therapeutic efficacy, we first confirmed the system’s safety; the drug-free carrier (DND) itself demonstrated no appreciable cytotoxicity across various cell lines, and DND@Dox exhibited excellent hemocompatibility, confirming its inherent biocompatibility (Supplementary Fig. 21). We assessed apoptosis and cell viability across four cell lines with distinct receptor profiles (CEM, HeLa, Ramos, and HELF) (Fig. 6a). Free Dox induced high, non-selective toxicity in all cell types (Fig. 6b and Supplementary Fig. 22), and the corresponding Annexin V-FITC/PI apoptosis quantification is summarized in Fig. 6c–f. In stark contrast, DND@Dox induced profound apoptosis and growth inhibition exclusively in CEM cells, which presented the full complement of three target antigens required to trigger the AND gate. Over an extended concentration range, DND@Dox showed a concentration-dependent cytotoxic effect in CEM cells, with an apparent IC_50_ of 29.1 nM, but proved largely insensitive in Ramos, HeLa, and HELF cells under identical conditions (Supplementary Fig. 23). Flow cytometry and confocal imaging confirmed that this selectivity originated from drastically enhanced cellular uptake of DND@Dox only in target CEM cells, while non-target cells showed uptake levels comparable to those of the background (Supplementary Fig. 24). To further examine the structural basis of this selectivity, we compared DND@Dox with two related control constructs, a scaffold-free 3apt-tFNA@Dox and a non-cholesterol 0chol-DND@Dox (Supplementary Fig. 25). In comparison, only cholesterol-anchored DND@Dox showed clear cell selectivity, with uptake mainly observed in cells expressing all three target receptors. The drug-free DND control showed no toxicity in any cell line, confirming that the observed effects are solely cargo-dependent. These data confirm that the DND functions as a logical therapeutic nanomachine. It utilizes its membrane-anchored, multi-input sensing capability to restrict the efficient internalization and potent cytotoxic activity of its payload strictly to target cells that satisfy a predefined molecular signature, thereby achieving maximal therapeutic specificity while minimizing off-target side effects.

### In vivo application of the DNA nanodevice for logic‑gated, low‑toxicity cancer therapy

The ultimate translational potential of the DND lies in its ability to deliver logic-gated therapy in vivo. We first evaluated its efficacy and safety via local (peritumoral) administration in nude mice bearing A549 subcutaneous xenografts. The mice were randomly assigned to five groups: PBS (control), drug-free DND carrier (DND), free doxorubicin (Dox), a non-targeting control (Lib-DND@Dox, with aptamers replaced by random sequences), and the active DND@Dox formulation. All treatments were administered at an equivalent Dox dosage (2 mg/kg) every two days for a total of six doses (Supplementary Fig. 26a). While body weights remained stable across all groups (Supplementary Fig. 26b), only the DND@Dox group exhibited tumor growth inhibition (Supplementary Fig. 26c–f). Histological analysis of the excised tumors confirmed that the most extensive apoptosis and necrosis occurred specifically in the DND@Dox group (Supplementary Fig. 26g). Moreover, examination of major organs revealed no pathological damage in any group, confirming the local biosafety of the platform (Supplementary Fig. 27).

**Fig. 7:**
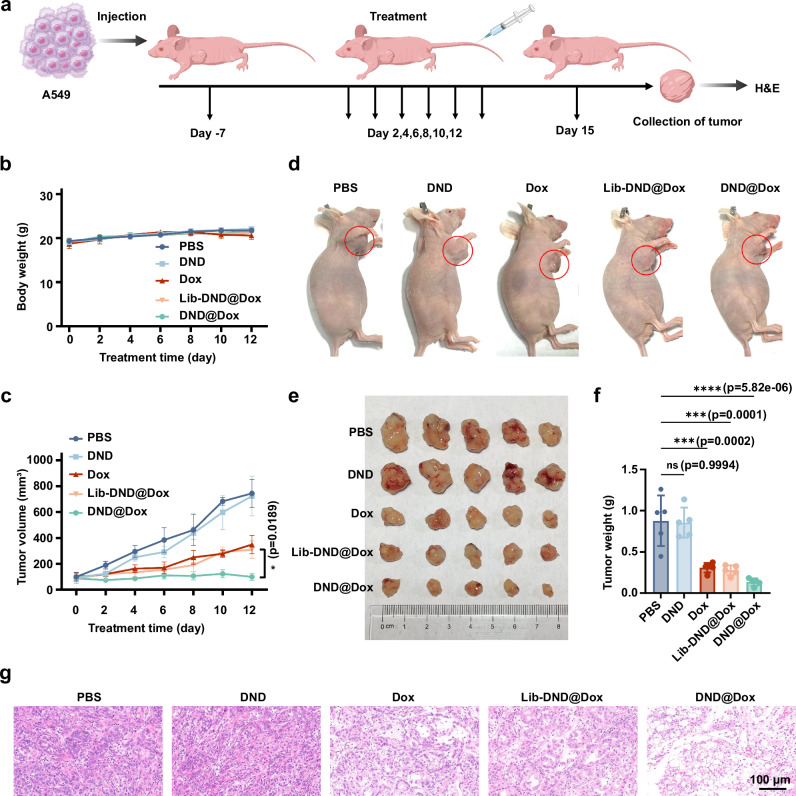
In vivo antitumor efficacy of intravenously administered DND@Dox. ​​​​​​​**a** Overview of the establishment of the A549 cancer model and in vivo treatment scheme. When the tumors reached ~80–100 mm³, the mice were randomized into five groups (*n* = 5 biologically independent mice per group): the PBS, DND, free Dox, DND@Dox, and Lib-DND@Dox groups. Treatments were administered via tail-vein injection every other day for a total of six doses. **b** Changes in body weight of the mice during the treatment period. **c** Tumor growth curves for different treatment groups over the course of therapy. **d** Representative images of tumor-bearing mice from the five groups after six treatments. **e** Photographs of tumors harvested after six treatments. **f** Weights of the harvested tumors. **g** Representative H&E-stained images are shown from five biologically independent mice per group. Scale bar, 100 μm. Data in Fig. 7b, c, f are presented as mean ± SD (*n* = 5 biologically independent mice per group). Statistical significance for Fig. 7c, f was evaluated using ordinary one-way ANOVA followed by Tukey’s multiple comparisons test; all tests were two-sided, and *P* values were adjusted for multiple comparisons. ns, not significant; *P < 0.05, **P < 0.01, ***P < 0.001, ****P < 0.0001. Source data are provided as a Source Data file.

We next investigated the performance of the DND under systemic intravenous administration (Supplementary Fig. 28a). Compared with DND, Lib-DND was cleared more quickly and exhibited a more diffuse distribution. Biodistribution studies using Cy5-labeled constructs revealed that compared with the non-targeting Lib-DND control, the active DND accumulated rapidly and selectively at tumor sites, resulting in stronger and more persistent signals, which were cleared quickly and distributed diffusely (Supplementary Fig. 28b, c). Quantitative analysis of the ex vivo fluorescence distribution in major organs and tumors further showed that the tumor-associated signal accounted for 7.21% of the total ex vivo fluorescence in the Lib-DND group, but increased to 24.10% in the DND group (Supplementary Fig. 28d, e). Using an additional fluorescence-reporting construct in which Cy5 and BHQ2 were placed on the Apt1-lock pair, we further monitored the activation-associated fluorescence kinetics in vivo. The tumor-region fluorescence became detectable at 0.5 h, increased further at 1 h, reached a maximum at ~2 h, and then gradually declined over 4–6 h (Supplementary Fig. 29). This profile highlights the critical role of the aptamer arms in guiding the nanocarrier to the target tissue while minimizing nonspecific organ retention. Finally, we assessed the therapeutic outcome of systemic treatment following the regimen shown in Fig. 7a. Compared with the mice in the group receiving free Dox, the mice in the group receiving DND@Dox maintained stable body weights and did not exhibit detectable weight loss, underscoring the role of the carrier in mitigating systemic toxicity (Fig. 7b). In terms of anti-tumor efficacy, DND@Dox resulted in the most potent suppression of tumor growth, significantly outperforming PBS, the drug-free DND, free Dox, and the non-targeting Lib-DND@Dox control (Fig. 7c–f). The weaker tumor inhibition observed after intravenous administration relative to peritumoral treatment likely reflects the additional delivery barriers encountered under systemic administration before the nanostructures reach the tumor site^3,43,44^. Nevertheless, DND@Dox still retained significant anti-tumor activity after intravenous administration. Histological analysis of the endpoint tumors corroborated these results, with the most severe cellular damage occurring exclusively in the DND@Dox group (Fig. 7g). A comprehensive safety assessment was conducted post-treatment. Histopathological examination of major organs (heart, liver, spleen, lung, and kidney) revealed no evidence of damage or inflammatory infiltration in the DND@Dox group (Supplementary Fig. 30). In contrast, signs of liver inflammation were observed in both the free Dox group and the Lib-DND@Dox group. Serum biochemical analysis further revealed this difference: the levels of liver enzymes (ALT and AST) were significantly elevated only in the free Dox group, while all the parameters in the DND@Dox group remained within normal ranges and were indistinguishable from those in the PBS control group (Supplementary Fig. 31). The non-targeting Lib-DND@Dox group showed a milder, intermediate toxicity profile. The in vivo data demonstrate that the DND achieves logic-guided tumor targeting, resulting in significantly enhanced therapeutic efficacy and a markedly improved safety profile compared to both free drug and non-targeted nanocarriers. These findings validate the DND as a promising platform for intelligent, low-toxicity cancer therapy.

## Discussion

We report a membrane-anchored DND that integrates multivalent sensing, Boolean logic computation, and conditional drug delivery within a single nanostructure. Its three allosteric aptamer arms perform parallel, logic-gated profiling of cell surface markers, directly triggering the localized release of a doxorubicin-loaded tetrahedral module only upon satisfying a stringent, three-input AND gate. This sense and act cycle is spatially confined via cholesterol anchoring and a diffusion trap mechanism, ensuring high fidelity without free diffusing components. The DND performs precise phenotypic discrimination, selectively inducing apoptosis only in target cells (e.g., CEM) within mixed populations while sparing nontarget cells (e.g., HeLa, Ramos, HELF). This design addresses a key limitation of single-receptor targeting, namely its limited ability to exclude nontarget cells with partially overlapping marker expression. In vivo, compared with nontargeting controls, the aptamer-guided DND@Dox formulation exhibited enhanced tumor targeting, as evidenced by its superior accumulation at tumor sites and reduced nonspecific distribution. This led to potent tumor growth inhibition in both local and systemic administration models while significantly mitigating the systemic toxicity (e.g., weight loss and hepatotoxicity) associated with free doxorubicin. The platform is inherently modular; its targeting specificity and therapeutic logic can be rapidly reprogrammed, as evidenced by successful retargeting to an alternative biomarker panel (nucleolin/EpCAM/MUC1). By unifying multivalent recognition with spatially controlled drug delivery, the DND establishes a versatile and adaptive paradigm for logic-guided cancer theranostics.

## Methods

### Materials and reagents

Supplementary Table 1 summarizes all the oligonucleotides used in this study, which were synthesized, modified, and purified by Shanghai Sangon Biological Engineering Technology and Services Co., Ltd. (Shanghai, China). Upon receipt, the oligonucleotides were reconstituted in 1× TE buffer (10 mM Tris-HCl, 1 mM EDTA, pH 8.0), quantified using a NanoDrop 2000 spectrophotometer (Thermo Fisher Scientific, USA), and adjusted to 10 μM for subsequent experiments. All DNA solutions were stored at 4 °C until use. For gel electrophoresis, a low molecular weight DNA ladder (New England Biolabs, Beijing, China) was used as the molecular size standard. SYBR Green I and Hoechst 33342 were purchased from Solarbio (Beijing, China). DNase I and DiI (C1036) were obtained from Beyotime (Shanghai, China), and doxorubicin (Dox) was obtained from Sangon Biotech (Shanghai, China). Fetal bovine serum (FBS), Dulbecco’s modified Eagle medium (DMEM), and RPMI-1640 were purchased from Gibco (Thermo Fisher Scientific, USA), and the Cell Counting Kit-8 (CCK-8) assay kit was purchased from Thermo Fisher Scientific (USA). BALB/c nude mice were supplied by Vital River Laboratory Animal Technology Co., Ltd. (Zhejiang, China). Paraffin embedding, sectioning, and hematoxylin and eosin (H&E) staining of tumor tissues and major organs were performed by Shanghai Lianlan Biotechnology Co., Ltd. (Shanghai, China).

### Preparation of the DND

Assembly of tFNA: Equimolar amounts (1 μL, 10 μM) of four DNA strands (t-s1, t-s2, t-s3, and t-s4) were mixed with 0.5 μL of 10× TM buffer (100 mM Tris, 12.5 mM MgCl_2_, pH 8.0) in a 0.5-mL microcentrifuge tube, and the final volume was adjusted to 5 μL with ddH2O. The mixture was heated to 95 °C for 10 min and then cooled to 4 °C for 2 h to allow for self-assembly of the DNA tetrahedron.

Construction of the Chol-MAS: Similarly, 1 μL (10 μM) of each Y-s1, Y-s2, and Y-s3 strand was mixed with 0.5 μL of 10× TM buffer in a fresh 0.5-mL microcentrifuge tube, and the total volume was adjusted to 5 μL with ddH2O. The mixture was heated to 95 °C for 10 min and then cooled to 4 °C for 2 h to form the Y-shaped DNA scaffold.

Assembly of DND: Equal volumes (5 μL each) of the preannealed tFNA and Chol-MAS solutions were mixed and incubated at 37 °C for 2 h to allow for hybridization between the complementary sticky ends, yielding the integrated DND.

### Gel electrophoresis analysis

Agarose gel electrophoresis (AGE) was performed to analyze the assembly and target responsiveness of DND. For each sample, 8 μL of the reaction mixture was combined with 2 μL of 6 × loading buffer and 2 μL of SYBR Green I nucleic acid stain prior to loading. The DNA marker was prepared by mixing 8 μL of ddH2O, 2 μL of 6× loading buffer, 2 μL of SYBR Green I, and 0.1 μL of a low-molecular-weight DNA ladder. Electrophoresis was performed in 0.5× TBE buffer (45 mM Tris-borate, 1 mM EDTA, pH 8.3). For assembly analysis and target responsiveness analysis, 3% and 2% agarose gels were used, respectively. All gels were run at 100 V for 40 min. After electrophoresis, the gels were imaged using a ChemiDoc XRS imaging system (Bio-Rad, USA). Uncropped and unprocessed gel images are provided in the Source Data file.

### AFM characterization

The DND structure and its individual components were prepared as described above. For AFM imaging, each sample (5 nM, 50 μL) was deposited onto freshly cleaved mica pretreated with 3-aminopropyltriethoxysilane (APTES, Sigma-Aldrich) and incubated for 5 min at room temperature. The samples were then washed with TE buffer containing 5 mM MgCl2 (TE-MgCl2). AFM images were acquired in AC water topography mode using an ultra-sharp silicon probe (BL-AC40TS, Olympus) on a Cypher-VRS AFM system (Oxford Instruments) under ambient conditions.

### Cryogenic transmission electron microscopy (cryo-TEM)

The samples were prepared according to the procedure described in the Preparation of the DND section. For cryo-TEM imaging, C02-Cu300-1.2/1.3 grids were glow-discharged for hydrophilization using a Ted Pella easiGlow system and immediately used for vitrification with a Leica EM GP2 plunge freezer. A 3 μL aliquot of each sample was applied to the grid, blotted for 5 s, and rapidly plunge-frozen in liquid ethane at ~−182 °C. The vitrified grids were transferred under liquid nitrogen to a Gatan 698 cryo-holder and loaded into a JEOL JEM-F200 transmission electron microscope operated at 200 kV. Imaging was performed under low-dose conditions at approximately −175 °C.

### Fluorescence response assay

To evaluate the fluorescence activation of the DND upon target recognition, the nanostructure was first prepared as described in the Preparation of the DND section. Afterward, 1 μL (10 μM) of each complementary strand (C-TCO1, C-Sgc4f, and C-Sgc8c), which mimics the specific binding of the aptamers to their respective protein targets, was added to the assembled DND solution. Subsequently, 1× TM buffer (10 mM Tris, 1.25 mM MgCl₂, pH 8.0) was added to each tube to bring the total volume to 20 μL. The mixtures were incubated at 37 °C in a thermostatic metal bath for 1 h to allow for complete hybridization. After incubation, 180 μL of 1× TM buffer was added to each tube, and the solutions were gently vortexed to ensure thorough mixing. Fluorescence spectra were then recorded on an F-7000 fluorescence spectrophotometer (Hitachi Ltd., Japan) with quartz cuvettes. Excitation wavelengths were set at 492 nm for FAM, 550 nm for Cy3, and 646 nm for Cy5. Fluorescence intensity is reported in arbitrary units.

### Serum stability assay

DND and related components were prepared as described previously. A mixture containing 2 μL of FBS and 10 μL of the DND was brought to a final volume of 20 μL with 1× TM buffer. The samples were incubated at 37 °C for various durations (0, 2, 4, 6, 8, 12, and 24 h). After incubation, the structural integrity of the nanostructures was evaluated by 2% agarose gel electrophoresis. The untreated sample (0 h) served as the control. Band intensities were quantified using ImageJ 1.54 to assess structural stability over time.

### Cell culture

CEM, Ramos, HeLa, A549, HepG2, and HELF cells were obtained from the Institute of Biochemistry and Cell Biology, Chinese Academy of Science (Shanghai, China). IOSE80 cells were obtained from Shanghai Fuheng Biotechnology Co., Ltd. CEM, Ramos and IOSE80 cells were cultured in RPMI-1640 medium supplemented with 10% FBS and 1% penicillin‒streptomycin. HeLa, HELF and HepG2 cells were maintained in DMEM supplemented with 10% FBS and 1% penicillin‒streptomycin. A549 cells were cultured in F-12K medium supplemented with 10% FBS and 1% penicillin–streptomycin. All the cells were incubated at 37 °C in 5% CO₂.

### DND membrane anchoring assay by CLSM

To evaluate the membrane anchoring ability of the DND, HeLa and HELF cells (1 × 10⁵) were seeded into 24-well plates preloaded with sterile coverslips and cultured for at least 24 h. The cells were then incubated with 250 nM FAM-labeled DND (labeled on the t-s4 strand) in serum-free DMEM at 37 °C for 20 min. After incubation, the cells were washed three times with 1× PBS and fixed with 4% paraformaldehyde for 15 min. The coverslips were then gently removed and mounted face-down onto glass slides for CLSM imaging. For suspension cells (CEM and Ramos), 1 × 10⁵ cells were incubated with 250 nM FAM-labeled DND under the same conditions, fixed with 4% paraformaldehyde, and transferred to glass-bottomed confocal dishes for imaging. All the images were acquired using a confocal laser-scanning microscope (Nikon, Japan).

### Membrane colocalization of DND with DiI

To investigate the membrane colocalization of the DND, Ramos cells were prestained with the membrane dye DiI at 37 °C for 15 min, washed with PBS, and then incubated with 250 nM FAM-labeled DND for 20 min. After being washed, the cells were imaged to assess colocalization between the nanostructures and the membrane. Colocalization analysis was performed using ImageJ software.

### FRAP analysis

Ramos cells were incubated with 250 nM FAM-labeled DND and subjected to FRAP analysis by CLSM. A defined membrane region (Region #1) was photobleached using a 405 nm laser at 100% intensity for 5 s, while another membrane region (Region #2) was left unbleached as the reference. Fluorescence images were acquired before bleaching, immediately after bleaching, and during recovery up to 155 s. The fluorescence intensity of Region #1 was normalized against the unbleached control region (Region #2) to correct for background photobleaching.

### DNase I validation of surface-localized DND

To confirm the outer-membrane localization of the DND, Ramos cells incubated with 250 nM FAM-labeled DND were imaged by CLSM before and after treatment with DNase I (1 U/μL) at 37 °C for 10 min.

### Cell response to the DND

For flow cytometry, CEM, Ramos, HeLa, and HELF cells were incubated with 250 nM DND labeled with FAM, Cy3, and Cy5 in serum-free medium at 37 °C for 20 min, washed with 1× PBS, and analyzed via a CytoFLEX S flow cytometer (Beckman Coulter, USA). A total of 10,000 events were collected per sample. The fluorescence was excited at 488 nm, 561 nm, and 638 nm, and the signals were recorded in the corresponding channels. For CLSM, HeLa and HELF cells on coverslips and CEM and Ramos suspension cells were incubated with 250 nM multicolor-labeled DND under the same medium conditions for 20 min, fixed with 4% paraformaldehyde, and imaged by confocal microscopy (Nikon, Japan).

### Aptamer binding competition assay

To confirm that different aptamers recognize their targets independently without mutual interference, CEM cells were first incubated with 5 μM unlabeled aptamer at 37 °C for 30 min to block the corresponding target. Subsequently, a Cy5-labeled aptamer targeting a different marker was added, and the cells were incubated for another 30 min. As a control, the cells were directly incubated with the Cy5-labeled aptamer without prior blocking. After incubation, the cells were washed twice with 1× PBS and analyzed by flow cytometry to compare the fluorescence intensities between the blocked and unblocked groups.

### Cell type-selective disassembly assay

To investigate the selective disassembly of the DND in response to different cell types, DND constructs comprising Cy5-labeled tFNA and FAM-labeled Chol-MAS were incubated with various cell types at 250 nM for 2 h. CLSM imaging was then performed to evaluate nanostructure disassembly triggered by cell-specific molecular recognition.

### Competitive binding assay for DND recognition

To verify the competitive binding specificity of each aptamer in the DND system, the cells were first blocked by incubation with 5 μM unlabeled aptamers at 37 °C for 30 min to saturate the corresponding binding sites. After being washed with 1× PBS, the cells were incubated with 250 nM DND structures labeled with FAM, Cy3, and Cy5 for an additional 30 min. Flow cytometry was performed using a CytoFLEX S flow cytometer (Beckman Coulter, USA), and 10,000 events were recorded for each sample. Fluorescence excitation was provided by three independent lasers at 488 nm (FAM), 561 nm (Cy3), and 638 nm (Cy5), and the corresponding emission signals were collected through detector channels matched to each excitation wavelength.

### Cell type-specific recognition of the DND in mixed cell populations

To evaluate cell type-specific recognition in a mixed population, HeLa, CEM, and Ramos cells were tested as pairwise mixtures. For each pair, one cell type was prestained with Hoechst 33342 (1 μg/mL) at 37 °C for 15 min for nuclear labeling. After three washes with 1× PBS, the labeled cells were combined with the unlabeled counterpart and incubated with DND (250 nM) in serum-free medium at 37 °C for 30 min. The cells were subsequently washed three times with 1× PBS and then subjected to flow cytometry for quantitative analysis and CLSM for imaging after they were seeded in glass-bottomed dishes.

### Dox loading and release from tFNA

Tetrahedral DNA nanostructures (tFNAs) were assembled as described above. For loading assays, tFNA was mixed with Dox to a final Dox concentration of 2 μM in a total reaction volume of 20 μL using 1× TM buffer. The tFNA concentration was varied as indicated (0–250 nM). The samples were incubated at 37 °C for 8 h to allow for Dox intercalation. To optimize the loading time, 100 nM tFNA was incubated with Dox (2 μM) at 37 °C for different durations. After incubation, each sample was diluted with 1× TM buffer to 200 μL for fluorescence measurement. Fluorescence spectra were recorded via an F-7000 fluorescence spectrophotometer (Hitachi, Japan) with excitation at 488 nm and emission scanning from 510 to 750 nm.

To quantify Dox loading, a calibration curve was constructed by measuring the fluorescence intensity of free Dox standards prepared in the same buffer and measured under identical conditions. The concentration of free Dox in each loaded sample was calculated from the calibration curve. The amount of Dox loaded into the tFNA was calculated by mass balance:

1 $${C}_{{{{\rm{Dox}}}},{{{\rm{loaded}}}}}={C}_{{{{\rm{Dox}}}},{{{\rm{total}}}}}-{C}_{{{{\rm{Dox}}}},{{{\rm{free}}}}}$$

The loading ratio is expressed as the number of Dox molecules per tFNA, with all concentrations converted to consistent units:

2 $${N}_{{{{\rm{Dox}}}}/{{{\rm{tFNA}}}}}=\frac{{C}_{{{{\rm{Dox}}}},{{{\rm{loaded}}}}}}{{C}_{{{{\rm{tFNA}}}}}}$$

For release analysis, tFNA and Dox were mixed at a molar ratio of 1:30 under the optimized loading conditions and incubated at 37 °C for 8 h to form tFNA@Dox. The complexes were then incubated with DNase I (20 U/mL) or DNase-free buffer at 37 °C. Fluorescence spectra were collected at the indicated time points using the same instrument settings to monitor Dox release associated with enzymatic degradation.

### Cell-selective delivery of DND@Dox

To evaluate cell-selective delivery, HeLa, Ramos, and CEM cells were incubated with DND@Dox (70 nM) or free Dox (2 μM) at 37 °C for 2 h in serum-free medium. The cells were then rinsed three times with 1× PBS, followed by flow cytometry analysis to quantify the intracellular Dox fluorescence in the FITC channel and CLSM imaging to visualize cellular Dox accumulation.

### Cell viability assay

Cells (8 × 10³ per well) were seeded into 96-well plates, incubated with different materials for 6 h, washed to remove unbound materials, and then cultured in fresh medium for an additional 48 h. After treatment, the medium was discarded, and 100 μL of Cell Counting Kit-8 working solution (1:10 dilution in serum-free medium) was added to each well. The plates were incubated at 37 °C for 1 h, after which the absorbance at 450 nm was measured using a multifunctional microplate reader. Cell viability was calculated according to the manufacturer’s instructions.

### Hemolysis assay

Whole blood from nude mice was centrifuged at 400 × *g* for 5 min, the plasma was discarded, and the red blood cells (RBCs) were washed three times with 0.9% (w/v) NaCl solution (normal saline). The washed RBCs were resuspended in normal saline to obtain a 4% (v/v) stock, which was mixed 1:1 with DND@Dox solutions of various concentrations to yield 2% (v/v) RBC suspensions and incubated at 37 °C for 4 h. H2O and normal saline served as positive and negative controls, respectively. After incubation, the samples were centrifuged at 400 × *g* for 5 min, after which the absorbance of the supernatant at 541 nm was recorded. Hemolysis was calculated using the following equation:

3 $${{{\rm{Hemolysis}}}}(\%)=\frac{{A}_{{{{\rm{sample}}}}}-{A}_{{{{\rm{negative}}}}}}{{A}_{{{{\rm{positive}}}}}-{A}_{{{{\rm{negative}}}}}}\times 100\%$$

### Animal experimentation

All animal experiments were performed under the protocol approved by the Laboratory Animal Ethics Committee of Wenzhou Medical University (approval number: wydw2024-0453). BALB/c nude mice (Mus musculus, BALB/c genetic background, 3–4 weeks old) were obtained from Zhejiang Vital River Laboratory Animal Technology Co., Ltd. (Zhejiang, China). Mice were housed under specific pathogen-free conditions at an ambient temperature of 25 °C and a relative humidity of 40–60%, with a 12 h light/dark cycle.

A549 tumor xenografts were established by subcutaneous injection of 100 μL of A549 cell suspension (1 × 10⁶ cells) into the axillary region of 3–4-week-old BALB/c nude mice. When the tumor volume reached approximately 80–100 mm³, the mice were randomly divided into five groups: (1) PBS, (2) DND, (3) free Dox, (4) DND@Dox, and (5) Lib-DND@Dox. For groups (3)–(5), treatments were formulated to deliver an equivalent Dox dose of 2 mg/kg. DND@Dox and Lib-DND@Dox were prepared by loading Dox onto the DND at a 1:30 molar ratio. Each experimental condition was tested using both peritumoral and tail-vein injection routes, with all other parameters kept identical. The PBS group received the same volume of PBS, and the DND group received the same nanostructure concentration and volume as the DND@Dox group but without Dox. The tumor volume and body weight were recorded every two days, and the tumor volume was calculated as follows:

4 $$V=\frac{L\times {W}^{2}}{2}$$

where *V* represents the tumor volume, *L* represents the tumor length, and *W* represents the tumor width.

At the end of the treatment period, the tissues were collected, fixed, embedded, and sectioned for H&E staining to examine histopathological changes. In parallel, serum biochemical parameters, including alanine aminotransferase, aspartate aminotransferase (AST), blood urea nitrogen, and creatinine, were measured using commercial assay kits to evaluate the potential effects of the administered materials on hepatic and renal function in nude mice.

### In vivo imaging

Nude mice bearing A549 xenografts were randomly assigned to two groups. Cy5 was conjugated to the t-s4 strand for fluorescence tracking, and DND constructs were assembled as described in the Preparation of the DND section. As a sequence control, Lib-DND was generated by replacing the aptamer sequence with a randomized sequence of the same length while keeping all the other strands, stoichiometries, and assembly conditions identical. The mice received an intravenous injection of Cy5-labeled DND or Lib-DND (10 μM in 100 μL per mouse). Whole-body fluorescence imaging was performed on an IVIS Spectrum system (PerkinElmer, USA) at 1, 2, 4, and 6 h post-injection. For ex vivo analysis, major organs were harvested at 2 h after tail-vein administration and imaged under identical conditions. Cy5 was excited at 640 nm, and the emission was measured at 680 nm. The fluorescence signals were quantified using Living Image 4.5.5.

### Reporting summary

Further information on research design is available in the Nature Portfolio Reporting Summary linked to this article.

## Supplementary information

Supplementary Information

Reporting Summary

Transparent Peer Review file

## Source data

Source data

## Data Availability

The data generated or analyzed during this study are included in this article, the Supplementary Information, and the Source Data file. Source data are provided for Figs. 2b–e, h, j, 3c–f, 6c–f, 7b, c, e–g and Supplementary Figs. 4, 6, 10, 12c, 14, 15b–g, 16, 17b, 19–23, 26b, d–g, 27, 28c, e, 29–31.  Source data are provided with this paper.
