# Supplementary material for: Membrane-anchored DNA nanodevice with allosteric aptamer arms enables parallel probing and on-site drug delivery
Source: Nat Commun. 2026 Jul 7;17:8394. doi: 10.1038/s41467-026-75300-5 (PMC13473122; doi:10.1038/s41467-026-75300-5)
Supplement: Supplementary file 1 [file 41467_2026_75300_MOESM1_ESM.pdf]

Supplementary Information for

Membrane-Anchored DNA Nanodevice with Allosteric Aptamer Arms Enables Parallel Probing and On-Site Drug Delivery

Table of Contents

Section A: Supplementary experimental procedures 4

Optimization of DND membrane anchoring 4

Serum stability assay of membrane anchored DND 4

Time-dependent uptake of free Dox and DND@Dox 4

Lysosomal trafficking analysis of DND 5

Comparative analysis of cellular localization and uptake by CLSM 5

Quantitative evaluation of CEM cell detection in a mouse blood-cell background 5

In vivo fluorescence imaging of DND activation in tumors 6

Computational modeling and simulation 6

Section B: Supplementary figures 8

Supplementary Figure 1. Nucleotide sequences of the DND. 8

Supplementary Figure 2. Exploratory structural models of the DND before and after PTK7 docking... 9

Supplementary Figure 3. Exploratory free-binding simulation of Sgc8c with PTK7. 10

Supplementary Figure 4. Simplified sgc8c–lock–PTK7 model for evaluating local aptamer–lock coupling. 11

Supplementary Figure 5. Theoretical size estimation of tFNA, Chol-MAS, and DND. 12

Supplementary Figure 6. Representative cryo-electron microscopy (Cryo-EM) micrographs of DND and its structural modules. 14

Supplementary Figure 7. Serum stability of membrane anchoring for different DND variants. 15

Supplementary Figure 8. Membrane anchoring of DND in different cell lines. 16

Supplementary Figure 9. DNase I validation of surface-localized DND. 17

Supplementary Figure 10. Agarose gel analysis of target-responsive DND disassembly. 18

Supplementary Figure 11. Aptamer binding competition assay in CEM cells. 19

Supplementary Figure 12. Logic operation of the second DND system in different cell lines. 20

Supplementary Figure 13. Competitive binding assay for DND recognition. 21

Supplementary Figure 14. Quantitative evaluation of CEM cell detection in a mouse blood-cell background. 23

Supplementary Figure 15. Characterization of Dox loading into tFNA. 25

Supplementary Figure 16. Serum stability of DND and its components. ... 27

Supplementary Figure 17. DNase I-triggered drug release from tFNA@Dox. ... 28

Supplementary Figure 18. Optimization of DND membrane anchoring. ... 29

Supplementary Figure 19. Cellular uptake kinetics of free Dox and DND@Dox in CEM cells. ... 30

Supplementary Figure 20. Lysosomal trafficking analysis of DND. ... 31

Supplementary Figure 21. In vitro biocompatibility of DND and DND@Dox. ... 32

Supplementary Figure 22. Cytotoxicity of DND@Dox across different cell types. ... 33

Supplementary Figure 23. Cell viability over an extended concentration range of DND@Dox. ... 34

Supplementary Figure 24. Cell-selective delivery of DND@Dox. ... 35

Supplementary Figure 25. Comparative study of the cellular localization and uptake of DND@Dox and related control constructs in different cell lines. ... 36

Supplementary Figure 26. Peritumoral therapy in A549 tumor-bearing mice. ... 37

Supplementary Figure 27. H&E staining of major organs after peritumoral treatment. ... 39

Supplementary Figure 28. Biodistribution of DND and Lib-DND after tail-vein injection. ... 40

Supplementary Figure 29. In vivo fluorescence reporting of tumor-associated activation behavior of DND. ... 41

Supplementary Figure 30. H&E staining of major organs after intravenous treatment. ... 42

Supplementary Figure 31. Serum biochemical indices of mice after intravenous treatment. ... 43

Section C: Supplementary tables ... 44

Supplementary Table 1. The sequences of oligonucleotides used in this work [a)]. ... 44

Supplementary Table 2. Quantitative parameters for CEM cell detection in a mouse blood-cell background. ... 47

Section D: Uncropped gels ... 48

Section E: Supplementary References ... 49

Section A: Supplementary experimental procedures

Optimization of DND membrane anchoring

To optimize the membrane anchoring efficiency of the DND, Ramos cells were incubated with FAM-labeled DND at various concentrations (0–350 nM) for 20 min, or with 250 nM DND for different incubation times (0–60 min), at 37 °C. After incubation, cells were washed twice with 1 × PBS and analyzed on a CytoFLEX flow cytometer (Beckman Coulter, USA). A total of 10,000 events were collected for each sample.

Serum stability assay of membrane anchored DND

To assess the anchoring stability of DND constructs bearing different numbers of cholesterol moieties, FAM-labeled DND structures (t-s4-modified) containing 0, 1, 2, or 3 cholesterol units were incubated with Ramos cells in RPMI-1640 medium supplemented with 10% fetal bovine serum (FBS) at 37 °C for various time intervals. After incubation, the cells were washed three times with 1 × PBS to remove unbound nanostructures and then transferred into glass-bottom confocal dishes for CLSM imaging.

Time-dependent uptake of free Dox and DND@Dox

To evaluate the time-dependent cellular internalization of free Dox and DND@Dox, CEM cells were incubated with 2 μM free Dox or 70 nM DND@Dox in serum-free medium at 37 °C for various time intervals. After incubation, the cells were washed three times with 1 × PBS to remove unbound drug or nanostructures, and resuspended in PBS for flow cytometry analysis. Dox fluorescence was detected in the FITC channel under 488 nm excitation on a CytoFLEX S flow cytometer (Beckman Coulter, USA), and 10,000 events were collected for each sample.

Lysosomal trafficking analysis of DND

To assess the intracellular trafficking of the DND, CEM cells were incubated with 250 nM FAM-labeled DND in serum-free RPMI-1640 medium at 37 °C for 2 h. After washing three times with 1 × PBS, the cells were further incubated with Lyso-Tracker Red (Beyotime, China) at 37 °C for 1 h to stain lysosomes. Excess dye was removed by washing the cells 3 times with 1 × PBS. The cell suspension was then transferred into a glass-bottom confocal dish for CLSM imaging.

Comparative analysis of cellular localization and uptake by CLSM

CEM, HeLa, Ramos, HELF, and IOSE80 cells were incubated with 3apt-tFNA@Dox, 0chol-DND@Dox, or DND@Dox at a final concentration of 70 nM at 37 °C for 2 h. IOSE80 cells were included as a normal human ovarian epithelial cell line with reported PTK7 expression to further evaluate selectivity in a normal-cell background[1]. After incubation, the cells were washed three times with 1 × PBS to remove unbound nanostructures and then subjected to confocal laser scanning microscopy. Cy5 fluorescence was collected in the Cy5 channel to track the nanostructures, whereas the intrinsic fluorescence of Dox was collected in the FITC channel to monitor drug localization and uptake.

Quantitative evaluation of CEM cell detection in a mouse blood-cell background

Mouse whole blood was collected, and plasma was removed by centrifugation at 400 × *g* for 10 min. The resulting blood cells were washed with PBS, resuspended, and counted. Hoechst 33342-prelabeled CEM cells were then spiked into the washed mouse blood-cell suspensions at predetermined ratios. After incubation with Cy5/BHQ2-labeled DND for 30 min, the samples were washed and analyzed by flow cytometry. Hoechst-positive cells were identified in the PB450 channel,

and DND recognition was assessed in the Cy5 channel. For each sample, 10,000 events were collected.

In vivo fluorescence imaging of DND activation in tumors

Nude mice bearing A549 xenografts were randomly assigned to two groups. For fluorescence reporting, Cy5 was labeled on the Apt1 strand and BHQ2 was labeled on the corresponding lock strand, and the DND constructs were assembled as described in the Preparation of DND section. As a sequence control, Lib-DND was generated by replacing the aptamer sequence with a randomized sequence of the same length, while keeping all other strands and assembly conditions identical. Mice received DND or Lib-DND via tail-vein injection (10 μM in 100 μL per mouse). Whole-body fluorescence imaging was performed on an IVIS Spectrum system (PerkinElmer, USA) at 0, 0.5, 1, 2, 4, and 6 h post-injection. Cy5 was excited at 640 nm and emission was collected at 680 nm.

Computational modeling and simulation

For the PTK7-free DND model, the initial DND scaffold was generated using oxDNA according to the designed DNA sequences, base-pairing patterns, strand connectivity, and overall spatial arrangement[2]. The generated model was converted into atomic coordinates and subsequently energy-minimized using GROMACS 2024.2[3]. Energy minimization was performed with the steepest descent algorithm to remove unfavorable contacts and obtain a relaxed DND scaffold. For the PTK7-bound DND model, the structure of Sgc8c was taken from the reported NMR-resolved structure (PDB ID: 8Y0F)[4]. The PTK7 Ig3–Ig4 domain was modeled using AlphaFold 3 and used as the target-binding domain according to the reported Sgc8c-binding region[5]. Sgc8c was docked to PTK7 using HDOCK[6-8], and the resulting Sgc8c–PTK7 complex was positioned on the designated tFNA arm within the

DND architecture. The full DND–PTK7 model was then subjected to structural relaxation using GROMACS 2024.2 to reduce steric clashes and optimize the local geometry[3].

To examine the early encounter between Sgc8c and PTK7, three independent unrestrained simulations were performed, with Sgc8c initially placed approximately 9 Å away from the PTK7 Ig3–Ig4 domain. Representative snapshots were extracted to visualize the time-dependent Sgc8c–PTK7 interaction.

To evaluate local aptamer–lock coupling, a simplified sgc8c–lock–PTK7 model was constructed. The Sgc8c 3WJ tertiary core was preserved based on the NMR-resolved structure, and the lock strand was simplified to a minimal 6 bp complementary segment. The PTK7 Ig3–Ig4 domain was included as the target-binding domain. The system was simulated for 100 ns in explicit solvent using GROMACS. DNA and protein were described using the OL21 and ff19SB force fields, respectively. Hydrogen bonds between the terminal DA33–DT9 base pair of the lock duplex were monitored along the trajectory. MM/GBSA analysis was performed to estimate the binding free energy of the lock–sgc8c interaction in the absence and presence of PTK7.

Section B: Supplementary figures

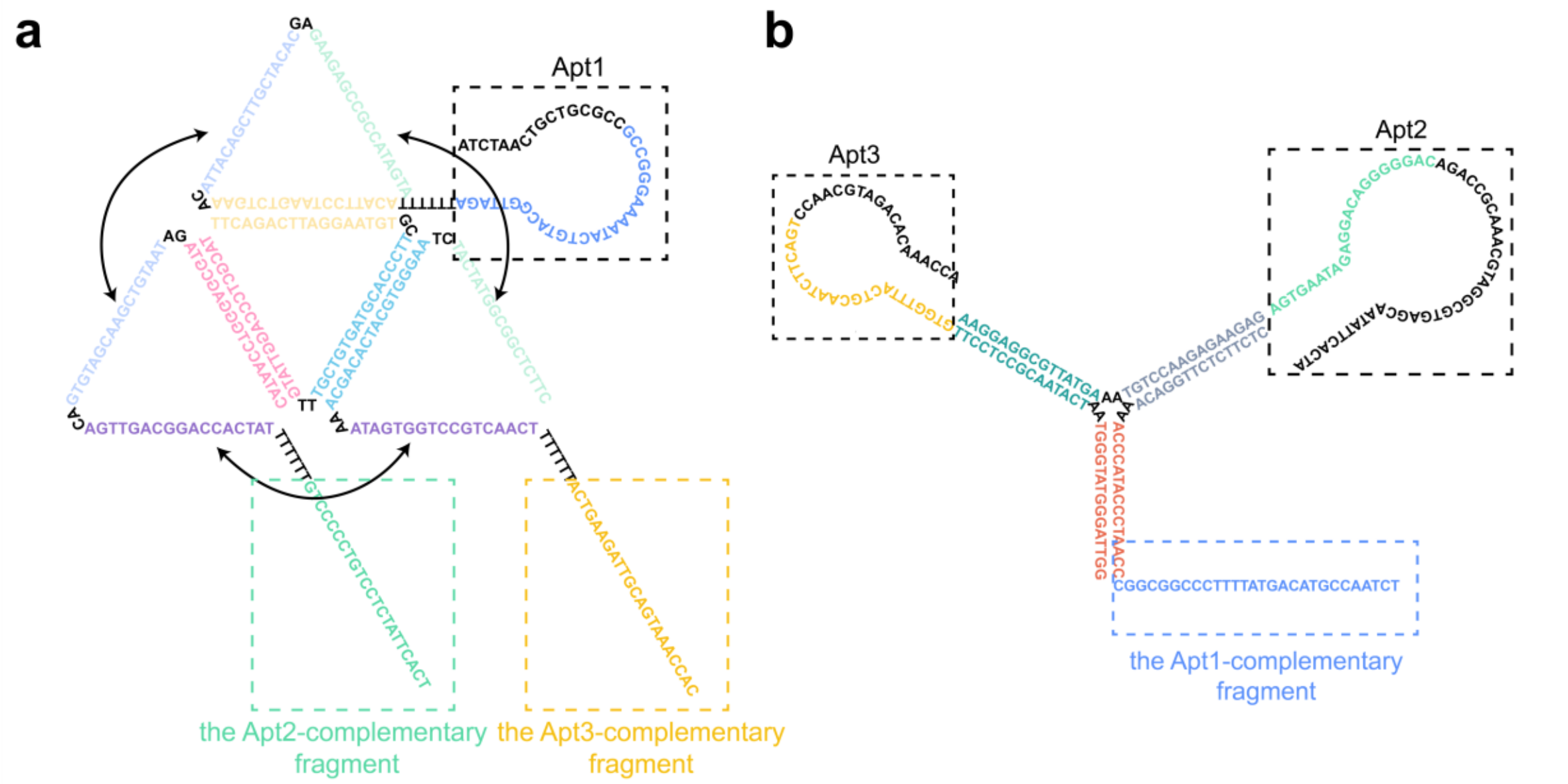

Supplementary Figure 1. Nucleotide sequences of the DND. **(a)** Sequence design of the tetrahedral framework nucleic acid (tFNA) and **(b)** the cholesterol-modified Y-shaped membrane-anchoring scaffold (Chol-MAS). Dashed boxes indicate the functional sequence modules, including aptamer1–aptamer3 and their corresponding aptamer binding complementary fragments.

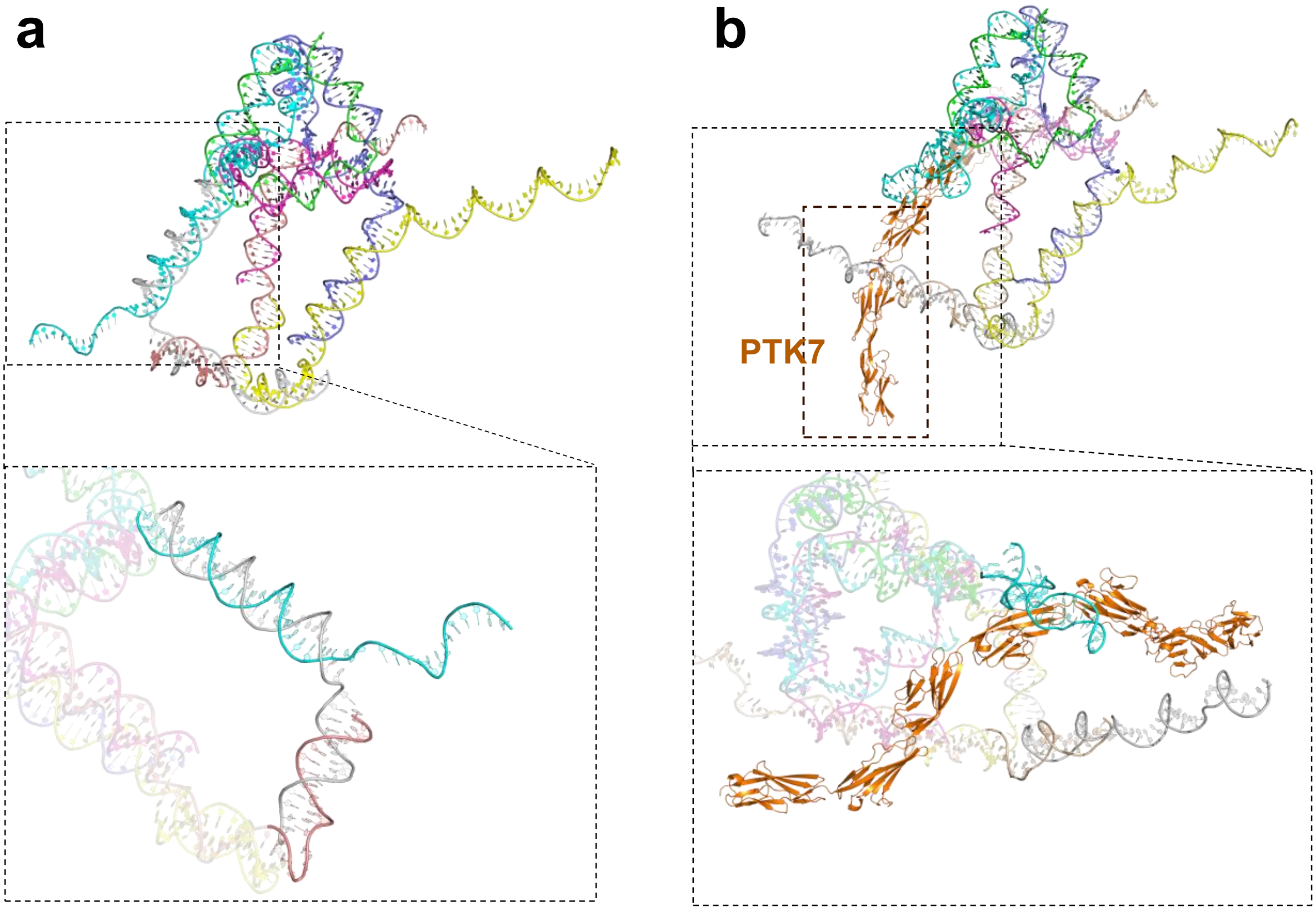

Supplementary Figure 2. Exploratory structural models of the DND before and after PTK7 docking.PTK7 was selected as the representative modeling target because it is the known molecular target of Sgc8c, whereas the molecular identities of the Sgc4f and TCO1 targets remain uncharacterized. **(a)** Theoretical prebinding model of the assembled DND containing the Sgc8c module. **(b)** Theoretical post-docking model of the DND–PTK7 complex. The docked PTK7 Ig3–Ig4 domain is labeled in the figure. Insets show enlarged local views of the Sgc8c-containing region before and after PTK7 docking.

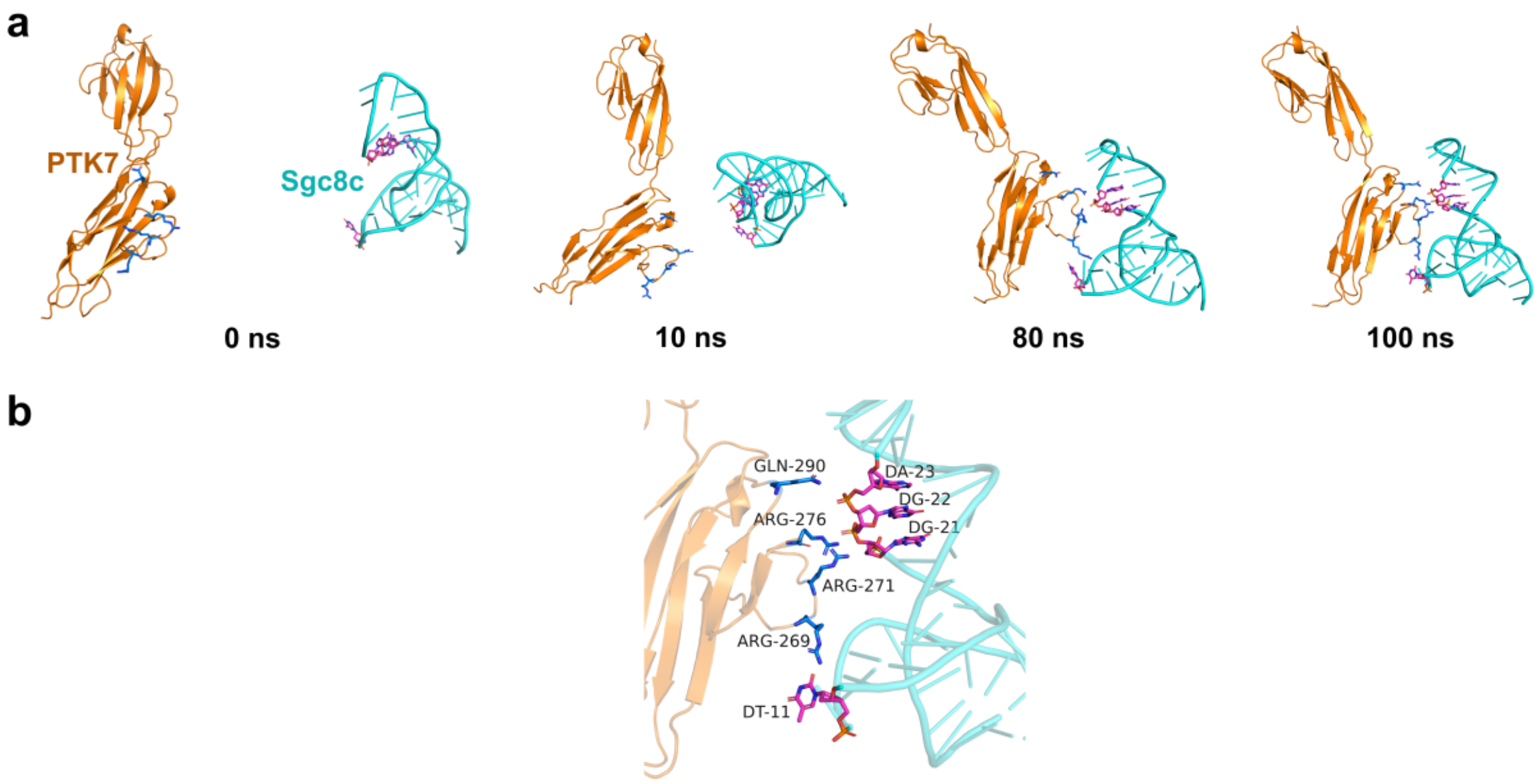

Supplementary Figure 3. Exploratory free-binding simulation of Sgc8c with PTK7. **(a)** Representative snapshots of Sgc8c and the PTK7 Ig3–Ig4 domain at 0, 10, 80, and 100 ns during an unrestrained simulation. **(b)** Enlarged view of the local contact region between Sgc8c and PTK7, with selected residues and nucleotides labeled.

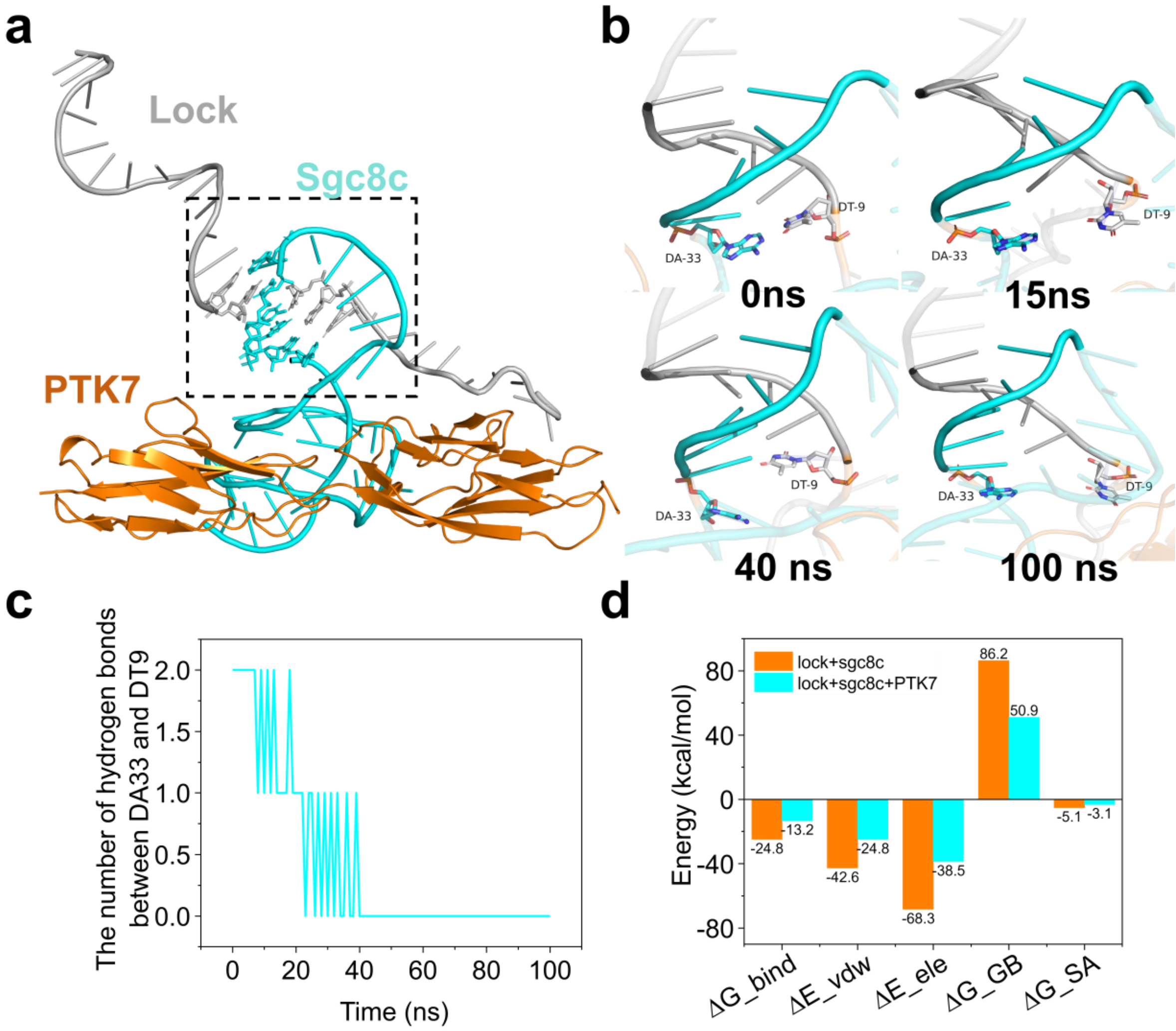

Supplementary Figure 4. Simplified sgc8c–lock–PTK7 model for evaluating local aptamer–lock coupling. **(a)** Structural model of the sgc8c–lock–PTK7 system. **(b)** Representative enlarged views of the local base-pairing region between sgc8c and the lock strand during the simulation. **(c)** Time-dependent change in the number of hydrogen bonds between DA33 and DT9 of the lock duplex during the 100 ns simulation. **(d)** MM/GBSA energy analysis of the lock–sgc8c interaction in the absence and presence of PTK7. Source data are provided as a Source Data file.

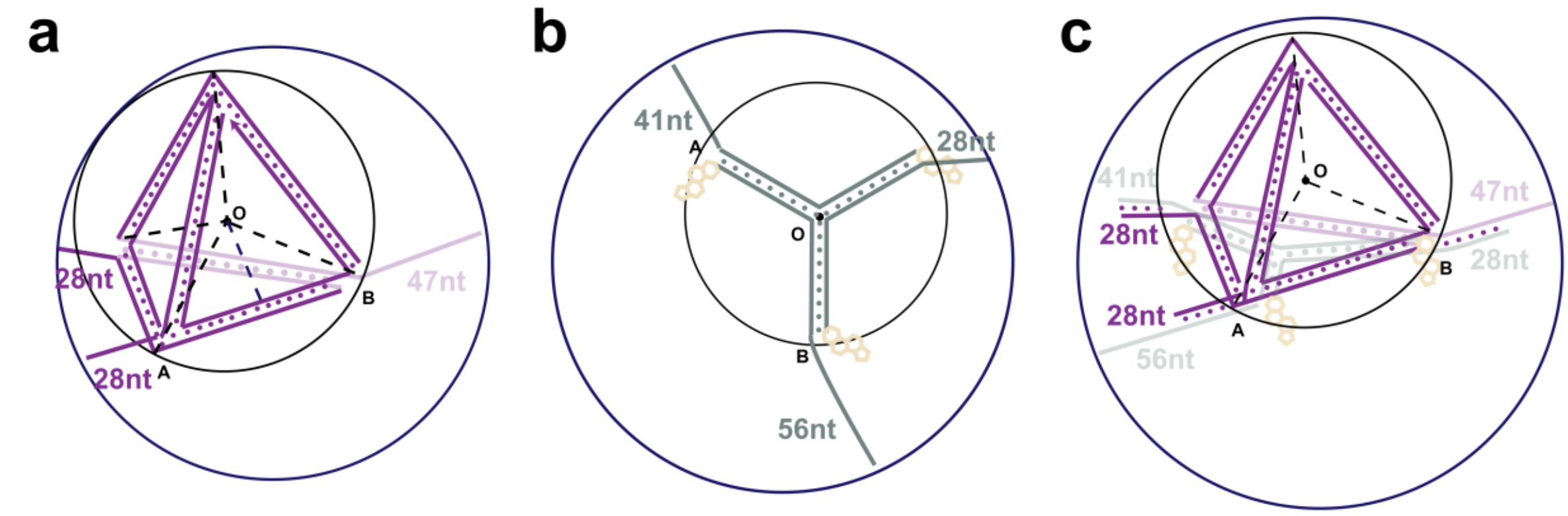

Supplementary Figure 5. Theoretical size estimation of tFNA, Chol-MAS, and DND. **(a)** tFNA. **(b)** Chol-MAS. **(c)** DND assembled from tFNA and Chol-MAS. The minimal dimensions were estimated from scaffold geometry, and the maximal spans were estimated by assuming fully extended single-stranded segments, using a contour length of 0.34 nm per nucleotide for conversion. The resulting theoretical size ranges are 7.1–31.3 nm for tFNA, 10.2–41.8 nm for Chol-MAS, and 10.2–43.9 nm for DND.

Discussion: The theoretical dimensions were estimated by converting nucleotide numbers to contour lengths and then applying simple geometric relations. *n* is the number of nucleotides. *L* is the contour length. *AB* is the tetrahedron edge length. *OA* is the arm length in Chol-MAS. *r* is the circumscribed radius. d_{min} and d_{max} denote the minimal diameter and maximal span, respectively.

$$l_0 = 0.34 \text{ nm} \cdot nt^{-1} \tag{1}$$

$$L = n \cdot l_0 \tag{2}$$

(a) tFNA

$$AB = 17 \text{ nt} \cdot l_0 = 5.78 \text{ nm} \tag{3}$$

$$r = \frac{\sqrt{6}}{4} AB \approx 3.54 \text{ nm} \tag{4}$$

$$d_{\mathrm{min,tFNA}} = 2r \approx 7.1\ \mathrm{nm} \tag{5}$$

$$d_{\mathrm{max,tFNA}} = (28 + 47) \cdot l_0 + AB = 31.28\ \mathrm{nm} \approx 31.3\ \mathrm{nm} \tag{6}$$

$$d_{\mathrm{tFNA}} = 7.1\text{–}31.3\ \mathrm{nm} \tag{7}$$

(b) Chol-MAS

$$OA = 15\ \mathrm{nt} \cdot l_0 = 5.10\ \mathrm{nm} \tag{8}$$

$$d_{\mathrm{min,Chol-MAS}} = 2OA = 10.2\ \mathrm{nm} \tag{9}$$

$$AB = \sqrt{3OA} = 8.83\ \mathrm{nm} \tag{10}$$

$$d_{\mathrm{max,Chol-MAS}} = AB\ +\ (56\ +\ 41) \cdot l_0 \approx 41.8\ \mathrm{nm} \tag{11}$$

$$d_{\mathrm{Chol-MAS}} = 10.2\text{–}41.8\ \mathrm{nm} \tag{12}$$

(c) DND

$$d_{\mathrm{min,DND}} = 2OA = 10.2\ \mathrm{nm} \tag{13}$$

$$d_{\mathrm{max,DND}} = \sqrt{3} \cdot OA\ +\ (47\ +\ 56) \cdot l_0 \approx 43.9\ \mathrm{nm} \tag{14}$$

$$d_{\mathrm{DND}} = 10.2\text{–}43.9\ \mathrm{nm} \tag{15}$$

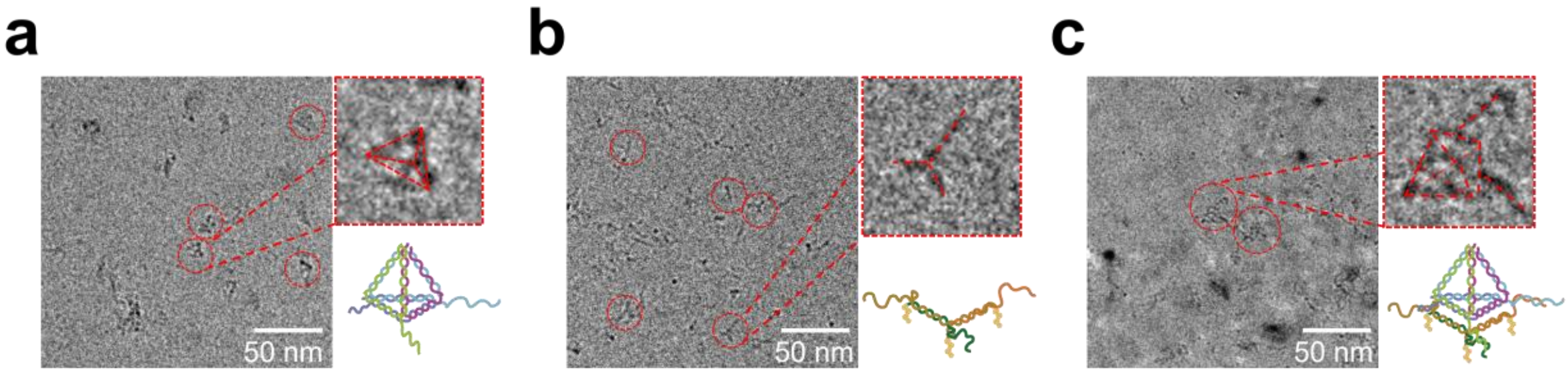

Supplementary Figure 6. Representative cryo-electron microscopy (Cryo-EM) micrographs of DND and its structural modules. **(a)** tFNA, **(b)** Chol-MAS, and **(c)** DND in vitreous ice. Insets show enlarged views of representative particles. The experiment was repeated independently for 3 times with similar results. Scale bars, 50 nm. Source data are provided as a Source Data file.

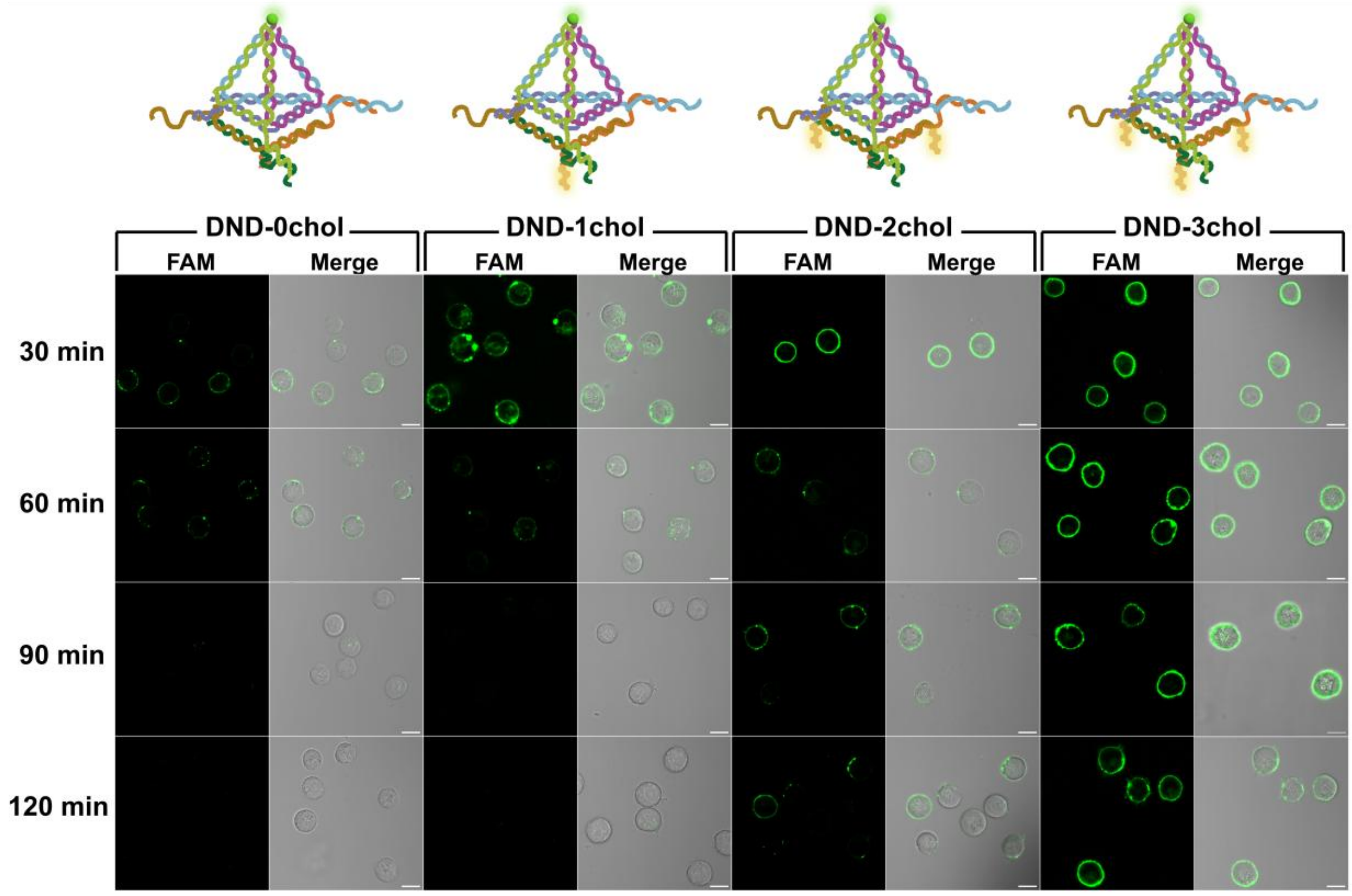

Supplementary Figure 7. Serum stability of membrane anchoring for different DND variants. Ramos cells were incubated with FAM-labeled DND-0chol, DND-1chol, DND-2chol, or DND-3chol in medium containing 10% fetal bovine serum (FBS), and imaged at 30–120 min. Representative CLSM images are shown from three independent experiments with similar results. Scale bars, 10 μm.

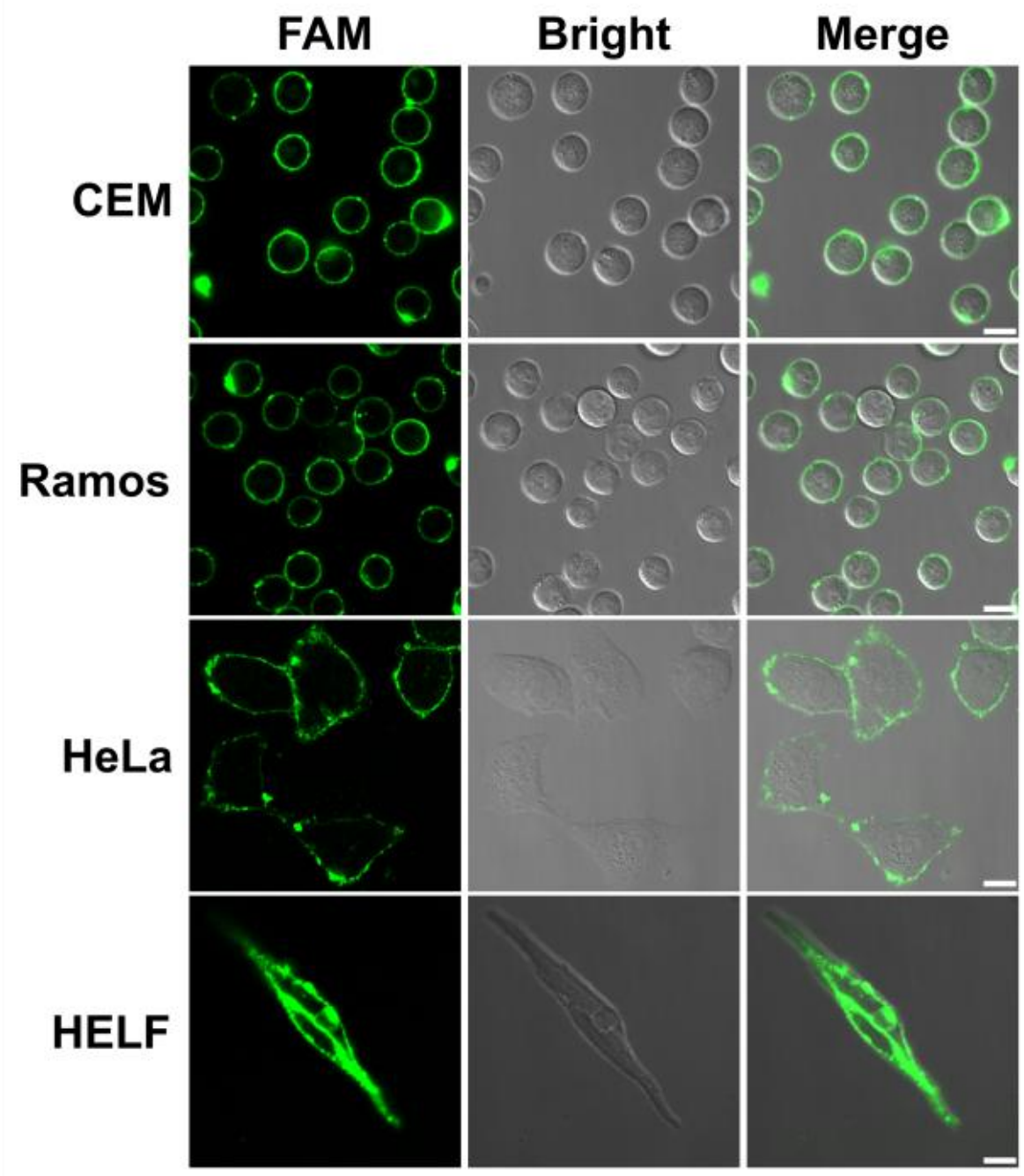

Supplementary Figure 8. Membrane anchoring of DND in different cell lines.CLSM images of cells incubated with FAM-labeled DND (20 min, 37 °C). Representative CLSM images are shown from three independent experiments with similar results. Scale bars, 10 μm.

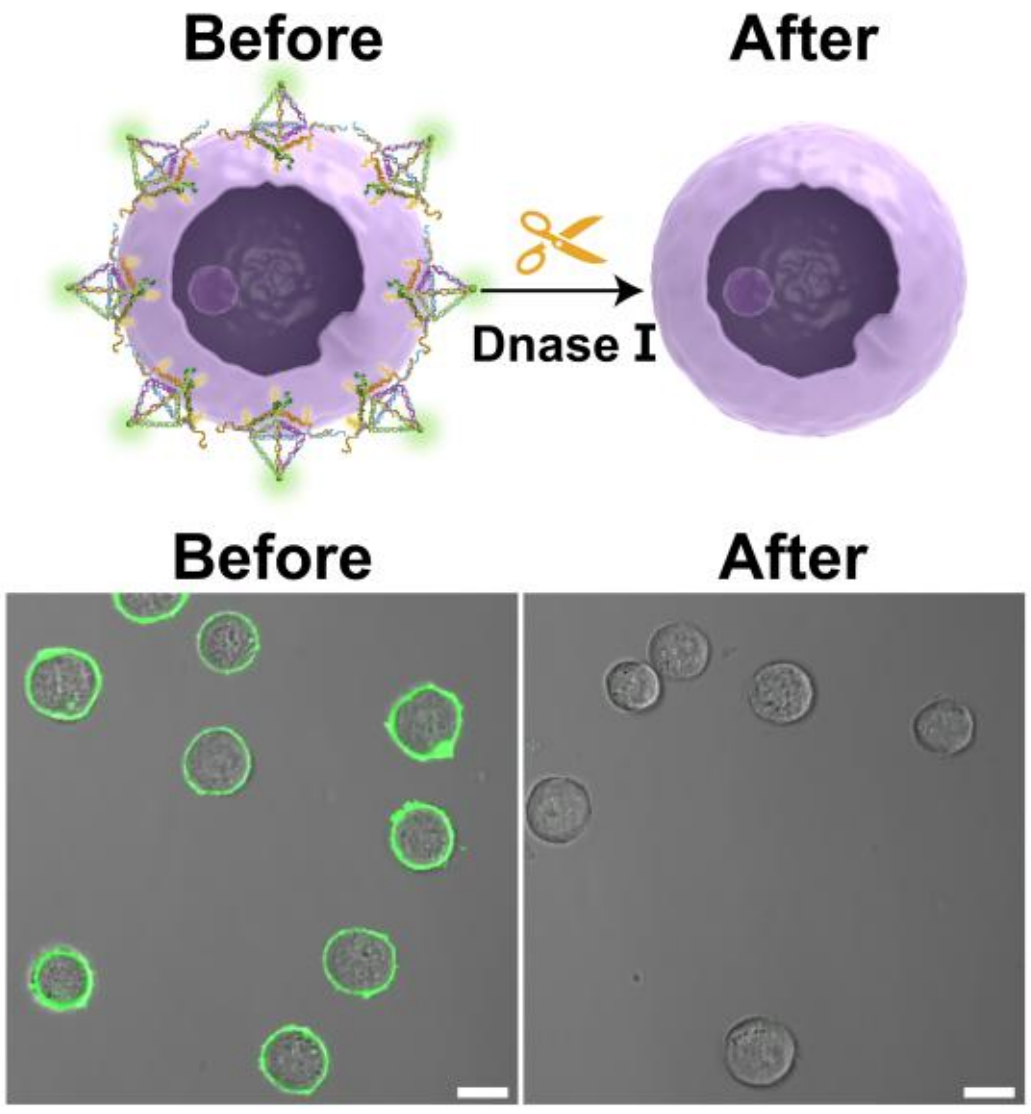

Supplementary Figure 9. DNase I validation of surface-localized DND. Ramos cells incubated with 250 nM FAM-labeled DND were imaged by CLSM before and after DNase I treatment (1 U/µL) at 37 °C for 10 min. Representative merged bright-field and fluorescence images are shown. Representative CLSM images are shown from three independent experiments with similar results. Scale bars, 10 µm.

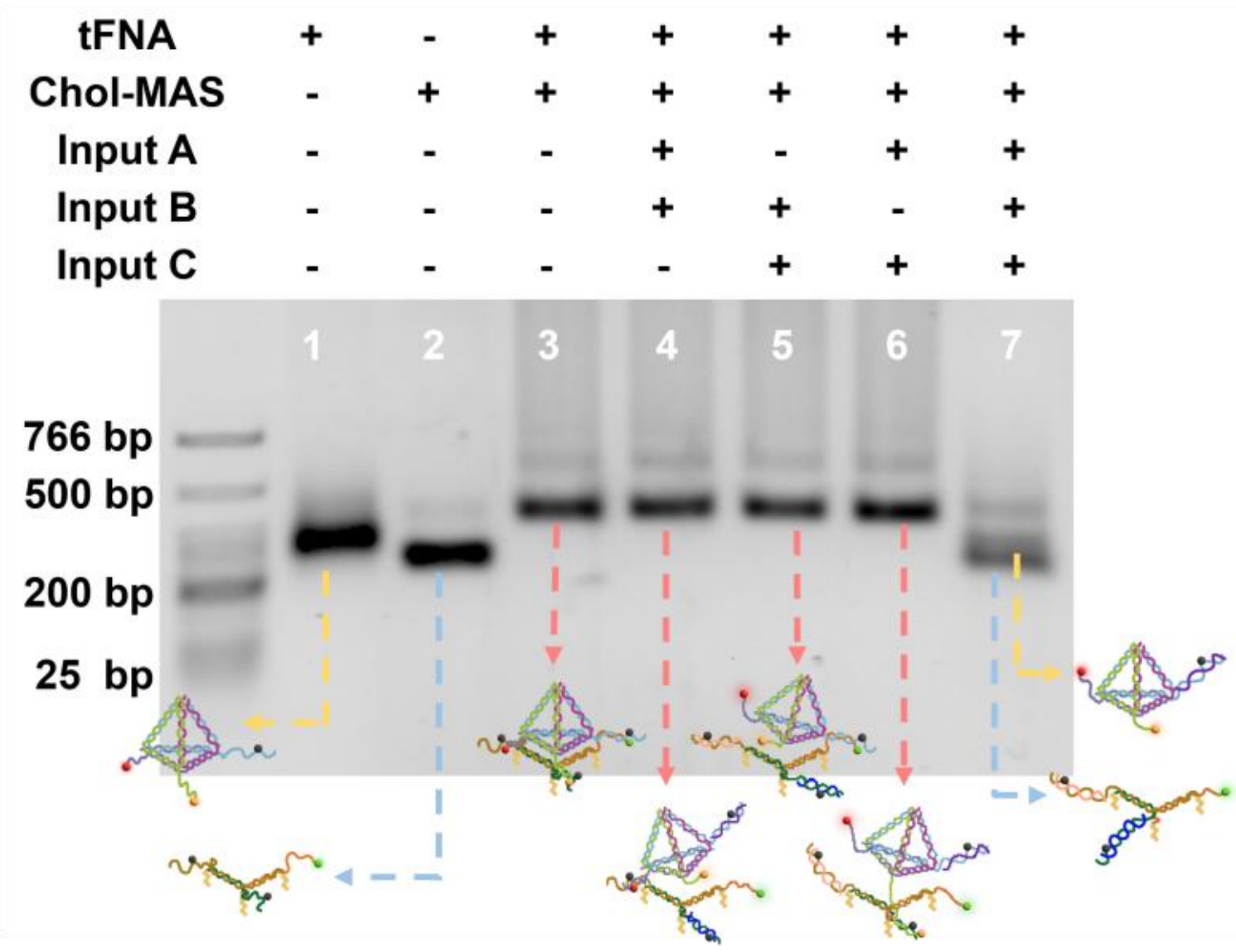

Supplementary Figure 10. Agarose gel analysis of target-responsive DND disassembly. Lane 1, tFNA. Lane 2, Chol-MAS. Lane 3, intact DND. Lanes 4–7, DND incubated with the indicated complementary inputs (C-Sgc8c, C-Sgc4f and/or C-TCO1), showing input-dependent disassembly and release of the tFNA. Representative gel images are shown from three independent experiments with similar results. Source data are provided as a Source Data file.

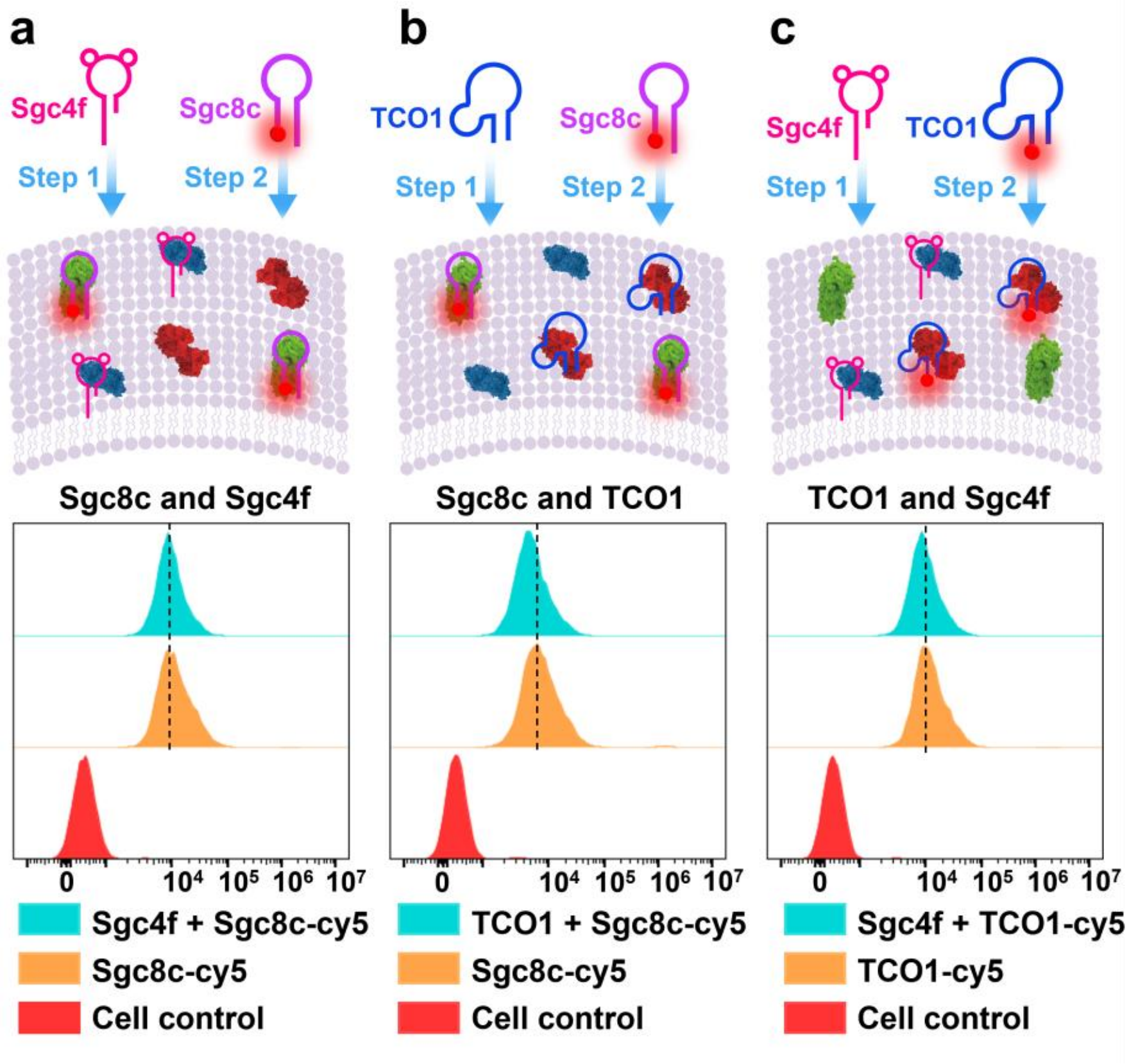

Supplementary Figure 11. Aptamer binding competition assay in CEM cells. In each experiment, cells were first incubated with an unlabeled aptamer (step 1), followed by a Cy5-labeled aptamer that recognized a different membrane protein (step 2). Flow cytometry histograms showed that the Cy5 fluorescence intensity was not reduced when the second aptamer was added after pre-incubation with another aptamer, that is **(a)** Sgc8c after Sgc4f pre-binding, **(b)** Sgc8c after TCO1 pre-binding, and **(c)** TCO1 after Sgc4f pre-binding. The experiment was repeated independently for 3 times with similar results.

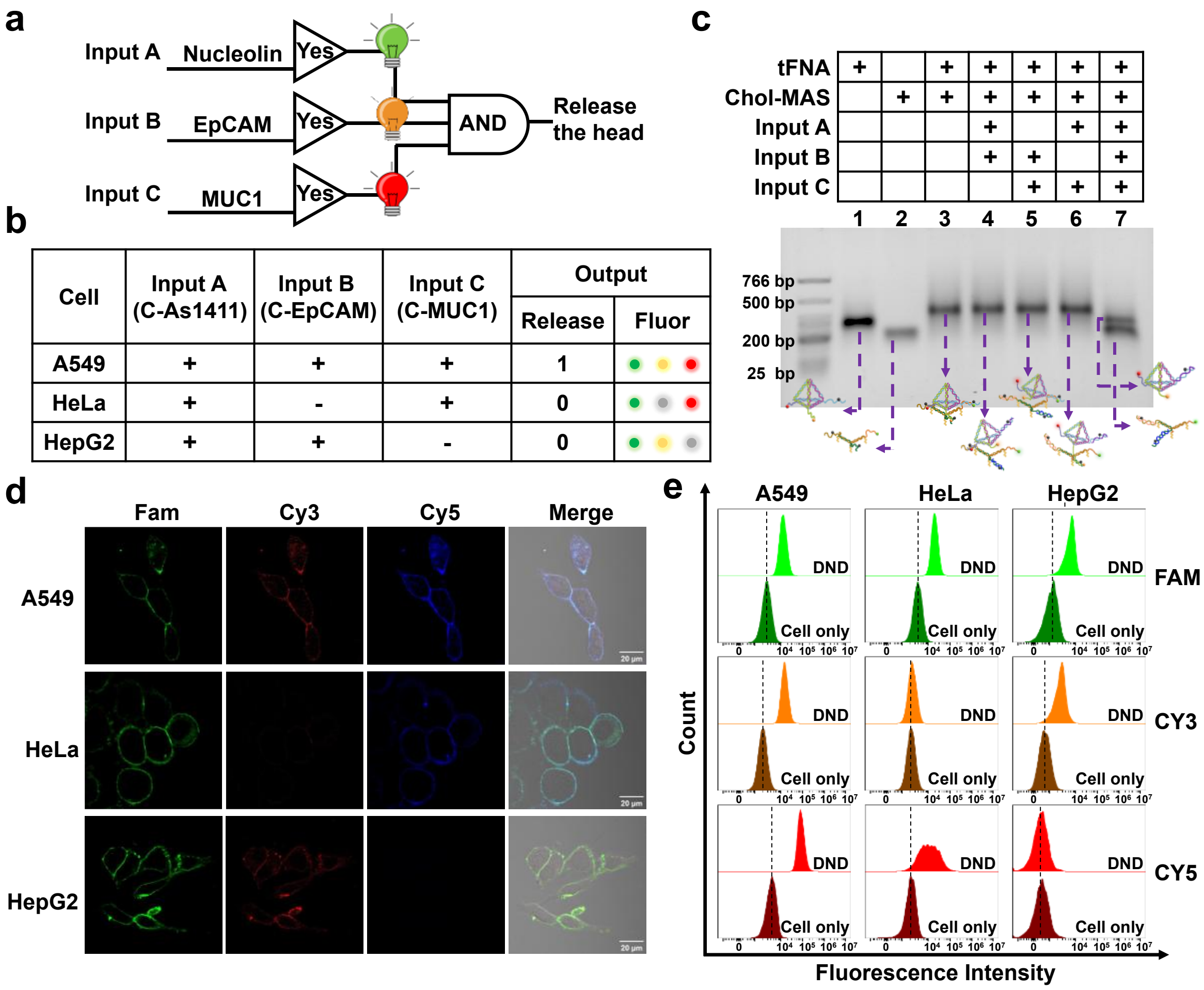

Supplementary Figure 12. Logic operation of the second DND system in different cell lines. **(a)** logic diagram and **(b)** Truth table of the three-input AND gate based on Nucleolin, EpCAM, and MUC1 aptamer recognition. C-AS1411 (FAM), C-EpCAM (Cy3), and C-MUC1 (Cy5). **(c)** 2% agarose gel electrophoresis analysis of tFNA, Chol-MAS, and their assemblies in the presence of different input combinations. Source data are provided as a Source Data file. **(d)** CLSM images and **(e)** flow cytometry histograms of A549, HeLa, and HepG2 cells after incubation with DND. Scale bars, 20 μm. The experiment was repeated independently for 3 times with similar results.

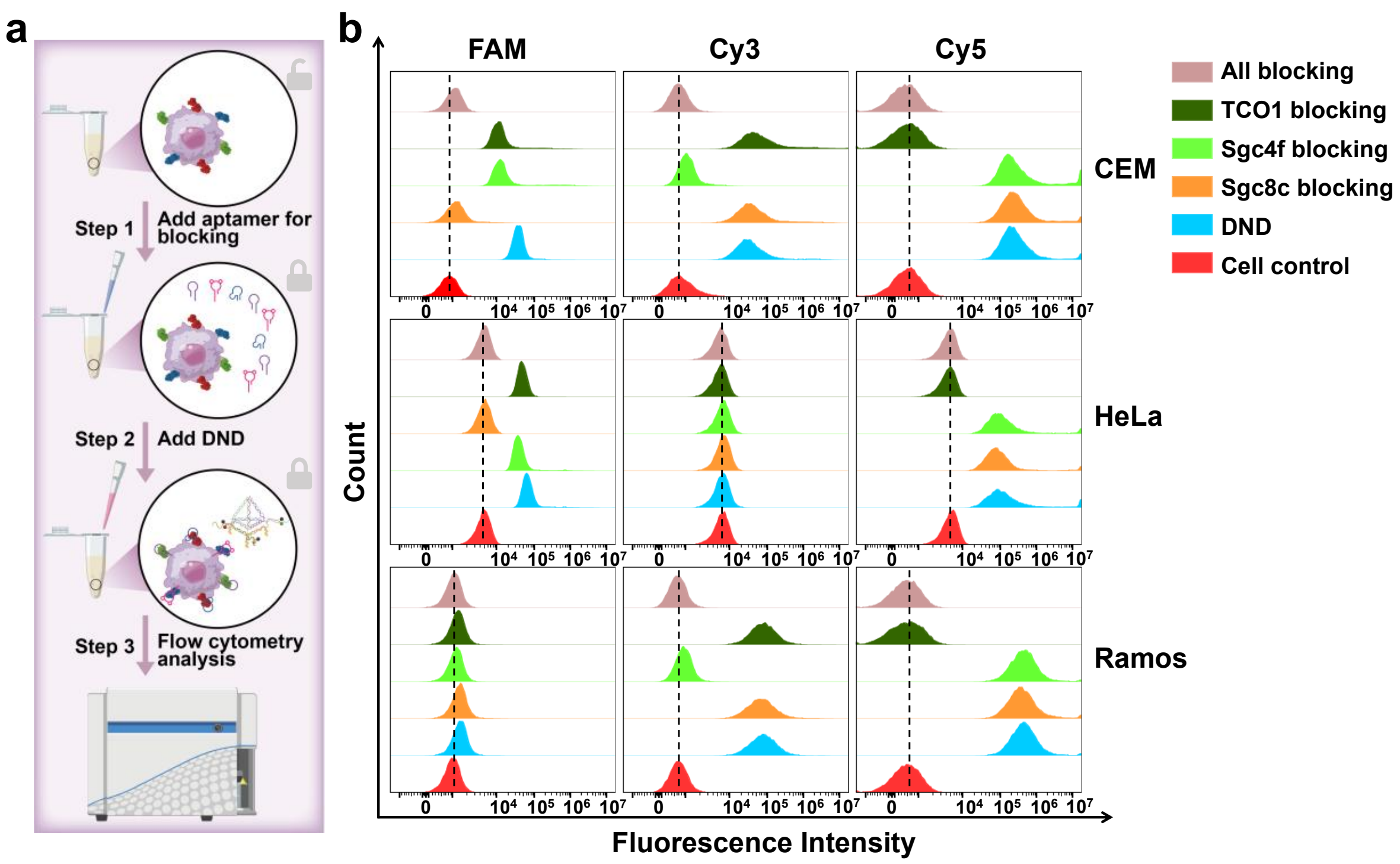

Supplementary Figure 13. Competitive binding assay for DND recognition. **(a)** Schematic of the protocol in which cells are pre-incubated with excess free aptamer for target blocking, followed by incubation with fluorophore-labeled DND and flow cytometry analysis. **(b)** Flow cytometry histograms of CEM, HeLa, and Ramos cells after pre-blocking with individual aptamers (Sgc4f, Sgc8c, or TCO1) or all three aptamers, and subsequent incubation with DND. The experiment was repeated independently for 3 times with similar results.

Discussion:

Supplementary Figure 13 shows blocking experiments designed to verify that DND recognizes cells through its three aptamer-functionalized arms. In this assay, cells were first preincubated with an excess of free aptamer to occupy the corresponding surface proteins, then incubated with fluorophore-labeled DND and analyzed by flow cytometry. In the histograms, the x axis represents fluorescence intensity in the FAM, Cy3, and Cy5 channels, and the rows correspond to CEM, HeLa, and Ramos

cells. Taking the top row of CEM cells as an example, the DND peaks are clearly shifted to the right relative to the cell-control peaks in all three channels, indicating that DND produces signals from all three arms on CEM cells. When cells are preblocked with a single type of free aptamer, the fluorescence signal in the corresponding channel is selectively reduced, whereas the other two channels remain essentially unchanged. For instance, preincubation of CEM cells with non-labeled free Sgc8c markedly suppresses the FAM signal to near the cell-control level, while the Cy3 and Cy5 channels still display strong signals comparable to those of unblocked DND. This response pattern indicates that each aptamer arm binds primarily to its corresponding target protein and that blocking one aptamer–protein interaction does not significantly affect recognition by the other two arms. When all three free aptamers are added simultaneously, the fluorescence distributions in all three channels of CEM cells are no longer markedly different from those of the cell-control group. These results show that DND association with the cell surface is governed mainly by specific aptamer–protein interactions rather than nonspecific adsorption. Overall, the data demonstrate that each arm of DND acts as an independent, highly selective recognition unit, and that the three arms work together to provide mutually orthogonal input channels for multi-receptor logic computation on the cell surface.

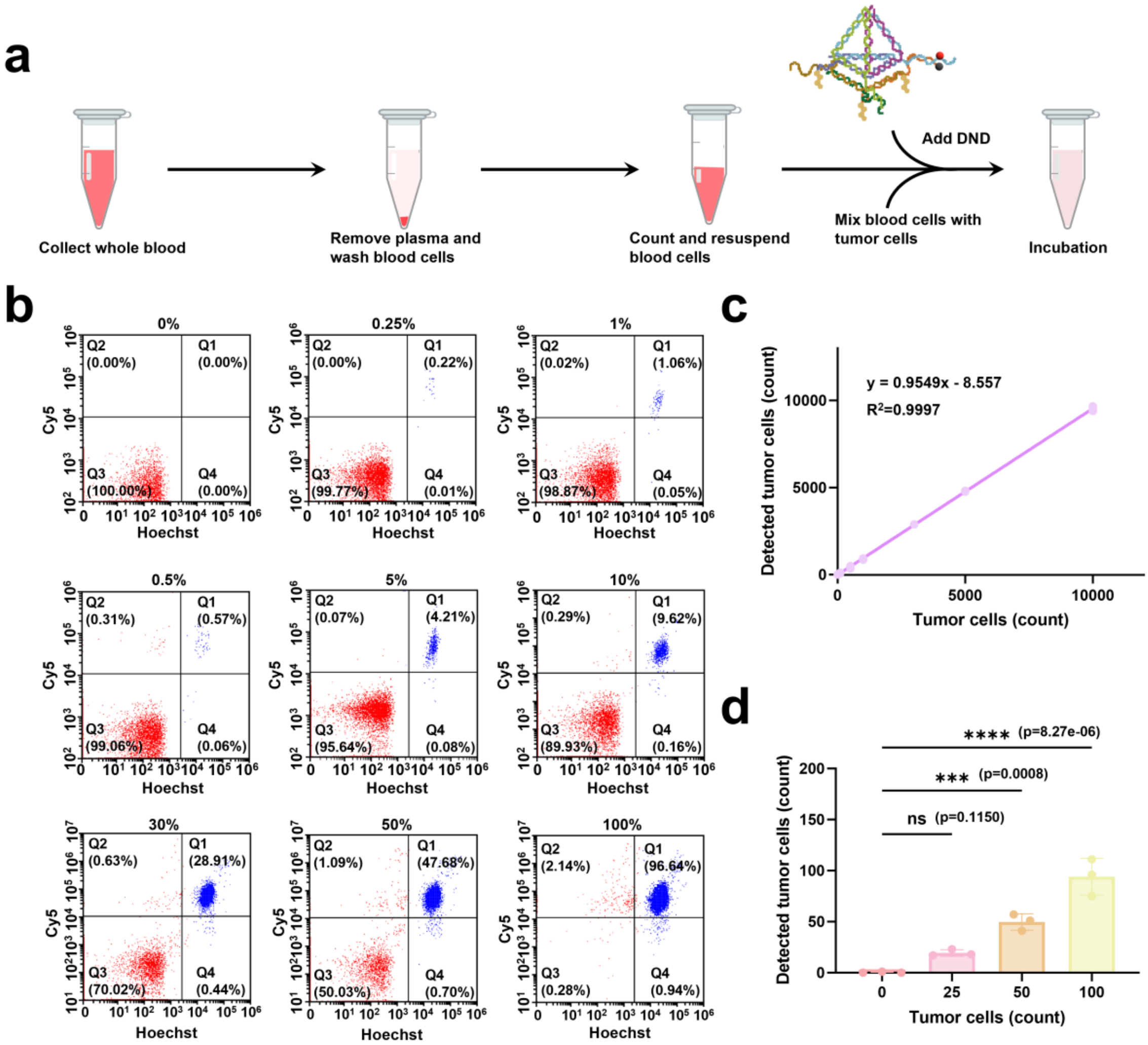

Supplementary Figure 14. Quantitative evaluation of CEM cell detection in a mouse blood-cell background. **(a)** Schematic illustration of the experimental workflow. Mouse whole blood was collected, followed by plasma removal, blood-cell washing, counting, and resuspension. Hoechst-prelabeled CEM cells were spiked into the washed mouse blood-cell suspension at predetermined levels, incubated with Cy5/BHQ2-labeled DND for 30 min, washed, and analyzed by flow cytometry. A total of 10,000 events were collected for each sample. **(b)** Representative flow cytometry dot plots of Hoechst-prelabeled CEM cells spiked into washed mouse blood-cell suspensions at different theoretical levels. Hoechst-positive cells were detected in the PB450 channel, and DND recognition was measured

in the Cy5 channel. Representative flow cytometry plots are shown from three independent experiments with similar results. **(c)** Linear fitting of theoretical tumor-cell numbers and detected tumor-cell numbers over the 0–10,000 cell range, with a fitting equation of $y = 0.9549x - 8.557$ and $R^2 = 0.9997$. **(d)** Detected tumor-cell numbers at different theoretical input levels over the 0–100 cell range. ($n = 3$ independently prepared blood-cell spiking samples). Bars represent the mean ± SD, and dots indicate individual measurements. Statistical significance was determined by one-way ANOVA followed by Dunnett's multiple comparisons test, with the 0-cell group as the control. ns, $P > 0.05$; ***$P < 0.001$; ****$P < 0.0001$. Source data are provided as a Source Data file.

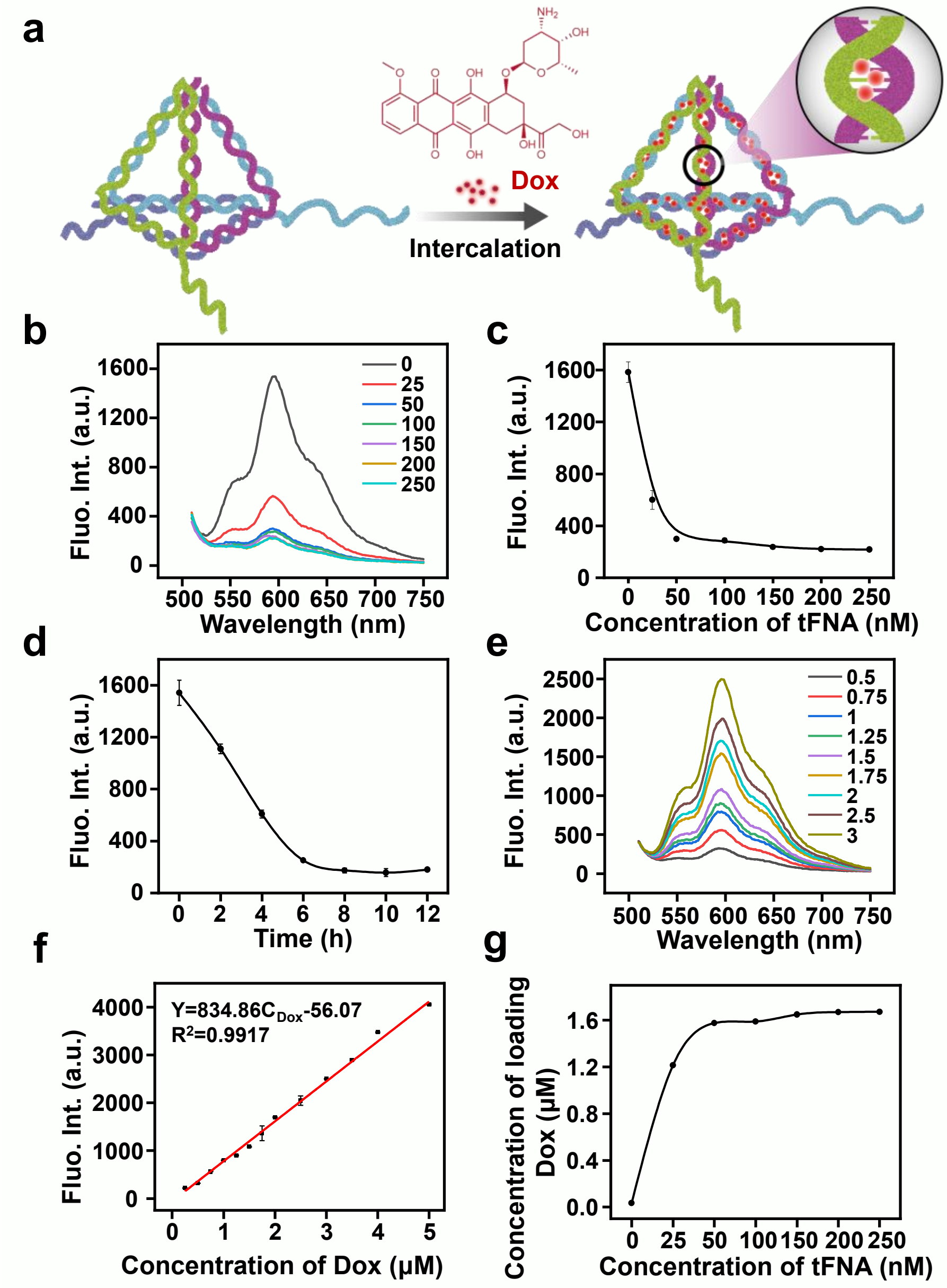

Supplementary Figure 15. Characterization of Dox loading into tFNA. Fluorescence signals arise from the intrinsic doxorubicin fluorescence. **(a)** Schematic illustration of Dox intercalation into tFNA. **(b)** Fluorescence spectra of free Dox in the presence of increasing tFNA concentrations and **(c)** the corresponding concentration-dependent quenching curve showing a progressive decrease in Dox fluorescence upon binding to tFNA. **(d)** Time-dependent decrease in Dox fluorescence indicating that intercalation reaches equilibrium within 8 h. **(e, f)** Fluorescence spectra of free Dox at defined

concentrations (e) and the corresponding calibration curve (f) used to quantify loaded Dox. **(g)** Loading capacity analysis showing that Dox incorporation increases with tFNA concentration and plateaus at about 1.5 μM, corresponding to a maximal loading ratio of ~30 Dox molecules per tFNA. Data are presented as mean ± SD (*n* = 3 independently prepared reaction samples). Source data are provided as a Source Data file.

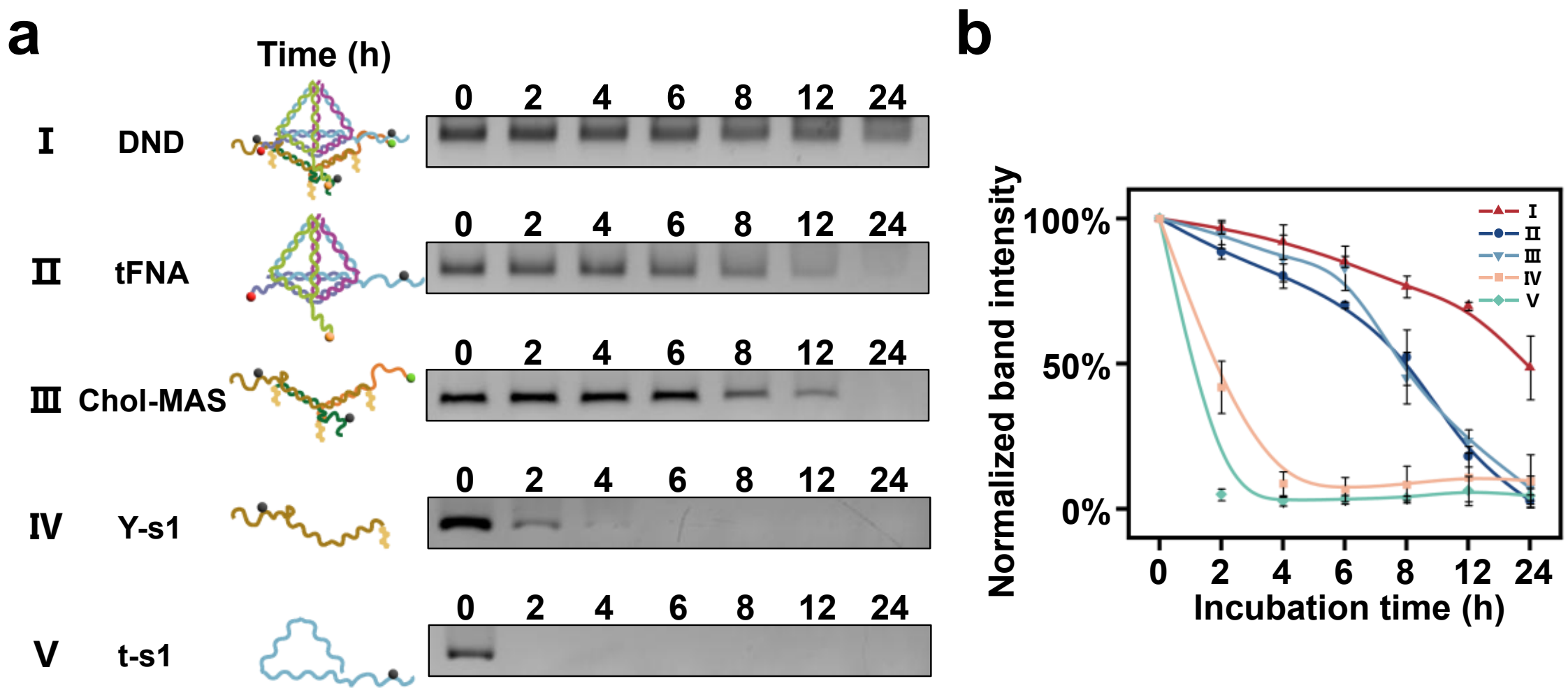

Supplementary Figure 16. Serum stability of DND and its components. **(a)** Agarose gel electrophoresis analysis monitoring the degradation of DND, tFNA, Chol-MAS, and single-stranded controls (Y-s1 and t-s1) after incubation in 10% FBS for 0–24 h. Representative gel images are shown from three independent experiments with similar results. **(b)** Quantification of the corresponding gel band intensities at the indicated time points. All gel experiments were performed and processed under the same conditions. Data are presented as mean ± SD (*n* = 3 independent gel experiments). Source data are provided as a Source Data file.

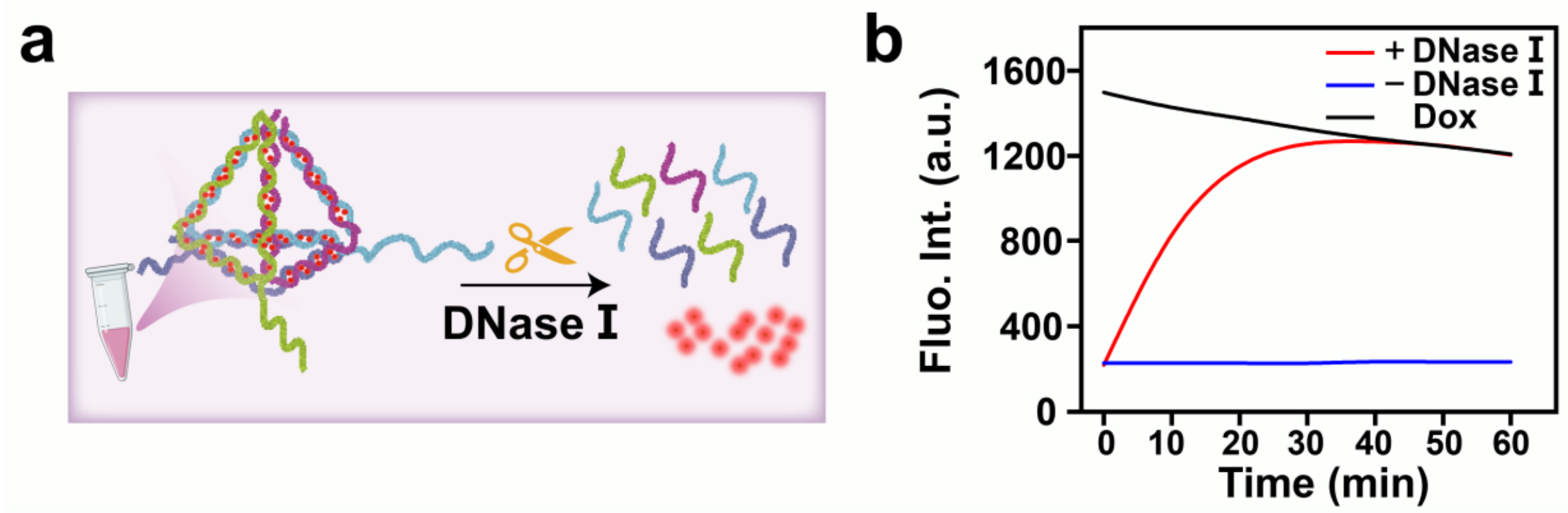

Supplementary Figure 17. DNase I-triggered drug release from tFNA@Dox. **(a)** Schematic illustration of the nuclease-mediated degradation of tFNA@Dox and subsequent Dox release. **(b)** Time dependent fluorescence recovery of tFNA@Dox incubated with DNase I (20 U/mL) or DNase-free buffer at 37 °C. Free Dox served as a control. Fluorescence signals arise from the intrinsic doxorubicin fluorescence. The experiment was repeated independently for 3 times with similar results. Source data are provided as a Source Data file.

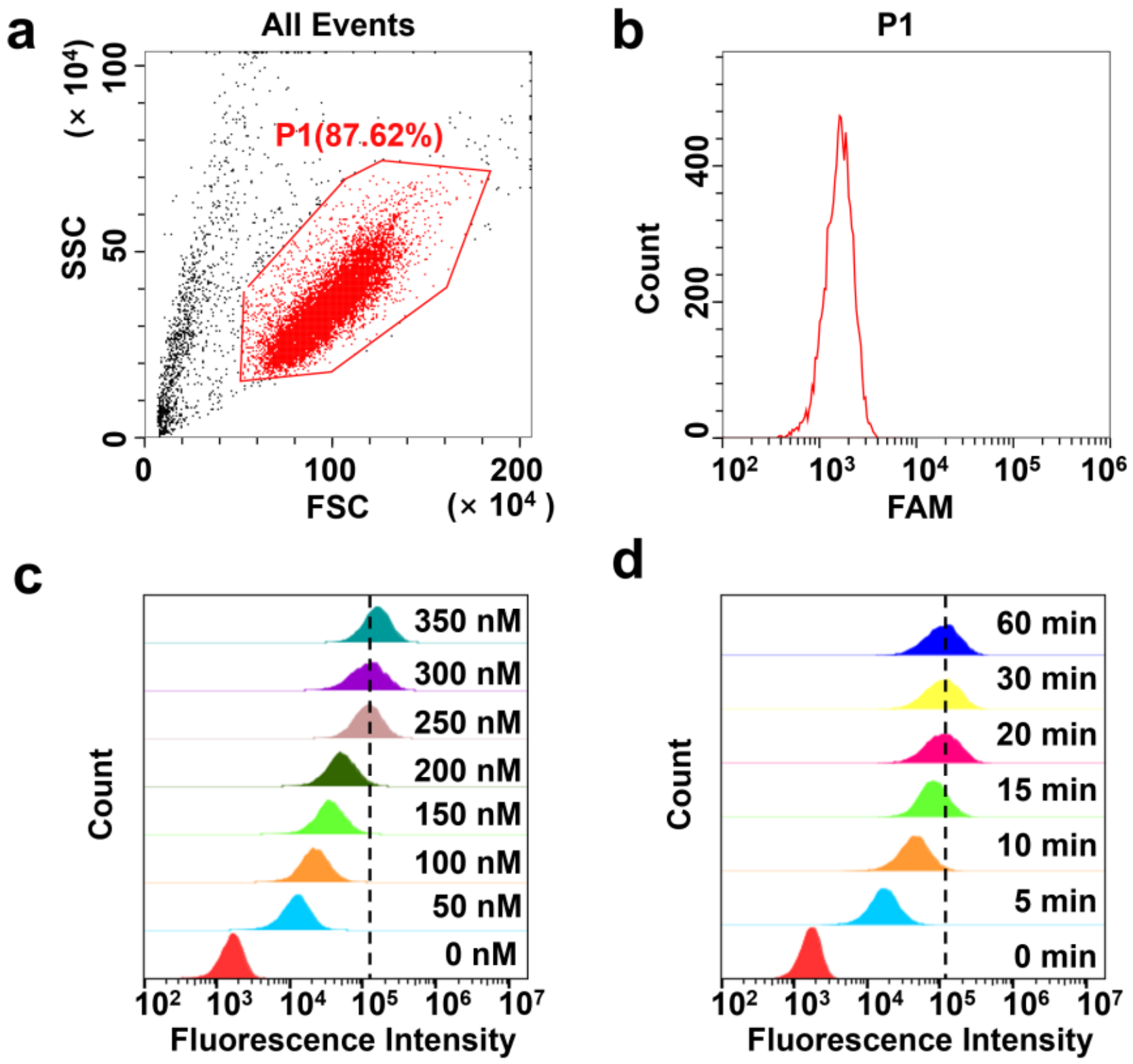

Supplementary Figure 18. Optimization of DND membrane anchoring. **(a)** Representative flow cytometry gating strategy used in this study. **(b)** Flow cytometry analysis of gated cells in the FAM channel. **(c)** Fluorescence histograms of Ramos cells incubated with FAM-labeled DND at concentrations ranging from 0 to 350 nM. **(d)** Fluorescence histograms of Ramos cells incubated with 250 nM FAM-labeled DND for varying times from 0 to 60 min. To avoid interference from tFNA internalization in CEM cells, all optimization experiments were performed in non-target Ramos cells. The experiment was repeated independently for 3 times with similar results.

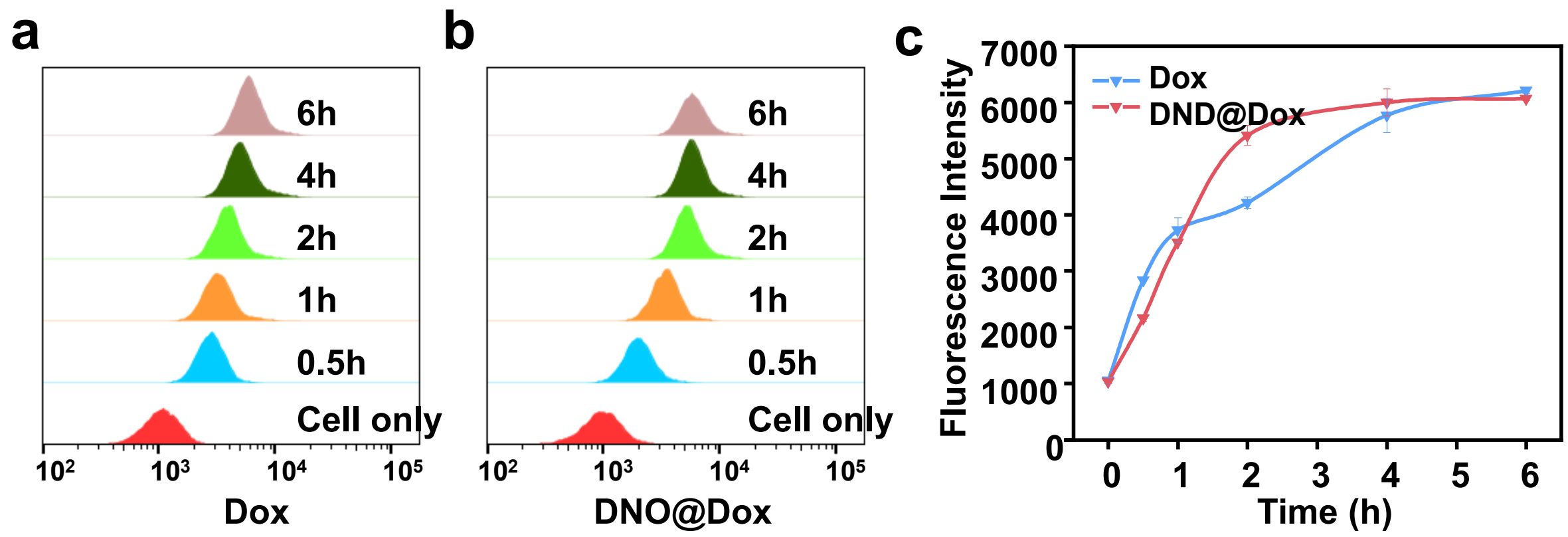

Supplementary Figure 19. Cellular uptake kinetics of free Dox and DND@Dox in CEM cells. **(a, b)** Flow cytometry histograms showing intracellular Dox fluorescence in CEM cells after incubation with free Dox (a) or DND@Dox (b) for 0.5–6 h. The experiment was repeated independently for 3 times with similar results. **(c)** Quantitative analysis of mean fluorescence intensity showing that the intracellular fluorescence signal increases over time and reaches a plateau at approximately 2 h. Fluorescence signals arise from the intrinsic doxorubicin fluorescence. Data are presented as mean ± SD ($n = 3$ from three independent experiments). Source data are provided as a Source Data file.

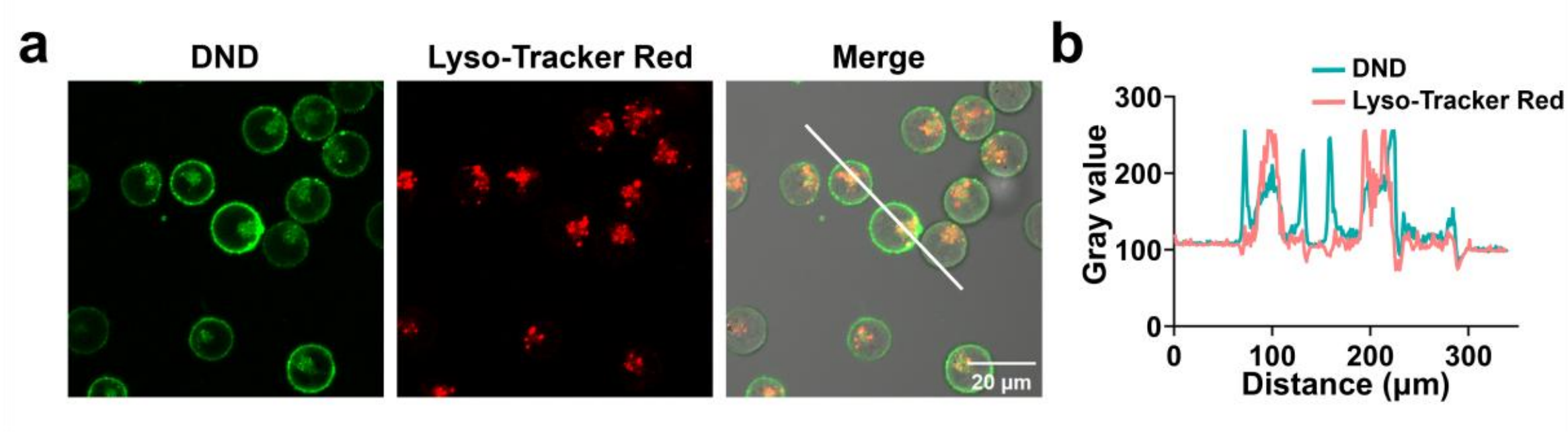

Supplementary Figure 20. Lysosomal trafficking analysis of DND. **(a)** CLSM images of CEM cells incubated with 250 nM FAM-labeled DND at 37 °C for 2 h, followed by Lyso-Tracker Red staining at 37 °C for 1 h. The merged image shows the spatial relationship between DND and lysosomes. Scale bar, 20 μm. **(b)** Line-scan fluorescence intensity profile along the white line indicated in (a), demonstrating colocalization between DND and Lyso-Tracker Red. The experiment was repeated independently for 3 times with similar results. Source data are provided as a Source Data file.

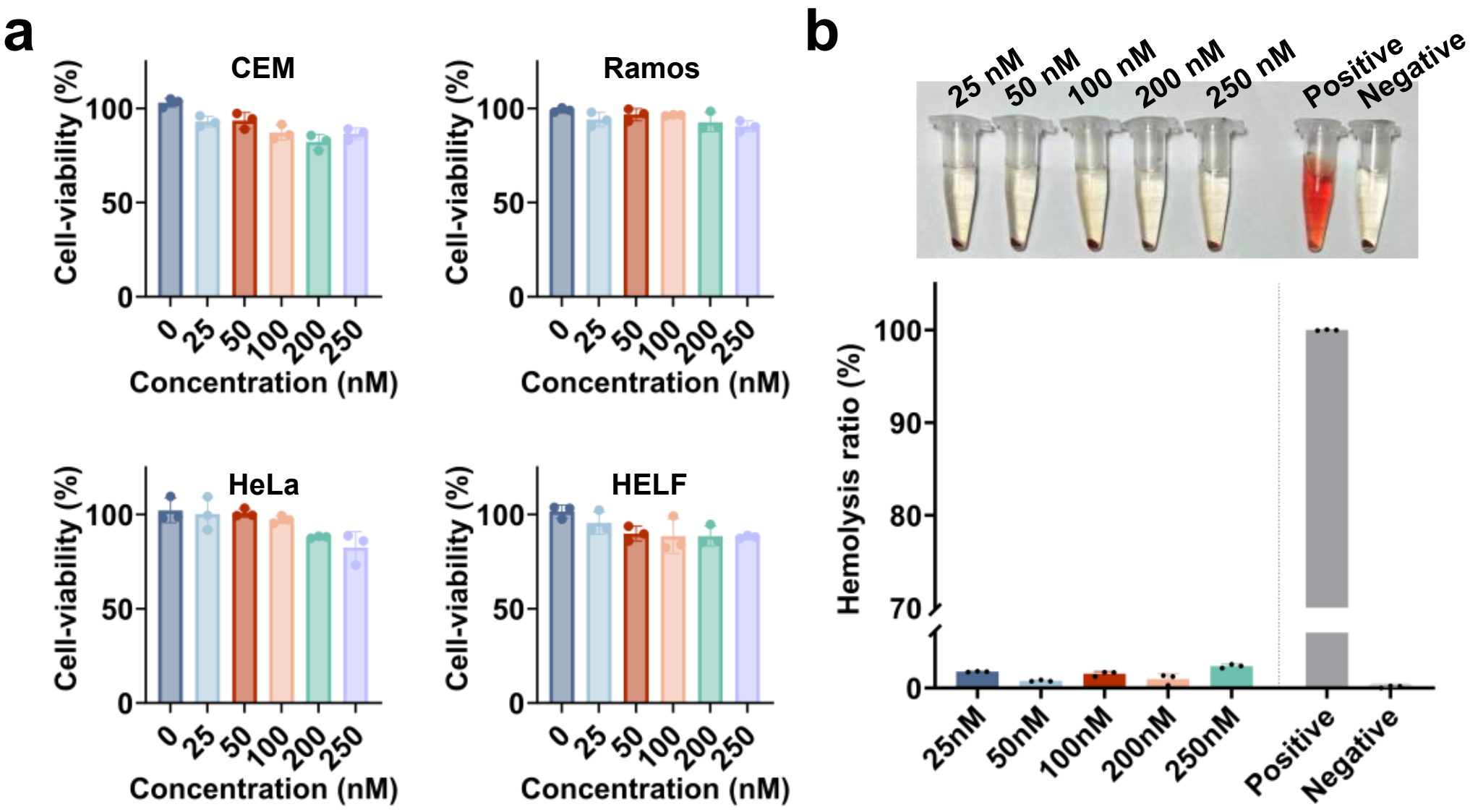

Supplementary Figure 21. In vitro biocompatibility of DND and DND@Dox. **(a)** Cell viability of CEM, Ramos, HeLa, and HELF cells after incubation with DND at the indicated concentrations from 0 to 250 nM for 48 h. **(b)** Hemolysis assay of DND@Dox at different concentrations from 25 to 250 nM in 2% red blood cell suspensions after incubation at 37 °C for 4 h. Representative photographs of supernatants after centrifugation are shown at the top, and the corresponding hemolysis ratios are shown at the bottom. H_2O and 0.9% NaCl were used as positive and negative controls, respectively. Data are presented as mean ± SD (*n* = 3 from three independent experiments). Source data are provided as a Source Data file.

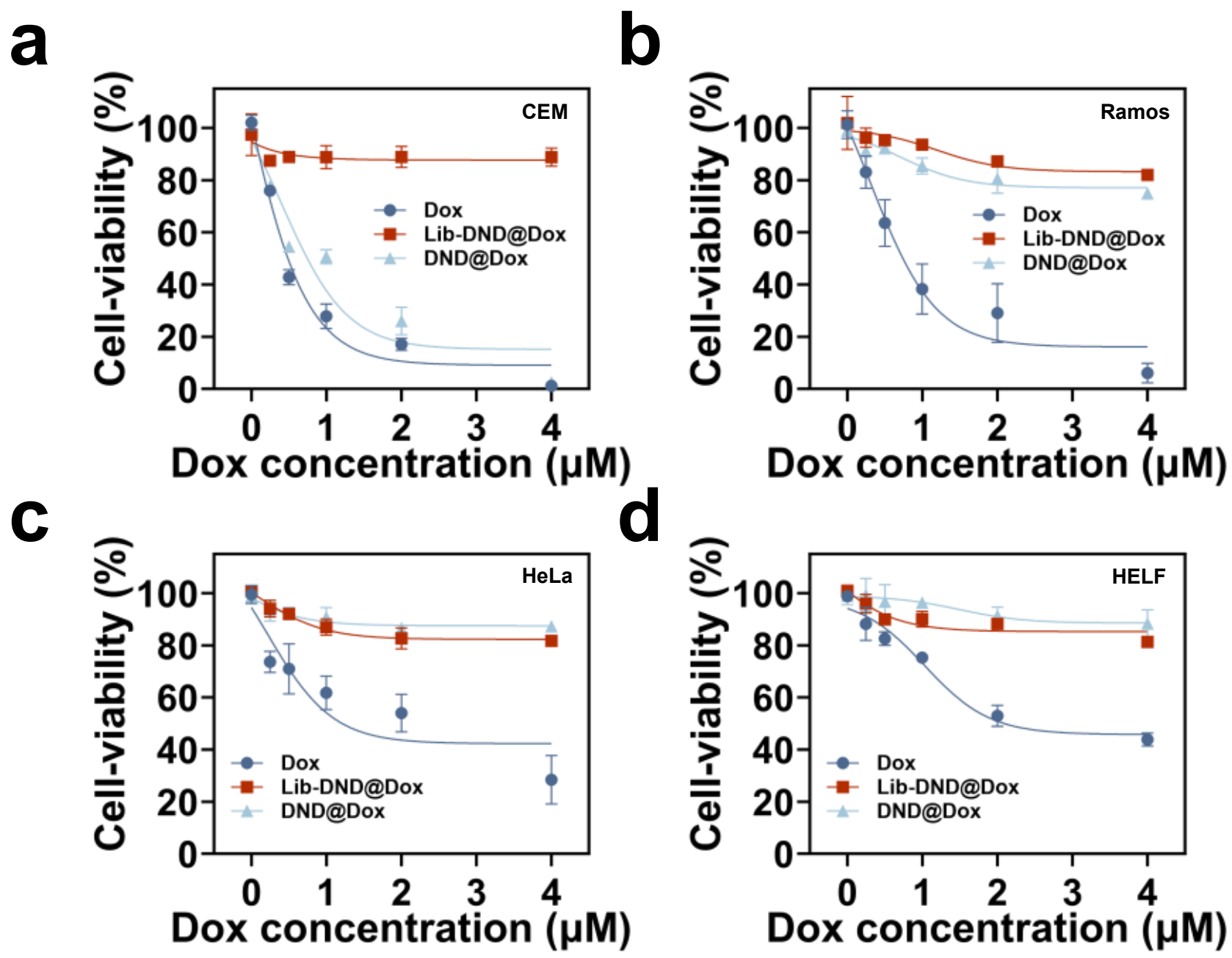

Supplementary Figure 22. Cytotoxicity of DND@Dox across different cell types. Cell viability of **(a)** CEM, **(b)** Ramos, **(c)** HeLa and **(d)** HELF cells measured after 48 h of treatment. Cells were treated with free doxorubicin (Dox), control Lib-DND@Dox (aptamer strands replaced by a random library), or DND@Dox over a Dox concentration range of 0–4 μM. Data are presented as mean ± SD (n = 3 from three independent experiments). Source data are provided as a Source Data file.

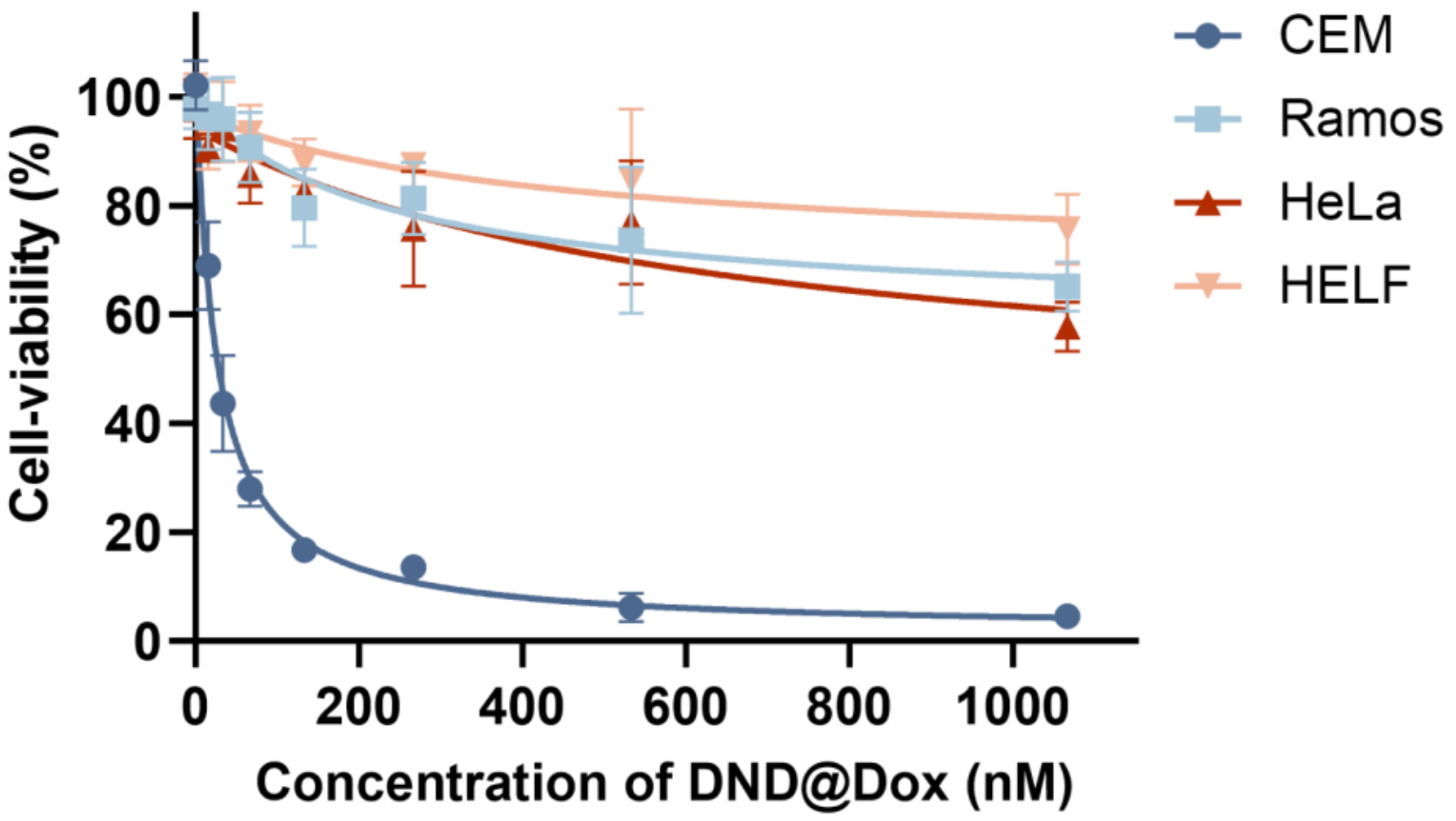

Supplementary Figure 23. Cell viability over an extended concentration range of DND@Dox. Cell viability of CEM, Ramos, HeLa, and HELF cells was measured after 48 h of treatment with DND@Dox at the indicated concentrations. Data are presented as mean ± SD (*n* = 3 from three independent experiments). Source data are provided as a Source Data file.

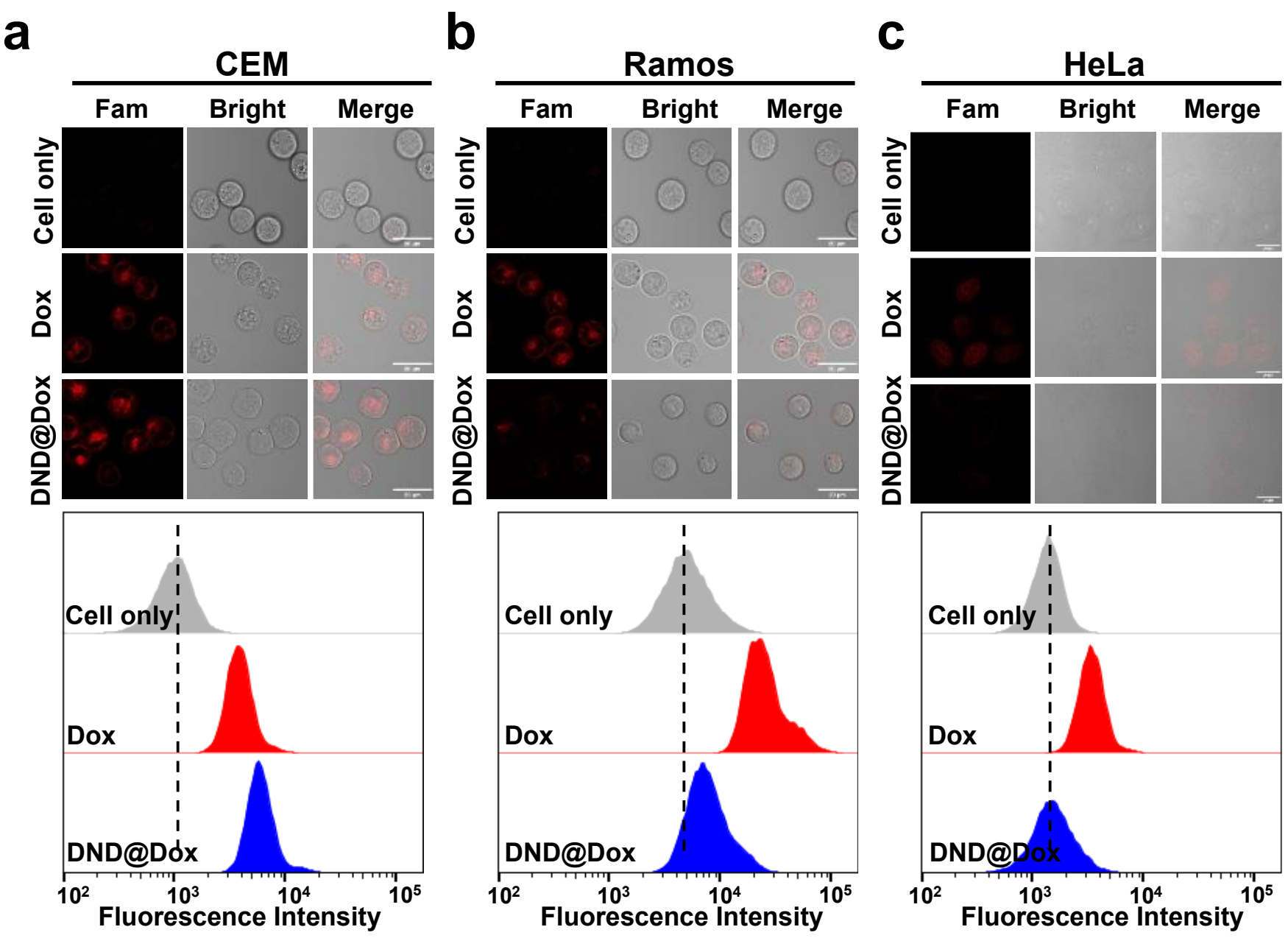

Supplementary Figure 24. Cell-selective delivery of DND@Dox. CLSM images and flow cytometry histograms of **(a)** CEM, **(b)** Ramos, and **(c)** HeLa cells after 2 h of incubation with free Dox or DND@Dox. Fluorescence signals arise from the intrinsic doxorubicin fluorescence. Scale bars, 20 μm. The experiment was repeated independently for 3 times with similar results.

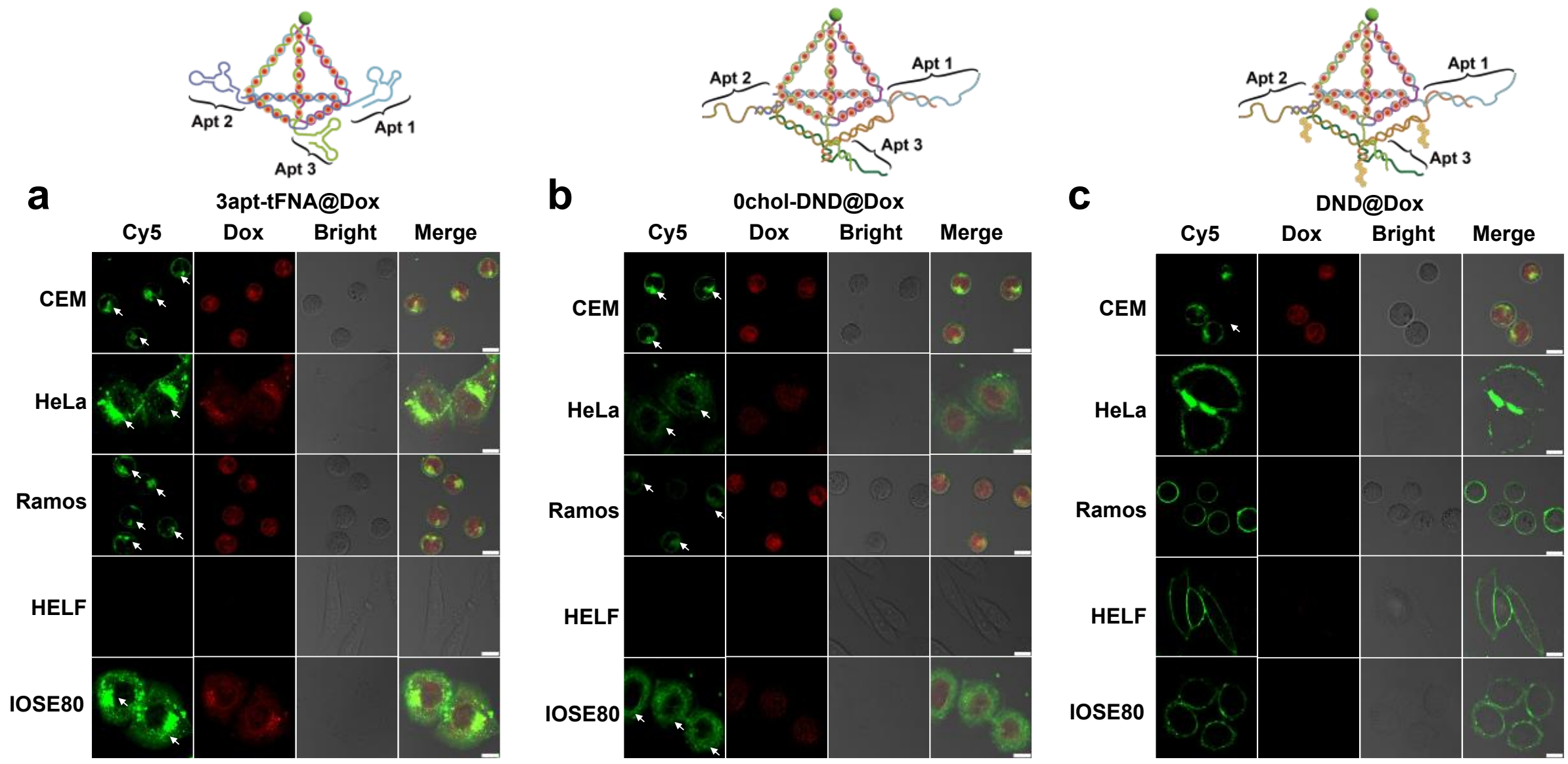

Supplementary Figure 25. Comparative study of the cellular localization and uptake of DND@Dox and related control constructs in different cell lines. **(a)** 3apt-tFNA@Dox, **(b)** 0chol-DND@Dox, and **(c)** DND@Dox. Confocal images were taken after 2 h incubation at 37 °C. Cy5 tracks the nanostructure, while intrinsic Dox fluorescence monitors drug delivery. Scale bars, 10 µm. The experiment was repeated independently for 3 times with similar results.

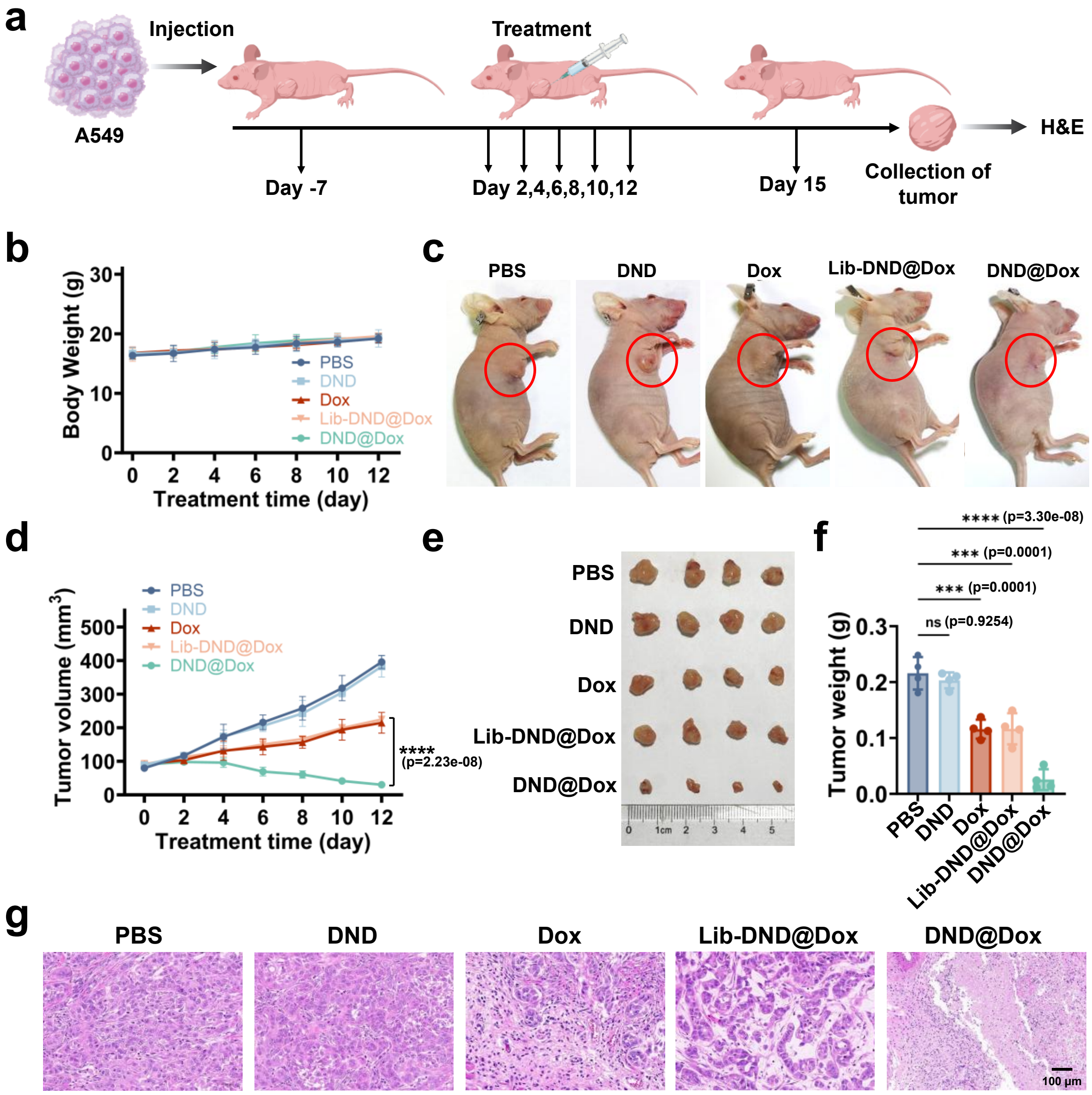

Supplementary Figure 26. Peritumoral therapy in A549 tumor-bearing mice. **(a)** Schematic of the peritumoral treatment schedule in A549 tumor-bearing mice. Tumors typically reached ~80–100 mm^3 about 1 week after inoculation, at which point mice were randomized into five groups: PBS, DND, free doxorubicin (Dox), DND@Dox, and Lib-DND@Dox. Treatments were administered by peritumoral injection every other day for a total of six doses. **(b)** Changes in average body weight of mice in each treatment group during the 12-day treatment period. **(c)** Representative photographs of tumor-bearing mice from each group at the study endpoint. **(d)** Tumor growth curves for the different treatment groups.

(e) Representative photographs of excised tumors on day 15. **(f)** Weights of the harvested tumors. Data are presented as mean ± SD (n = 4 biologically independent mice per group). Statistical significance was evaluated using one-way ANOVA followed by Tukey's multiple comparisons test; ***$P < 0.001$, ****$P < 0.0001$, ns $P > 0.05$. **(g)** Representative H&E-stained images are shown from four biologically independent mice per group. Scale bar, 100 μm. Source data are provided as a Source Data file.

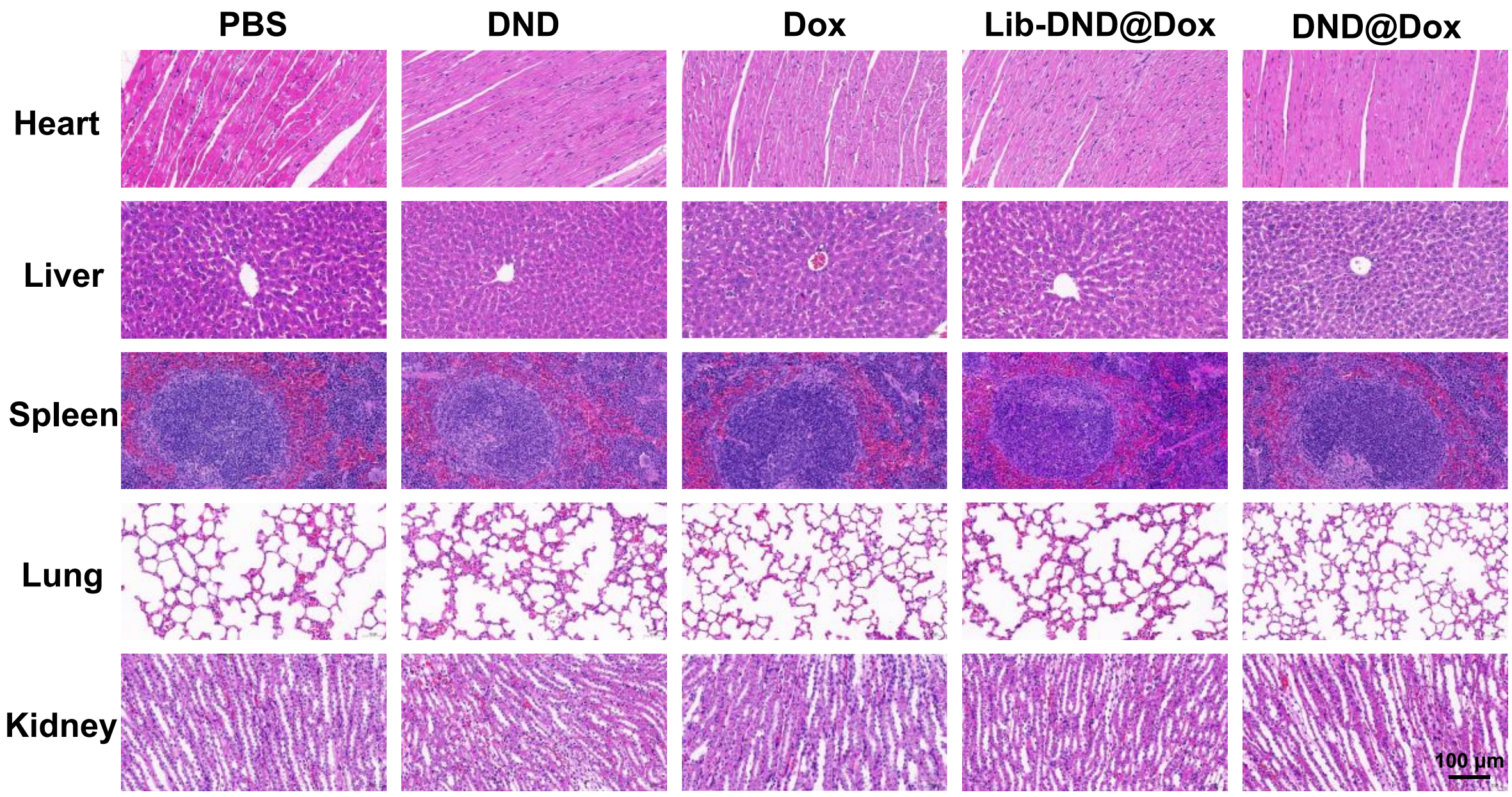

Supplementary Figure 27. H&E staining of major organs after peritumoral treatment. Representative heart, liver, spleen, lung, and kidney sections from each group. Representative images are shown from four biologically independent mice per group. Scale bar, 100 μm. Source data are provided as a Source Data file.

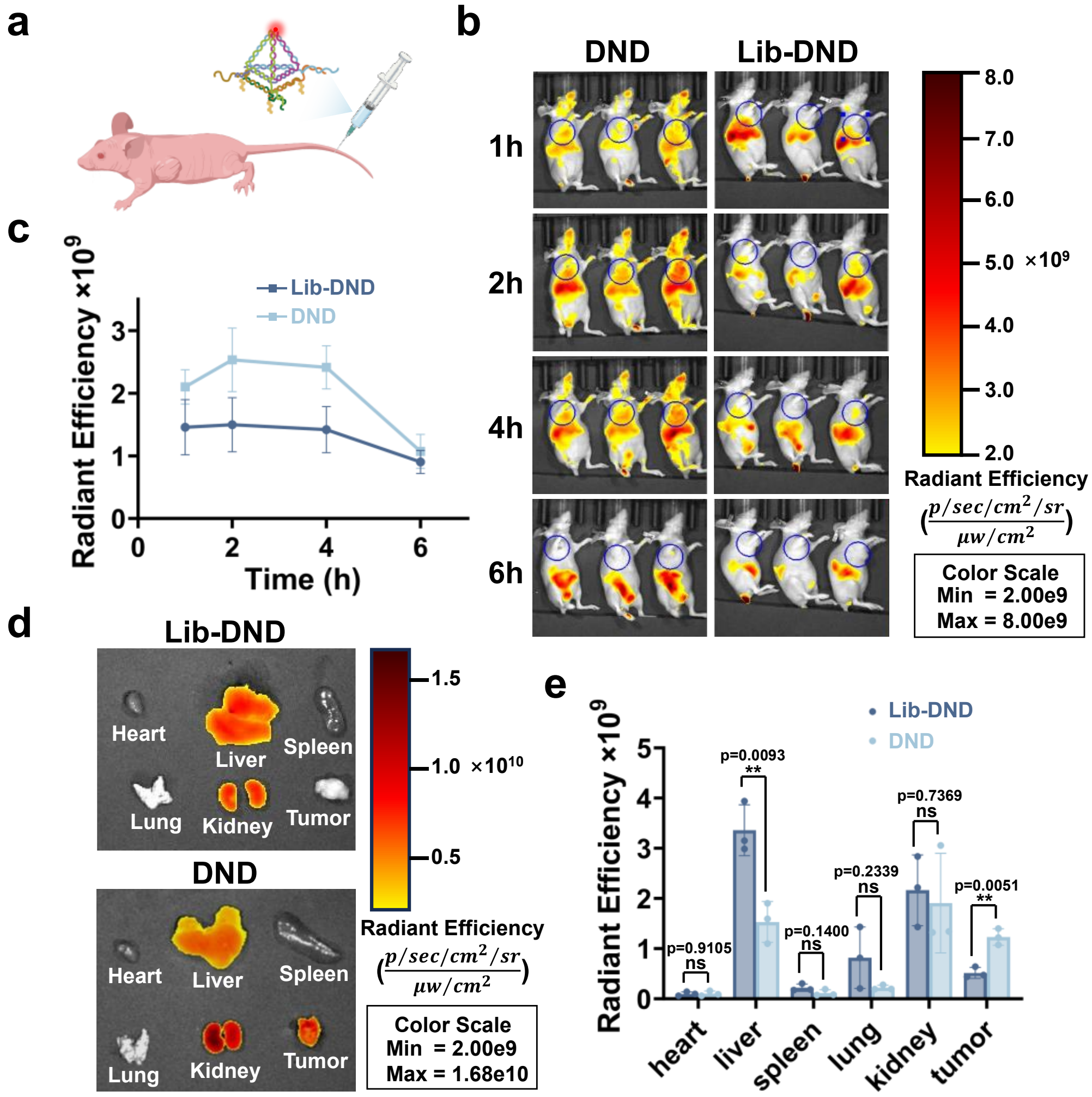

Supplementary Figure 28. Biodistribution of DND and Lib-DND after tail-vein injection. **(a)** Schematic of tail-vein injection of Cy5-labeled DND or Lib-DND into A549 tumor-bearing mice. **(b)** Whole-body fluorescence images acquired at 1, 2, 4, and 6 h after tail-vein injection; tumor regions are indicated by circles. **(c)** Time-dependent fluorescence intensity of the circled tumor regions in (b). **(d)** Ex vivo fluorescence images of excised tumors and major organs 2 h after tail-vein injection of DND or Lib-DND. **(e)** Quantitative fluorescence intensity of organs and tumors. Data are presented as mean ± SD (*n* = 3 biologically independent mice per group). Statistical significance was calculated using a two-tailed unpaired Welch's t-test. ***P* < 0.01, ns *P* > 0.05. Source data are provided as a Source Data file.

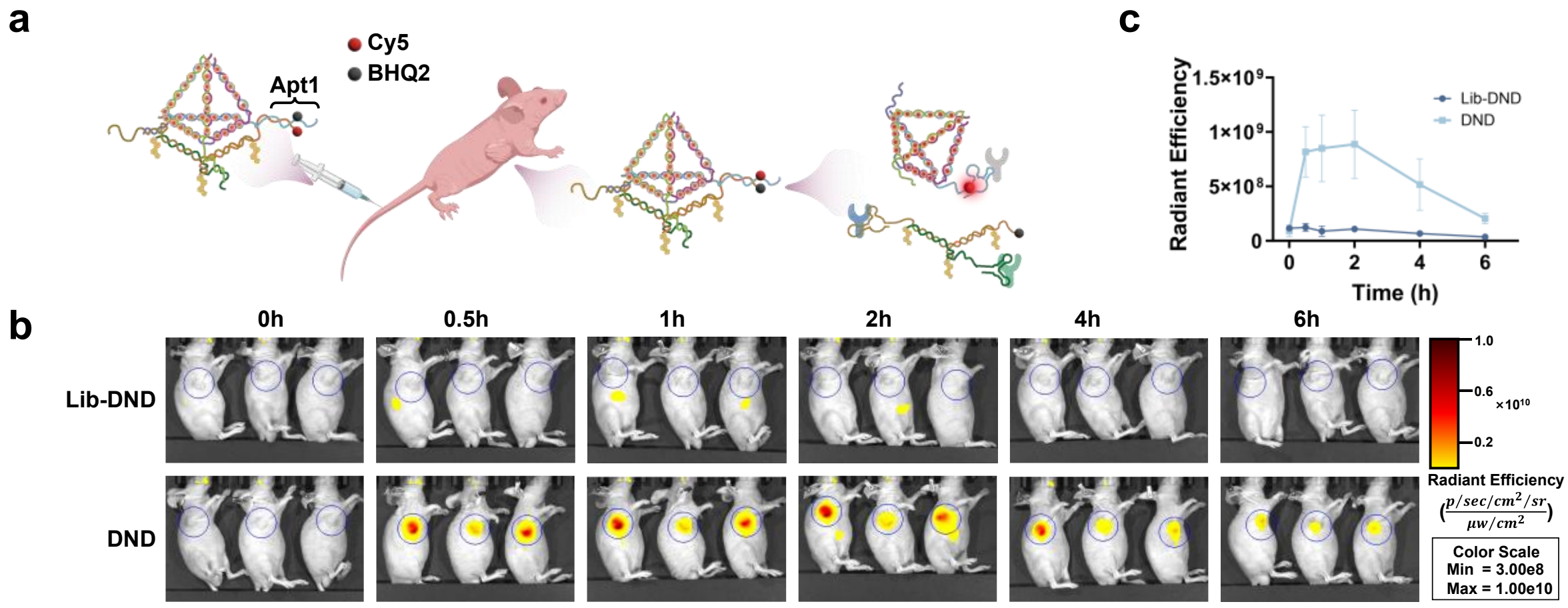

Supplementary Figure 29. In vivo fluorescence reporting of tumor-associated activation behavior of DND. **(a)** Schematic illustration of the fluorescence-reporting DND construct. Cy5 was labeled on the Apt1 strand and BHQ2 was labeled on the corresponding lock strand. In the assembled state, the Cy5 signal was quenched, whereas fluorescence recovery was observed after DND reached the tumor region and interacted with target cells. **(b)** Representative whole-body fluorescence images of tumor-bearing mice after intravenous injection of Lib-DND or DND at the indicated time points. Tumor regions are marked with circles. **(c)** Quantitative analysis of fluorescence intensity in the tumor region over time. Data are presented as mean ± SD (*n* = 3 biologically independent mice per group). Source data are provided as a Source Data file.

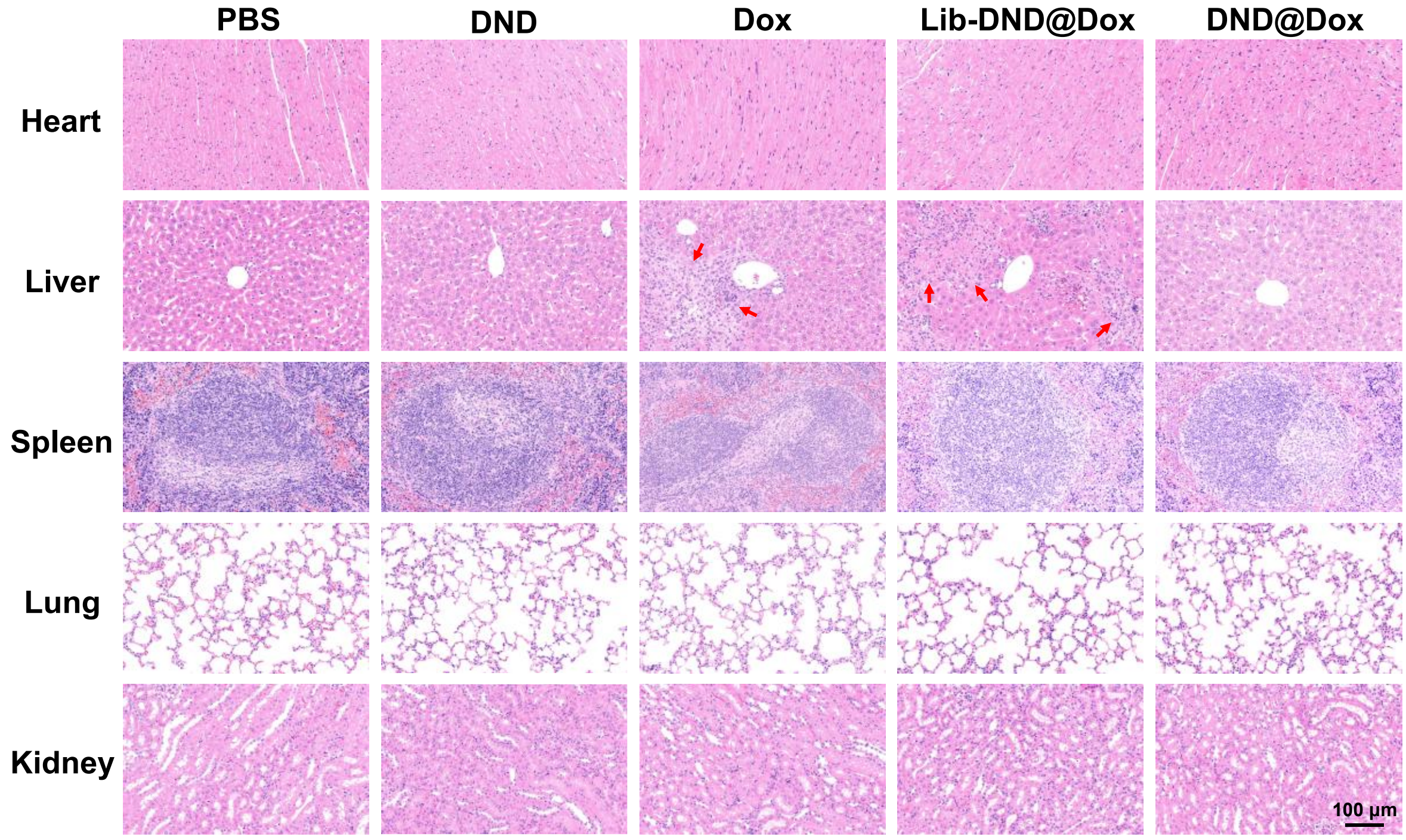

Supplementary Figure 30. H&E staining of major organs after intravenous treatment.Representative sections from each group. Arrows indicate representative histological alterations. Representative images are shown from five biologically independent mice per group. Scale bar, 100 μm. Source data are provided as a Source Data file.

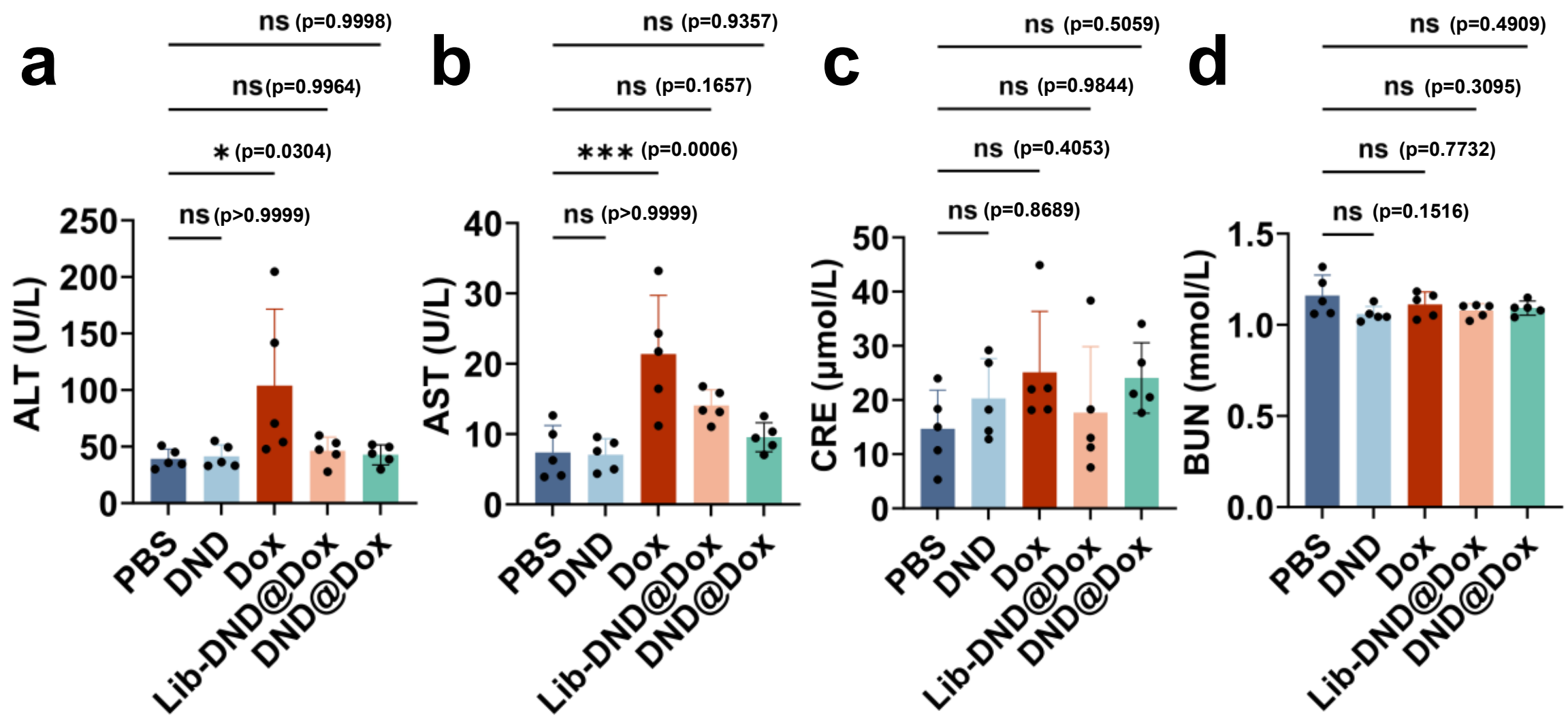

Supplementary Figure 31. Serum biochemical indices of mice after intravenous treatment. **(a)** ALT, **(b)** AST, **(c)** CRE, and **(d)** BUN levels. Data are presented as mean ± SD (*n* = 5 biologically independent mice per group). Statistical significance was evaluated using ordinary one-way ANOVA followed by Tukey's multiple comparisons test; all tests were two-sided, and P values were adjusted for multiple comparisons. $^*P < 0.05$, $^{***}P < 0.001$, ns $P > 0.05$. Source data are provided as a Source Data file.

Section C: Supplementary tables

Supplementary Table 1. The sequences of oligonucleotides used in this work [a)].

Name		Sequence (5'-3')
DND-1	t-s1	*ATCTAACTGCTGCGCCGCCGGGAAAATACTGTACGGTTAGA*TTTTTACATTCCTAAGTCTGAAACATTACAGCTTGCTACACGAGAAGAGCCGCCATAGTA
	t-s2	TCACTTATCTCCTGTCCCCCTGTTTTTTTATCACCAGGCAGTTGACAGTGTAGCAAGCTGTAATAGATGCGAGGGTCCAATAC
	t-s3	CACCAAATGACGTTAGAAGTCATTTTTTTCAACTGCCTGGTGATAAAACGACACTACGTGGGAATCTACTATGGCGGCTCTTC
	t-s4	TTCAGACTTAGGAATGTGCTTCCCACGTAGTGTCGTTTGTATTGGACCCTCGCAT
	Y-s1	*ACCAAACACAGATGCAACCTGACTTCTAACGTCATTTGGTG*TTCCTCCGCAATACTAATGGGTATGGGATTGG-Cholesteryl
	Y-s2	TCTAACCGTACAGTATTTTCCCGGCGGCCCAATCCCATACCCAAAACAGGTTCTCTTCTC-Cholesteryl
	Y-s3	*ATCACTTATAACGAGTGCGGATGCAAACGCCAGACAGGGGGACAGGAGATAAGTGA*GAGAAGAGAACCTGTAAAGTATTGCGGAGGAA-Cholesteryl
	Y-s1(0chol)	*ACCAAACACAGATGCAACCTGACTTCTAACGTCATTTGGTG*TTCCTCCGCAATACTAATGGGTATGGGATTGG
	Y-s2(0chol)	TCTAACCGTACAGTATTTTCCCGGCGGCCCAATCCCATACCCAAAACAGGTTCTCTTCTC
	Y-s3(0chol)	*ATCACTTATAACGAGTGCGGATGCAAACGCCAGACAGGGGGACAGGAGATAAGTGA*GAGAAGAGAACCTGTAAAGTATTGCGGAGGAA
	t-s1-BHQ1	*ATCTAACTGCTGCGCCGCCGGGAAAATACTGTACGGTT**(BHQ1)**AGA*TTTTTACATTCCTAAGTCTGAAACATTACAGCTTGCTACACGAGAAGAGCCGCCATAGTA
	t-s2-Cy3	**Cy3-**TCACTTATCTCCTGTCCCCCTGTTTTTTTATCACCAGGCAGTTGACAGTGTAGCAAGCTGTAATAGATGCGAGGGTCCAATAC
	t-s3-Cy5	**Cy5-**CACCAAATGACGTTAGAAGTCATTTTTTTCAACTGCCTGGTGATAAAACGACACTACGTGGGAATCTACTATGGCGGCTCTTC
	Y-s1-BHQ2	*ACCAAACACAGATGCAACCTGACTTCTAACGTCATTTGGT**(BHQ2)**G*TTCCTCCGCAATACTAATGGGTATGGGATTGG-Cholesteryl
	Y-s2-FAM	**FAM-**TCTAACCGTACAGTATTTTCCCGGCGGCCCAATCCCATACCCAAAACAGGTTCTCTTCTC-Cholesteryl
	Y-s3-BHQ2	*ATCACTTATAACGAGTGCGGATGCAAACGCCAGACAGGGGGACAGGAGATAAGT**(BHQ2)**GA*GAGAAGAGAACCTGTAAAGTATTGCGGAGGAA-Cholesteryl
	t-s4-FAM	**FAM-**TTCAGACTTAGGAATGTGCTTCCCACGTAGTGTCGTTTGTATTGGACCCTCGCAT
	t-s4-Cy5	**Cy5-**TTCAGACTTAGGAATGTGCTTCCCACGTAGTGTCGTTTGTATTGGACCCTCGCAT

Complementary strand	C-TCO1	CACCAAATGACGTTAGAAGTCAGGTTGCAT
	C-Sgc4f	TCTAACCGTACAGTATTTTCCCGGCGGCGCAGCAGTTAGAT
	C-Sgc8c	TCACTTATCTCCTGTCCCCCTGTCTGGCGTTTGCATCCGCA
Aptamer	TCO1	ACCAAACACAGATGCAACCTGACTTCTAACGTCATTTGGTG
	Sgc8c	ATCTAACTGCTGCGCCGCCGGGAAAATACTGTACGGTTAGA
	Sgc4f	ATCACTTATAACGAGTGCGGATGCAAACGCCAGACAGGGGGACAGGAGATAAGTGA
	TCO1-Cy5	**Cy5**-ACCAAACACAGATGCAACCTGACTTCTAACGTCATTTGGTG
	Sgc8c-Cy5	**Cy5**-ATCTAACTGCTGCGCCGCCGGGAAAATACTGTACGGTTAGA
	Sgc4f-Cy5	**Cy5**-ATCACTTATAACGAGTGCGGATGCAAACGCCAGACAGGGGGACAGGAGATAAGTGA
DND-2	t-s1	*GGTGGTGGTGGTTGTGGTGGTGGTGG*TTTTTTACATTCCTAAGTCTGAAACATTACAGCTTGCTACACGAGAAGAGCCGCCATAGTA
	t-s2	CAGGCCAACCCCCCATGACAACGTTTTTTTTATCACCAGGCAGTTGACAGTGTAGCAAGCTGTAATAGATGCGAGGGTCCAATAC
	t-s3	CCAGGGTATCCAAATTTTTTTCAACTGCCTGGTGATAAAACGACACTACGTGGGAATCTACTATGGCGGCTCTTC
	t-s4	TTCAGACTTAGGAATGTGCTTCCCACGTAGTGTCGTTTGTATTGGACCCTCGCAT
	Y-s1	*GCAGTTGATCCTTTGGATACCCTGG*TTTTTTTTCCTCCGCAATACTAATGGGTATGGGATTGG-Cholesteryl
	Y-s2	*CCACCACCACCACAA*TTTTTTCCAATCCCATACCCAAAACAGGTTCTCTTCTC-Cholesteryl
	Y-s3	*CACTACAGAGGTTGCGTCTGTCCCACGTTGTCATGGGGGGTTGGCCTG*TTTTTTGAGAAGAGAACCTGTAAAGTATTGCGGAGGAA-Cholesteryl
	t-s1-BHQ1	*GGTGGTGGTGGTTGTGGTGGTGGT**(BHQ1)**GG*TTTTTTACATTCCTAAGTCTGAAACATTACAGCTTGCTACACGAGAAGAGCCGCCATAGTA
	t-s2-Cy3	**Cy3-**CAGGCCAACCCCCCATGACAACGTTTTTTTTATCACCAGGCAGTTGACAGTGTAGCAAGCTGTAATAGATGCGAGGGTCCAATAC
	t-s3-Cy5	**Cy5-**CCAGGGTATCCAAATTTTTTTCAACTGCCTGGTGATAAAACGACACTACGTGGGAATCTACTATGGCGGCTCTTC
	Y-s1-BHQ2	*GCAGTTGATCCTTTGGATACCCT**(BHQ2)**GG*TTTTTTTTCCTCCGCAATACTAATGGGTATGGGATTGG-Cholesteryl
	Y-s2-FAM	**FAM-**CCACCACCACCACAATTTTTTCCAATCCCATACCCAAAACAGGTTCTCTTCTC-Cholesteryl
	Y-s3-BHQ2	*CACTACAGAGGTTGCGTCTGTCCCACGTTGTCATGGGGGGTTGGCCTG*TTTTTT**(BHQ2)**GAGAAGAGAACCTGTAAAGTATTGCGGAGGAA-Cholesteryl

	t-s4-CY5	**Cy5**-TTCAGACTTAGGAATGTGCTTCCCACGTAGTGTCGTTTGTATTGGACCCTCGCAT
	t-s1-Cy5	*GGTGGTGGTGGTTGTGGTGGTGGT**(Cy5)**GG*TTTTTTACATTCCTAAGTCTGAAACATTACAGCTTGCTACACGAGAAGAGCCGCCATAGTA
	Y-s2-BHQ2	**BHQ2-**CCACCACCACCACAATTTTTTCCAATCCCATACCCAAAACAGGTTCTCTTCTC-Cholesteryl
Complementary strand	C-MUC1	CCAGGGTATCCAAAGGATCAAC
	C-EpCAM	CAGGCCAACCCCCCATGACAACG
	C-AS1411	CCACCACCACCACAACCACC
Aptamer	MUC1	GCAGTTGATCCTTTGGATACCCTGG
	EpCAM	CACTACAGAGGTTGCGTCTGTCCCACGTTGTCATGGGGGGTTGGCCTG
	AS1411	GGTGGTGGTGGTTGTGGTGGTGGTGG
3apt-tFNA@Dox	t-s1	*ATCTAACTGCTGCGCCGCCGGGAAAATACTGTACGGTTAGA*TTTTTTACATTCCTAAGTCTGAAACATTACAGCTTGCTACACGAGAAGAGCCGCCATAGTA
	t-s2	*ATCACTTATAACGAGTGCGGATGCAAACGCCAGACAGGGGGACAGGAGATAAGTGA*TTTTTTTATCACCAGGCAGTTGACAGTGTAGCAAGCTGTAATAGATGCGAGGGTCCAATAC
	t-s3	*ACCAAACACAGATGCAACCTGACTTCTAACGTCATTTGGTG*TTTTTTTCAACTGCCTGGTGATAAAACGACACTACGTGGGAATCTACTATGGCGGCTCTTC
	t-s4-Cy5	**Cy5-**TTCAGACTTAGGAATGTGCTTCCCACGTAGTGTCGTTTGTATTGGACCCTCGCAT

[a] The italicized part is the aptamer sequence. The underlined part is the section that specifically binds to the aptamer.

Supplementary Table 2. Quantitative parameters for CEM cell detection in a mouse blood-cell background.

Theoretical tumor cells	Nominal fraction (%)	Detected tumor cells[a]	Detected proportion (%)[a]	Recovery (%)[b]	RSD (%)[c]
0	0	0.33 ± 0.58	0.003 ± 0.006%	—	—
25	0.25	19.33 ± 3.21	0.193 ± 0.032%	77.33	16.63
50	0.50	49.67 ± 8.08	0.497 ± 0.081%	99.33	16.27
100	1.00	94.00 ± 18.08	0.940 ± 0.181%	94	19.24
500	5.00	426.33 ± 68.24	4.263 ± 0.682%	85.27	16.01
1000	10.00	918.00 ± 53.81	9.180 ± 0.538%	91.8	5.86
3000	30.00	2888.67 ± 3.21	28.887 ± 0.032%	96.29	0.11
5000	50.00	4791.67 ± 22.59	47.917 ± 0.226%	95.83	0.47
10000	100.00	9522.67 ± 132.99	95.227 ± 1.330%	95.23	1.4

[a] Data are presented as mean ± SD from three independent experiments.

[b] Recovery (%) = $N_{\mathrm{detected}} / N_{\mathrm{theoretical}} \times 100\%$

[c] RSD, relative standard deviation.

Section D: Uncropped gels

Supplementary Fig. 10

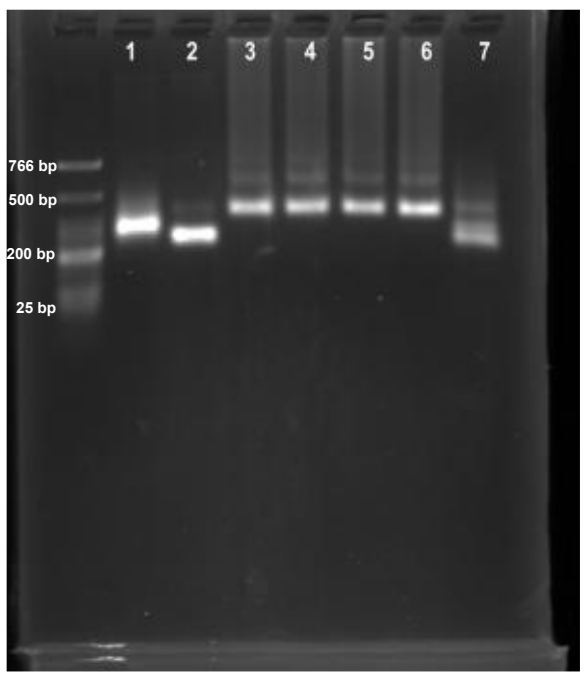

Supplementary Fig. 12

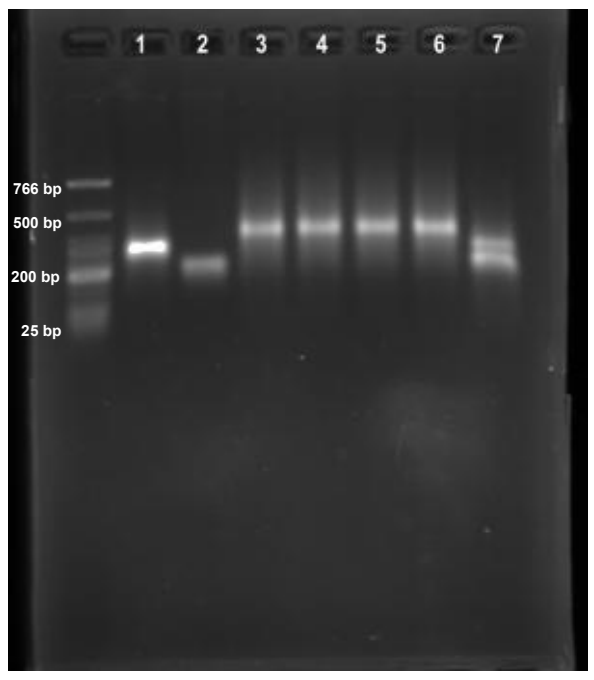

Supplementary Fig. 16

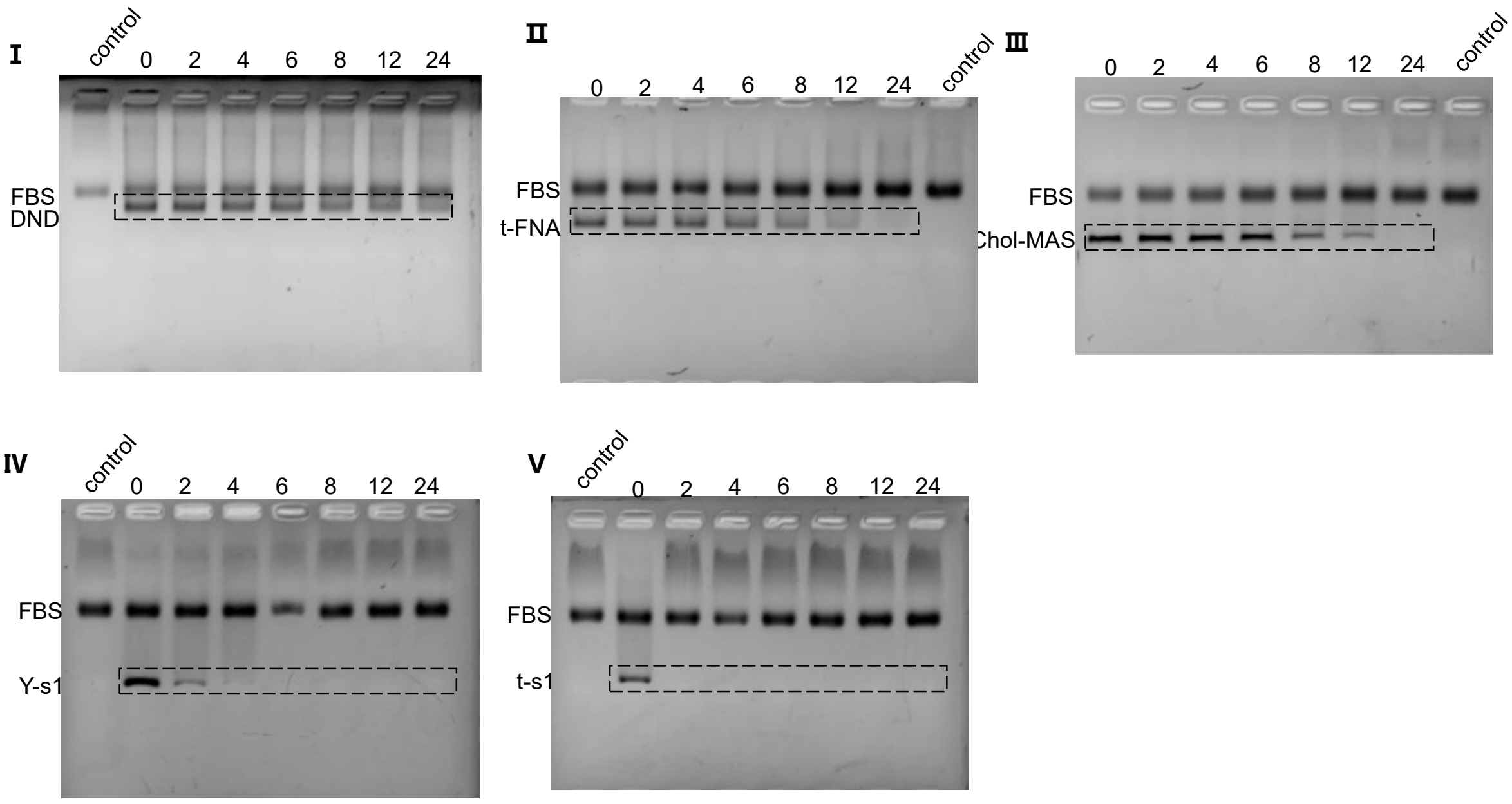

Section E: Supplementary References

1. Ouyang, C. et al. Precision-Guided Missile-Like DNA Nanostructure Containing Warhead and Guidance Control for Aptamer-Based Targeted Drug Delivery into Cancer Cells in Vitro and in Vivo. *J. Am. Chem. Soc.* **142**, 1265-1277 (2020).
2. Šulc, P. et al. Sequence-dependent thermodynamics of a coarse-grained DNA model. *J. Chem. Phys.* **137**, 135101 (2012).
3. Abraham, M. J. et al. GROMACS: High performance molecular simulations through multi-level parallelism from laptops to supercomputers. *SoftwareX* **1-2**, 19-25 (2015).
4. He, A. et al. Structure-based investigation of a DNA aptamer targeting PTK7 reveals an intricate 3D fold guiding functional optimization. *Proc. Natl Acad. Sci. USA* **121**, e2404060121 (2024).
5. Abramson, J. et al. Accurate structure prediction of biomolecular interactions with AlphaFold 3. *Nature* **630**, 493-500 (2024).
6. Yan, Y., Wen, Z., Wang, X. & Huang, S. Y. Addressing recent docking challenges: A hybrid strategy to integrate template-based and free protein-protein docking. *Proteins* **85**, 497-512 (2017).
7. Yan, Y., Zhang, D., Zhou, P., Li, B. & Huang, S. Y. HDOCK: a web server for protein-protein and protein-DNA/RNA docking based on a hybrid strategy. *Nucleic Acids Res.* **45**, W365-W373 (2017).
8. Yan, Y., Tao, H., He, J. & Huang, S. Y. The HDOCK server for integrated protein-protein

docking. *Nat. Protoc.* **15**, 1829-1852 (2020).
