# Supplementary material for: Membrane-anchored DNA nanodevice with allosteric aptamer arms enables parallel probing and on-site drug delivery
Source: Nat Commun. 2026 Jul 7;17:8394. doi: 10.1038/s41467-026-75300-5 (PMC13473122; doi:10.1038/s41467-026-75300-5)
Supplement: Supplementary file 2 [file 41467_2026_75300_MOESM2_ESM.pdf]

nature portfolio

Corresponding author(s): Chang Xue

Last updated by author(s): Jun 1, 2026

# Reporting Summary

Nature Portfolio wishes to improve the reproducibility of the work that we publish. This form provides structure for consistency and transparency in reporting. For further information on Nature Portfolio policies, see our Editorial Policies and the Editorial Policy Checklist.

## Statistics

For all statistical analyses, confirm that the following items are present in the figure legend, table legend, main text, or Methods section.

| n/a | Confirmed | |
|---|---|---|
| ☐ | ☒ | The exact sample size ($n$) for each experimental group/condition, given as a discrete number and unit of measurement |
| ☐ | ☒ | A statement on whether measurements were taken from distinct samples or whether the same sample was measured repeatedly |
| ☐ | ☒ | The statistical test(s) used AND whether they are one- or two-sided<br>*Only common tests should be described solely by name; describe more complex techniques in the Methods section.* |
| ☒ | ☐ | A description of all covariates tested |
| ☐ | ☒ | A description of any assumptions or corrections, such as tests of normality and adjustment for multiple comparisons |
| ☐ | ☒ | A full description of the statistical parameters including central tendency (e.g. means) or other basic estimates (e.g. regression coefficient) AND variation (e.g. standard deviation) or associated estimates of uncertainty (e.g. confidence intervals) |
| ☐ | ☒ | For null hypothesis testing, the test statistic (e.g. $F$, $t$, $r$) with confidence intervals, effect sizes, degrees of freedom and $P$ value noted<br>*Give P values as exact values whenever suitable.* |
| ☒ | ☐ | For Bayesian analysis, information on the choice of priors and Markov chain Monte Carlo settings |
| ☒ | ☐ | For hierarchical and complex designs, identification of the appropriate level for tests and full reporting of outcomes |
| ☒ | ☐ | Estimates of effect sizes (e.g. Cohen's $d$, Pearson's $r$), indicating how they were calculated |

*Our web collection on statistics for biologists contains articles on many of the points above.*

## Software and code

Policy information about availability of computer code

| | |
|---|---|
| Data collection | Image Lab 6.1.0, CytExpert 2.4, NIS Elements 5.21 and IVIS Living Image 4.5.5 were used to collect data. |
| Data analysis | Gwyddion 2.70, GraphPad Prism 10.1.2, ImageJ 1.54, FlowJo 10.8.1, oxDNA Viewer, GROMACS 2024.2, AlphaFold 3 and HDOCK were used to analyze data and perform computational modeling. |

For manuscripts utilizing custom algorithms or software that are central to the research but not yet described in published literature, software must be made available to editors and reviewers. We strongly encourage code deposition in a community repository (e.g. GitHub). See the Nature Portfolio guidelines for submitting code & software for further information.

## Data

Policy information about availability of data

All manuscripts must include a data availability statement. This statement should provide the following information, where applicable:

- Accession codes, unique identifiers, or web links for publicly available datasets
- A description of any restrictions on data availability
- For clinical datasets or third party data, please ensure that the statement adheres to our policy

The data generated or analyzed during this study are included in this article, the Supplementary Information, and the Source Data file. Source data are provided for Figs. 2b–e, h, j, 3c–f, 6c–f, 7b, c, e–g and Supplementary Figs. 4, 6, 10, 12c, 14, 15b–g, 16, 17b, 19–23, 26b, d–g, 27, 28c, e, 29–31. Source data are provided with this paper.

## Research involving human participants, their data, or biological material

Policy information about studies with human participants or human data. See also policy information about sex, gender (identity/presentation), and sexual orientation and race, ethnicity and racism.

| | |
|---|---|
| Reporting on sex and gender | N/A |
| Reporting on race, ethnicity, or other socially relevant groupings | N/A |
| Population characteristics | N/A |
| Recruitment | N/A |
| Ethics oversight | N/A |

Note that full information on the approval of the study protocol must also be provided in the manuscript.

# Field-specific reporting

Please select the one below that is the best fit for your research. If you are not sure, read the appropriate sections before making your selection.

☒ Life sciences ☐ Behavioural & social sciences ☐ Ecological, evolutionary & environmental sciences

For a reference copy of the document with all sections, see nature.com/documents/nr-reporting-summary-flat.pdf

# Life sciences study design

All studies must disclose on these points even when the disclosure is negative.

| | |
|---|---|
| Sample size | The sample size was determined based on previous related studies and similar experimental designs. For in vitro assays, three independent samples or experiments were generally used unless otherwise stated. For animal experiments, four biologically independent mice were used per group for peritumoral injection therapy, five biologically independent mice were used per group for tail-vein injection therapy, and three biologically independent mice were used per group for in vivo imaging experiments. No statistical method was used to predetermine sample size. |
| Data exclusions | No data were excluded from the analyses. |
| Replication | All attempts at replication were successful. Key experiments, including flow cytometry, confocal laser scanning microscopy (CLSM), fluorescence spectroscopy and agarose gel electrophoresis, were independently repeated at least three times with similar results. |
| Randomization | All the groups in this study are random. |
| Blinding | The investigators were blinded to group allocation during data collection and analysis. |

# Reporting for specific materials, systems and methods

We require information from authors about some types of materials, experimental systems and methods used in many studies. Here, indicate whether each material, system or method listed is relevant to your study. If you are not sure if a list item applies to your research, read the appropriate section before selecting a response.

### Materials & experimental systems

| n/a | Involved in the study | |
|---|---|---|
| ☒ | ☐ | Antibodies |
| ☐ | ☒ | Eukaryotic cell lines |
| ☒ | ☐ | Palaeontology and archaeology |
| ☐ | ☒ | Animals and other organisms |
| ☒ | ☐ | Clinical data |
| ☒ | ☐ | Dual use research of concern |
| ☒ | ☐ | Plants |

### Methods

| n/a | Involved in the study | |
|---|---|---|
| ☒ | ☐ | ChIP-seq |
| ☐ | ☒ | Flow cytometry |
| ☒ | ☐ | MRI-based neuroimaging |

## Eukaryotic cell lines

Policy information about cell lines and Sex and Gender in Research

| | |
|---|---|
| Cell line source(s) | CEM, Ramos, HeLa, A549, HepG2, HELF were obtained from Institute of Biochemistry and Cell Biology, Chinese Academy of Science (Shanghai, China). IOSE80 cell was obtained from Shanghai Fuheng Biotechnology Co., Ltd. |
| Authentication | All cell lines were obtained from recognized cell banks or commercial suppliers. STR profiling was performed to authenticate the cell lines before use. |
| Mycoplasma contamination | All the cell lines were tested negative for mycoplasma contamination. |
| Commonly misidentified lines (See ICLAC register) | None |

## Animals and other research organisms

Policy information about studies involving animals; ARRIVE guidelines recommended for reporting animal research, and Sex and Gender in Research

| | |
|---|---|
| Laboratory animals | BALB/c nude mice (Mus musculus, BALB/c genetic background, 3–4 weeks old) were obtained from Zhejiang Vital River Laboratory Animal Technology Co., Ltd. (Zhejiang, China). The mice were housed under specific pathogen-free conditions at an ambient temperature of 25 °C and a relative humidity of 40–60%, under a 12 h light/dark cycle, with free access to food and water. |
| Wild animals | None |
| Reporting on sex | Both female and male mice were used in this study. Specifically, female BALB/c nude mice were used for the primary in vivo experiments, while male mice were used for the supplementary experiments (Supplementary Figure 29). Sex was not considered as a biological variable for statistical comparison in the study design, as the fundamental physical and chemical mechanisms of the DNA nanostructure assembly, logic-gated targeting, and drug release are not expected to be sex-dependent. |
| Field-collected samples | This study did not involve samples collected from the field. |
| Ethics oversight | All animal experiments were performed under the protocol approved by the Laboratory Animal Ethics Committee of Wenzhou Medical University (approval number: wydw2024-0453). |

Note that full information on the approval of the study protocol must also be provided in the manuscript.

## Plants

| | |
|---|---|
| Seed stocks | N/A |
| Novel plant genotypes | N/A |
| Authentication | N/A |

## Flow Cytometry

### Plots

Confirm that:

- ☒ The axis labels state the marker and fluorochrome used (e.g. CD4-FITC).
- ☒ The axis scales are clearly visible. Include numbers along axes only for bottom left plot of group (a 'group' is an analysis of identical markers).
- ☒ All plots are contour plots with outliers or pseudocolor plots.
- ☒ A numerical value for number of cells or percentage (with statistics) is provided.

### Methodology

| | |
|---|---|
| Sample preparation | For flow cytometry assays, suspension cells were collected by centrifugation after incubation, whereas adherent cells were detached when necessary. Cells were washed three times with cold PBS to remove unbound DND or drug, resuspended in |

| | |
|---|---|
| | 300–500 µL cold PBS, and analyzed immediately by flow cytometry. |
| Instrument | CytoFLEX S flow cytometer (Beckman Coulter). |
| Software | CytExpert v2.4 software was used for data collection, and FlowJo v10.8.1 software was used for data analysis. |
| Cell population abundance | Not applicable. Cell sorting was not performed in this study; flow cytometry was used strictly for fluorescence analysis. |
| Gating strategy | Initial gating was performed using Forward Scatter Area (FSC-A) versus Side Scatter Area (SSC-A) to exclude cell debris and identify the main intact cell population. Single cells were subsequently gated using FSC-A versus FSC-Height (FSC-H) to exclude doublets. The positive and negative fluorescence boundaries/gates for specific channels (e.g., Cy5, FITC) were strictly established based on untreated (blank) control cells. |

☒ Tick this box to confirm that a figure exemplifying the gating strategy is provided in the Supplementary Information.
