# Supplementary material for: Membrane-anchored DNA nanodevice with allosteric aptamer arms enables parallel probing and on-site drug delivery
Source: Nat Commun. 2026 Jul 7;17:8394. doi: 10.1038/s41467-026-75300-5 (PMC13473122; doi:10.1038/s41467-026-75300-5)
Supplement: Supplementary file 3 [file 41467_2026_75300_MOESM3_ESM.pdf]

nature portfolio

Peer Review File

Membrane-Anchored DNA Nanodevice with Allosteric Aptamer Arms Enables Parallel Probing and On-Site Drug Delivery

Corresponding Author: Dr Chang Xue

This file contains all reviewer reports in order by version, followed by all author rebuttals in order by version.

Version 0:

Reviewer comments:

Reviewer #1

(Remarks to the Author)
This manuscript describes the development of a "DNA nano-octopus" (DNO) system, which combines a Dox-loaded DNA tetrahedron carrier with a Y-shaped cholesterol-modified membrane-anchored scaffold (Chol-MAS) for cancer cell capture and drug delivery. While the concept of multi-aptamer integration is interesting, the current design appears unnecessarily complex compared to existing aptamer-tethered DNA nanostructures. The reviewer has several fundamental concerns regarding the mechanistic claims, the necessity of the chosen architecture, and the lack of critical controls (see below). Furthermore, the therapeutic efficacy of DNO@Dox in both cellular environments (Figure 5c) and animal models (Figures 6c–f) shows marginal improvement over free DNO or the Lib-DNO@Dox control.

Major concerns:
1. The authors characterize the system as an "AND gate" operation. However, based on the data in Figure 2, the fluorescence signal is "ON" regardless of whether one, two, or three inputs are present. This behavior describes an OR gate (or simple multivalent binding), not an AND gate.
2. The authors claim "spatially precise drug release." This statement is inaccurate, as drug release appears to be a passive result of tetrahedron dissociation after cellular uptake rather than a triggered response to an active stimulus.
3. A critical missing control is a Dox-loaded DNA tetrahedron functionalized directly with the three aptamers (without the Chol-MAS). This control is essential to justify the complexity of the "octopus" design and to demonstrate whether the membrane-anchored scaffold provides any significant advantage in probing sensitivity or anti-tumor activity.
4. The electrophoretic mobility shift in Figure 1b (lane 8) is insufficient to distinguish the DNA nano-octopus from the standard tetrahedron. The resolution of AFM and TEM imaging is inadequate. Given the potential for dehydration artifacts in dry-state imaging, Cryo-EM should be provided to accurately confirm the dimensions and integrity of the nanostructures in solution.
5. What is the specific functional necessity of the cholesterol anchor? Please provide data showing the system's performance in the absence of cholesterol to clarify its role in cell-surface localization.
6. In Figure 1i, the initial fluorescence intensities across the three images are inconsistent. This discrepancy makes it difficult to quantitatively compare the samples before and after bleaching or to assess the recovery rate at 140s.
7. In Figure 2c, the DNA band in lane 1 is significantly diffused. Please provide an explanation for this lack of stability or poor resolution.
8. In Figure 3b, the Cy5 signal is disproportionately strong and appears to extend beyond the cell membrane boundaries. Is this an artifact of signal saturation, or does it indicate a leakage problem?
9. In Figure 3d, the authors should monitor the same field of view/cell population to track the release and uptake process over time. Comparing different spots at different time points makes the data less convincing.
10. Several figures mentioned in the results (Figures S11e–f and 5c–f) appear to be missing from the provided main text or supplementary sections.
11. The statement on page 21 (line 382) regarding apoptosis is not supported by Figure 5c, as the difference between DNO and DNO@Dox appears negligible. Rigorous statistical analysis must be performed and indicated for all data in Figures 5c– f.
12. The claim of "dramatic tumor regression" (page 24) is not supported by Figure 6c. Additionally, there is a significant discrepancy between the efficacy of peritumoral treatment versus intravenous administration that needs addressing.

13. The data in Figures S20–S21 appear redundant, as similar experiments involving tail vein injections are already presented in Figure 6 and Figures S22–S23.
14. Please correct the erroneous labels in Figure 6e.

Reviewer #2

(Remarks to the Author)
Major revisions.

This manuscript reports a membrane-anchored biomimetic DNA nano-octopus featuring multiple aptamer arms that are described as allosterically regulated for parallel molecular probing and on-site drug release. The overall concept is creative and timely, integrating DNA nanotechnology with membrane-localized sensing and responsive therapeutic delivery. The work has potential interest for the broad readership of Nature Communications. However, several key aspects of the mechanistic basis, quantitative performance, and biological relevance are not yet sufficiently substantiated. Several points would benefit from clarification and additional quantitative support to further strengthen the manuscript.
1. The manuscript refers to the aptamer arms as allosterically regulated. While the presented data are consistent with target-induced functional responses, additional clarification of the underlying allosteric mechanism would improve mechanistic rigor. A clearer definition and, if possible, supporting structural or spectroscopic evidence would strengthen this central claim.
2. The manuscript highlights parallel probing as a key advantage of the nano-octopus architecture. However, the potential for signal interference among different aptamer arms is not systematically evaluated. The authors are encouraged to provide additional evaluations under multi-target conditions, to further support this claim.
3. As the sensor system showed excellent performance, I wonder if the authors could try to provide some quantitative sensing parameters (e.g., detection limits, linear response ranges, and reproducibility).
4. Some figures (e.g., Figure 1c–e and Figure 4c) would benefit from improved resolution and clearer annotations.
5. In Scheme 1, some labels are not consistently capitalized. Please unify the formatting to improve clarity and presentation consistency.
6. To further support the proposed allosteric mechanism, theoretical modeling or molecular simulations (e.g., MD or coarse-grained simulations) could be highly valuable in elucidating the conformational coupling between target binding and functional responses of the aptamer arms.
7. Minor language polishing is recommended.
8. Does high concentration of DNO@Dox have cytotoxicity? What is the safe concentration range or IC50 value?

9. It is highly necessary to clarify the kinetics of DNO@Dox release in vivo and the enrichment rate at the tumor site. Please supplement this part.

10. The difference in the in vivo effects between Lib-DNO@Dox and DNO@Dox is due to the targeting sequences of the aptamers or the release effect?

11. Please check if the annotations in Figure 6e (Lib-BNO@Dox, BNO@Dox) are incorrect.

12. A single aptamer is generally sufficient for target recognition. The rationale and mechanistic advantages of using three aptamer arms instead of one are not clearly explained and should be clarified.

Overall, this study presents an interesting and potentially impactful platform. With major revisions addressing the points above, this manuscript would represent a strong and timely contribution to the field and is suitable for publication in Nature Communications.

Version 1:

Reviewer comments:

Reviewer #1

(Remarks to the Author)
No further comments.

Reviewer #2

(Remarks to the Author)
I recommend acceptance of this paper.It is well written and I have no comments.

Open Access This Peer Review File is licensed under a Creative Commons Attribution 4.0 International License, which permits use, sharing, adaptation, distribution and reproduction in any medium or format, as long as you give appropriate credit to the original author(s) and the source, provide a link to the Creative Commons license, and indicate if changes were made.
In cases where reviewers are anonymous, credit should be given to 'Anonymous Referee' and the source.
The images or other third party material in this Peer Review File are included in the article's Creative Commons license, unless indicated otherwise in a credit line to the material. If material is not included in the article's Creative Commons license and your intended use is not permitted by statutory regulation or exceeds the permitted use, you will need to obtain permission directly from the copyright holder.
To view a copy of this license, visit https://creativecommons.org/licenses/by/4.0/

Response to Editor and reviewers

Response to the Editor:

We sincerely thank the editor for the careful evaluation of our manuscript and for the opportunity to submit a revised version. We have carefully addressed all reviewer comments point by point and revised the manuscript, Supplementary Information, figures, and related files accordingly. In addition, we have followed the editorial requirements by updating the Data Availability section, preparing the Source Data file, completing the Reporting Summary, and checking the chemical and biomolecular materials reporting requirements. We have also revised the relevant figures and legends to show underlying data points and describe measures of centrality and dispersion where appropriate. The corresponding author will ensure that the ORCID information is properly linked in the Manuscript Tracking System. There are no changes to the author list in this revision.

Response to Reviewer 1:

This manuscript describes the development of a "DNA nano-octopus" (DNO) system, which combines a Dox-loaded DNA tetrahedron carrier with a Y-shaped cholesterol-modified membrane-anchored scaffold (Chol-MAS) for cancer cell capture and drug delivery. While the concept of multi-aptamer integration is interesting, the current design appears unnecessarily complex compared to existing aptamer-tethered DNA nanostructures. The reviewer has several fundamental concerns regarding the mechanistic claims, the necessity of the chosen architecture, and the lack of critical controls (see below). Furthermore, the therapeutic efficacy of DNO@Dox in both cellular environments (Figure 5c) and animal models (Figures 6c–f) shows marginal improvement over free DNO or the Lib-DNO@Dox control.

Response: We thank the reviewer for the constructive overall assessment. In response, we have substantially revised the manuscript to address the concerns raised, focusing on three key aspects:

1. Addition of critical controls to validate mechanistic claims and architectural necessity. Following the reviewer's suggestion, we synthesized and characterized two new control constructs— 3apt-tFNA@Dox (three aptamers directly conjugated to the tetrahedron without Chol-MAS) and 0chol-DNO@Dox (retains the Y-shaped scaffold but lacks cholesterol modification). Comparative analysis reveals that direct aptamer conjugation leads to non-discriminatory uptake in any cell expressing even a single target receptor, while removal of cholesterol anchoring only partially suppresses non-specific internalization. Only the full DNO@Dox architecture achieves stringent cell-type discrimination, confirming that the Chol-MAS scaffold and cholesterol-mediated membrane anchoring are functionally indispensable for enforcing surface confinement and enabling multi-input recognition before cargo release.
2. Correction of statistical errors and refinement of therapeutic data presentation. We discovered that while the raw flow cytometry data in Figure 5b were correct, the statistical analysis presented in Figure 5c in the original manuscript contained errors. We have now re-analyzed the apoptosis data, updated the figures, and revised the accompanying text. The corrected data demonstrate that DNO@Dox achieves potent and statistically significant apoptosis induction in target CEM cells, while maintaining a markedly improved safety profile. We have also carefully examined Figures 6c–f, and we respectfully clarify the interpretation of the in vivo therapeutic efficacy. The data show distinct and therapeutically meaningful differences among the treatment groups: free

DNO (drug-free carrier), Lib-DNO@Dox (non-targeting control), and free Dox all exhibited continued tumor growth, with the free DNO group displaying the fastest tumor progression. In contrast, DNO@Dox effectively suppressed tumor growth, and statistical analysis confirmed significant differences from all control groups, indicating a clear therapeutic benefit (revised Figure 6c). Moreover, we clarified the distinction between peritumoral and intravenous administration routes to provide proper context for the in vivo efficacy data.

3. Refinement of mechanistic terminology. We have carefully revised the manuscript to eliminate terminological ambiguity: fluorescence signals are now described as independent recognition reporters rather than logic outputs; the AND-gated functional event is explicitly defined as DNO disassembly and tFNA@Dox release; and “spatially precise drug release” has been replaced with “spatially precise drug delivery.” These revisions ensure that mechanistic claims are precise and well-supported by the experimental evidence.

Detailed point-by-point responses follow below.

Major concerns:

1. The authors characterize the system as an "AND gate" operation. However, based on the data in Figure 2, the fluorescence signal is "ON" regardless of whether one, two, or three inputs are present. This behavior describes an OR gate (or simple multivalent binding), not an AND gate.

Response: We thank the reviewer for this critical observation, which highlights a potential ambiguity in our original wording. We fully acknowledge that the fluorescence readout from each individual aptamer arm operates in a parallel, non-interfering manner and each channel is activated independently upon binding its cognate target. However, we respectfully clarify that the “AND gate” designation refers specifically to the functional output of the system, i.e., the dissociation of the tetrahedral drug carrier (tFNA@Dox) from the membrane-anchored scaffold, which occurs only when all three aptamer arms are simultaneously engaged.

To eliminate any conceptual confusion, we have carefully revised the manuscript to make this distinction explicit:

1. Revised text (Results, section “Working Principle of a Biomimetic DNA Nano-Octopus for Parallel Probing and Logic-Gated Drug Delivery”): “The system was designed as a three-input AND gate with respect to its functional output (payload release), which is generated exclusively when all three inputs are present simultaneously, whereas each fluorescence channel independently reports the binding status of its corresponding aptamer arm.” (**Page 5, Lines 108–111**).
2. Revised text (Figure 2b legend): “(b) Truth table for the three-input AND gate. The functional output (Output = 1, defined as tFNA release) occurs only when all three inputs (A, B, C) are simultaneously present (1,1,1). The fluorescence channels (FAM, Cy3, Cy5) independently report the binding status of each aptamer arm and do not constitute the logic gate output themselves” (**Page 14, Lines 263–267**).

These clarifications ensure that readers understand the distinction between the independent fluorescence reporters and the AND-gated functional disassembly event.

2. The authors claim "spatially precise drug release." This statement is inaccurate, as drug release appears to be a passive result of tetrahedron dissociation after cellular uptake rather than a triggered response to an active stimulus.

Response: We sincerely thank the reviewer for this astute observation, which highlights a critical nuance in the nomenclature of multi-step drug delivery systems. We fully acknowledge that the phrasing in the original manuscript blurred the distinction between carrier disassembly (an active, stimulated process) and cargo liberation (a passive, downstream consequence). The reviewer is correct that the intercalated doxorubicin (Dox) itself is not cleaved or released by an external physical or chemical trigger; its liberation is a passive result of nuclease-mediated degradation of the DNA tetrahedron within the endo-lysosomal compartment.

However, the intended subject of the "spatially precise drug release" claim is not the Dox molecule, but the integrated tetrahedral drug carrier (tFNA@Dox) as a whole unit. To eliminate the ambiguity identified by the reviewer, we have carefully revised the manuscript:

1. Title (Revised): "A Membrane-Anchored Biomimetic DNA Nano-Octopus with Allosteric Aptamer Arms Enables Parallel Probing and On-Site Drug Delivery"
2. Abstract (Revised): "...an intelligent nanomachine that integrates multivalent recognition, logic-gated computation, and spatially precise drug delivery in a single nanostructure." (**Page 1, Line 10**)
3. Introduction (Revised): "The DNO integrates parallel multi-target sensing, Boolean computation, and spatially precise drug delivery within a single membrane-anchored nanostructure." (**Page 4, Line 80**)
4. Results (Revised): "Working Principle of Biomimetic DNA Nano-Octopus for Parallel Probing and Logic-Gated Drug Delivery". (**Page 4, Line 93**)

3. A critical missing control is a Dox-loaded DNA tetrahedron functionalized directly with the three aptamers (without the Chol-MAS). This control is essential to justify the complexity of the "octopus" design and to demonstrate whether the membrane-anchored scaffold provides any significant advantage in probing sensitivity or anti-tumor activity.

Response: We thank the reviewer for this insightful comment, which prompted us to experimentally deconvolute the functional contributions of the membrane-anchored scaffold. In the revised manuscript, we have synthesized and characterized a simplified control construct, designated 3apt-tFNA@Dox, in which all three aptamers (Sgc8c, Sgc4f, and TCO1) are directly conjugated to the tetrahedral framework without the Chol-MAS module. A comparative analysis of this control versus the full DNO@Dox system is now presented in new supplementary Figure S25a. To enable unambiguous tracking of the nanocarrier independent of the drug cargo, we labeled the tetrahedral vertex with Cy5, while simultaneously monitoring the intrinsic fluorescence of doxorubicin (Dox) to assess intracellular drug delivery. Additionally, to rigorously evaluate selectivity against normal tissue background, we expanded our cell panel to include IOSE80 cells, a normal human ovarian epithelial line that expresses the PTK7 receptor (target of the Sgc8c aptamer), as previously characterized (J. Am. Chem. Soc. **2020**, 142, 1265–1277).

The experimental results reveal a stark functional distinction between the two architectures:

1. 3apt-tFNA@Dox (No Scaffold): Robust intracellular Cy5 and Dox signals were detected not only in target CEM cells but also in Ramos and HeLa cells, which express only subsets of the three receptors. More critically, although uptake was minimal in HELF cells (negative for all three targets), significant internalization was observed in IOSE80 normal cells that express PTK7. These data indicate that in the absence of the Chol-MAS scaffold, engagement of

a single aptamer–receptor interaction is sufficient to trigger cellular internalization. Consequently, this simplified design lacks the discriminatory power required to distinguish tumor cells from normal cells that share a single surface marker.

2. DNO@Dox (With Chol-MAS Scaffold): Under identical conditions, the full DNO architecture exhibited markedly superior selectivity. Internalization of both the Cy5 -labeled carrier and Dox cargo was predominantly observed in CEM cells, which co-express the full complement of three receptors required to satisfy the AND-logic gate.

Therefore, the apparent complexity of the "DNA nano-octopus" design is not merely structural but is functionally essential. We have incorporated these data and the corresponding discussion into the revised manuscript "To further examine the structural basis of this selectivity, we compared DNO@Dox with two related control constructs, a scaffold-free 3apt-tFNA@Dox and a non-cholesterol 0chol-DNO@Dox (Figure S25). In comparison, only cholesterol-anchored DNO@Dox showed clear cell selectivity, with uptake mainly observed in cells expressing all three target receptors." (**Page 23, Lines 397–401**).

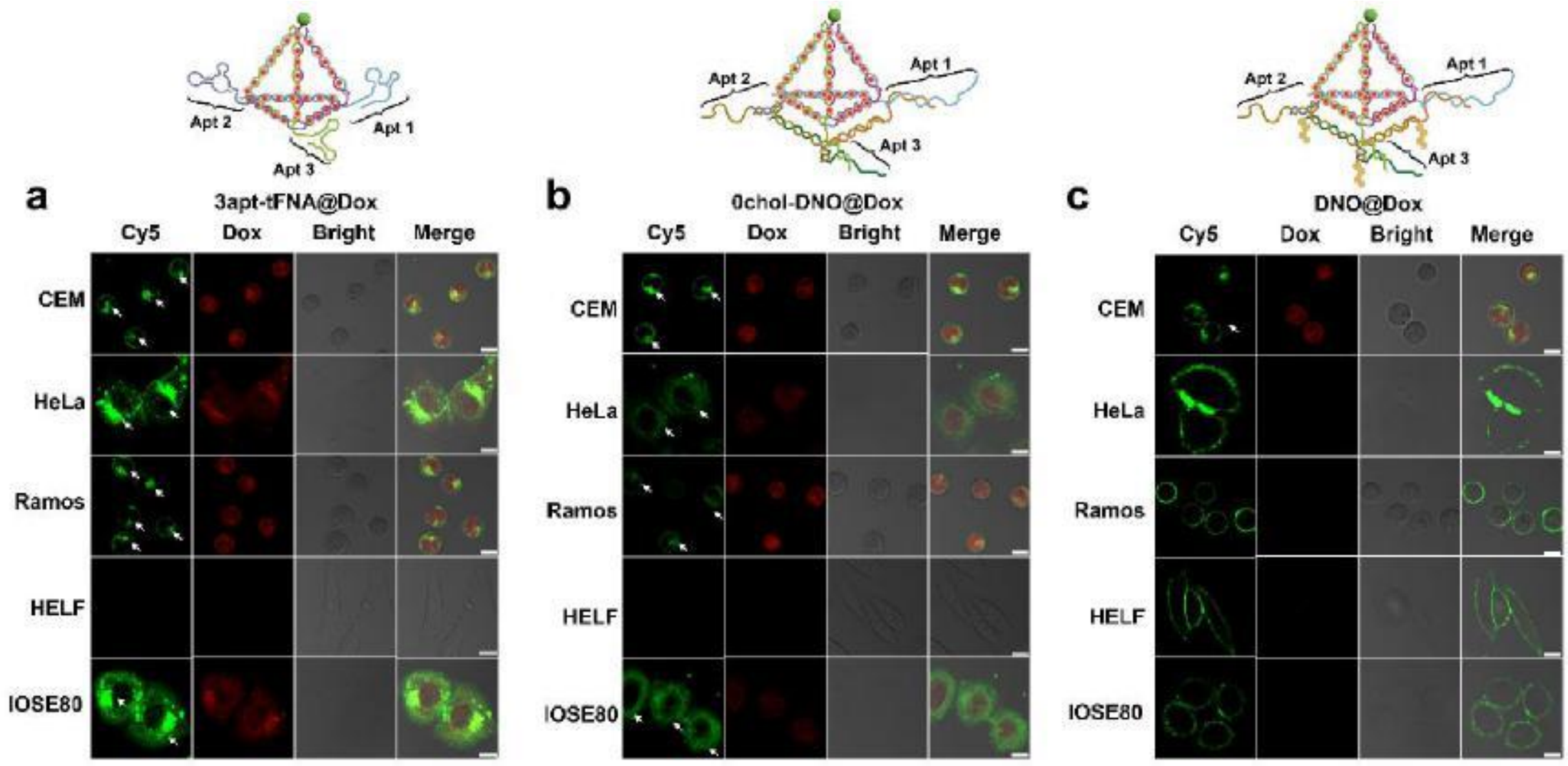

Figure S25. Comparative study of the cellular localization and uptake of DNO@Dox and related control constructs in different cell lines. **(a)** 3apt-tFNA@Dox, **(b)** 0chol-DNO@Dox, and **(c)** DNO@Dox. Confocal images were taken after 2 h incubation at 37 °C. Cy5 tracks the nanostructure, while intrinsic Dox fluorescence monitors drug delivery. Scale bars, 10 μm.

4. The electrophoretic mobility shift in Figure 1b (lane 8) is insufficient to distinguish the DNA nano-octopus from the standard tetrahedron. The resolution of AFM and TEM imaging is inadequate. Given the potential for dehydration artifacts in dry-state imaging, Cryo-EM should be provided to accurately confirm the dimensions and integrity of the nanostructures in solution.

Response: We thank the reviewer for this observation. To unambiguously resolve the electrophoretic mobility difference between the standard tetrahedron (tFNA) and the fully assembled DNA nano-octopus (DNO), we have optimized the electrophoretic conditions by increasing the agarose gel concentration from 2% to 3%. As shown in the revised Figure 1b, this adjustment now clearly resolves the mobility shift between the tFNA control (Lane 4) and the assembled DNO (Lane 8). The slower migration of DNO relative to tFNA is consistent with its larger molecular mass and more extended three-dimensional conformation, confirming successful

assembly of the integrated nanostructure.

Atomic force microscopy (AFM) is widely recognized as a powerful tool for characterizing DNA nanostructures. We acknowledge that the dry-state AFM images in the original submission were limited in resolution and may have been affected by structural deformation due to dehydration-induced collapse, a known artifact when three-dimensional DNA nanostructures are imaged in air. To address this concern, we have re-performed AFM imaging in liquid mode and replaced the original AFM panels in Figures 1c–e with the new liquid-AFM data. Together, the revised images provide a significantly more accurate morphological assessment of the nanostructures in their fully hydrated state, as AFM under native liquid conditions preserves the three-dimensional architecture of DNA nanostructures and eliminates dehydration artifacts.

We have also performed cryo-electron microscopy (Cryo-EM) on all three nanostructure components and replaced the original low-resolution TEM data with the new Cryo-EM micrographs (revised Figure S6). We note that high-resolution 3D reconstruction was not feasible for these constructs. This is likely due to the intrinsic technical limitations of Cryo-EM, a technique predominantly developed for protein characterization but less established for relatively small DNA nanostructures, including low electron-scattering contrast and possible structural flexibility or heterogeneity, which together hinder the acquisition of high-resolution structural information. (Nat. Biotechnol. 2021, 39, 378-386; Nano Lett. 2009, 9, 2747-2750).

Critically, our liquid-mode AFM imaging (revised Figures 1c–e) provides well-resolved, artifact-free morphological characterization in a fully hydrated environment, a method widely recognized as highly suitable for DNA nanostructure validation. Taken together, the orthogonal agreement among liquid AFM, Cryo-EM micrographs, gel electrophoresis, and theoretical modeling provides robust, multi-technique confirmation of correct nanostructure assembly and structural integrity in solution.

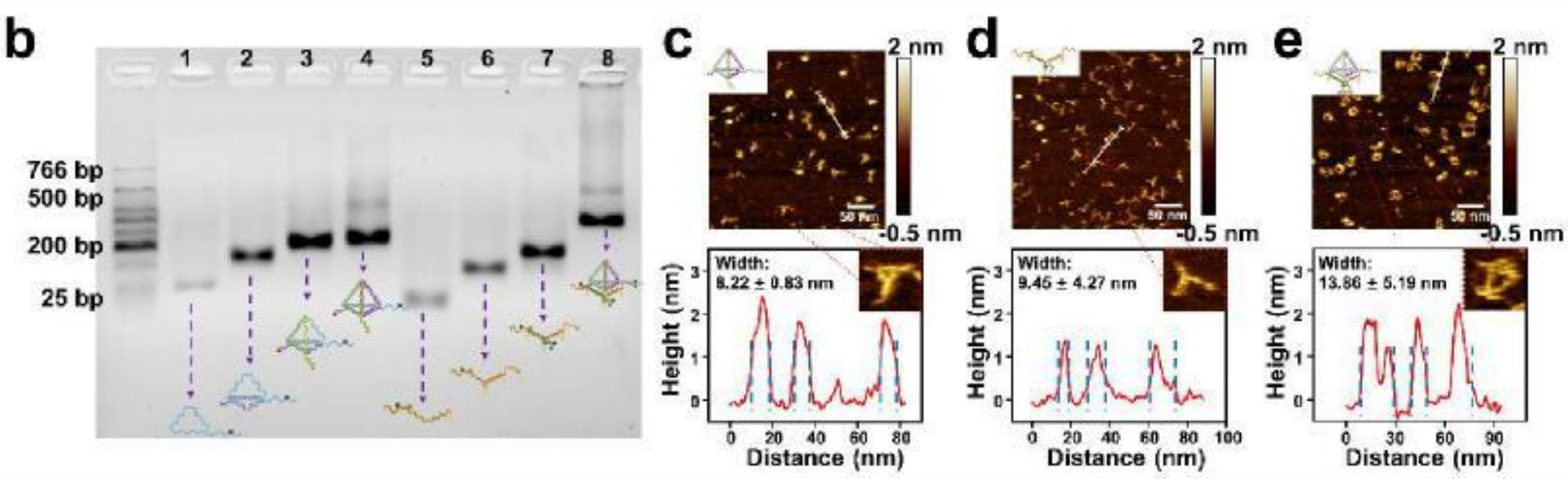

Figure 1. (b) Agarose gel electrophoresis verifying the stepwise assembly of tFNA (Lanes 1 to 4), Chol-MAS (Lanes 5 to 7) and the assembled DNO (Lane 8). **(c–e)** AFM images and height profiles of tFNA (c), Chol-MAS (d), and DNO (e). Scale bars, 50 nm.

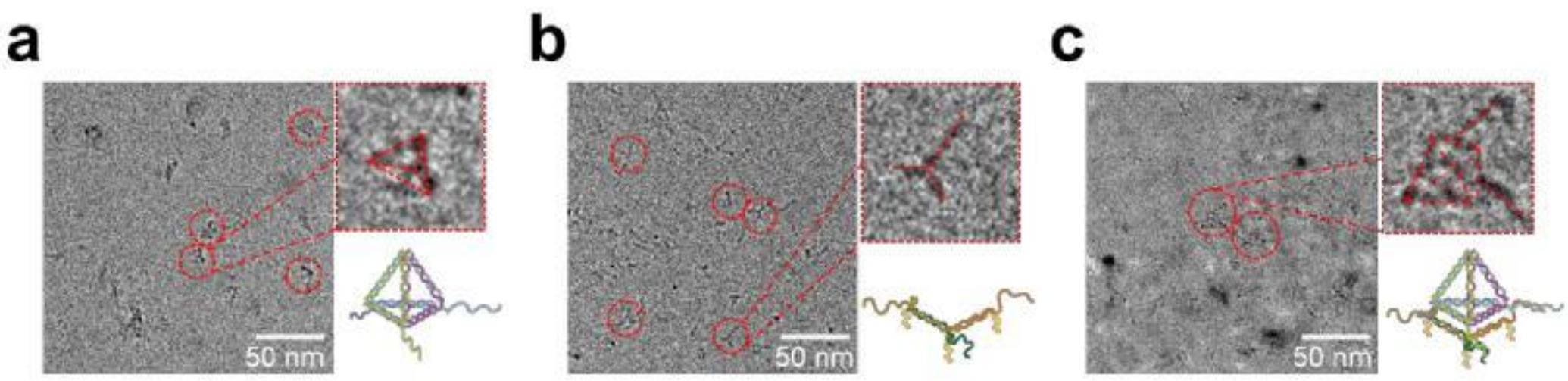

Figure S6. Representative Cryo-EM micrographs of DNO and its structural modules. **(a)** tFNA, **(b)** Chol-MAS, and **(c)** DNO in vitreous ice. Insets show enlarged views of representative particles.

Scale bars, 50 nm.

5. What is the specific functional necessity of the cholesterol anchor? Please provide data showing the system's performance in the absence of cholesterol to clarify its role in cell-surface localization.

Response: We thank the reviewer for this important question, which prompted us to rigorously dissect the functional contributions of the cholesterol anchor independent of the Chol-MAS scaffold. To address this, we synthesized an additional control construct—designated 0chol-DNO@Dox—which retains the complete Y-shaped scaffold and the three aptamer-lock duplex arms but lacks cholesterol modification (new Supplementary Figure S25b). In non-target Ramos and HeLa cells, 0chol-DNO@Dox still displayed appreciable intracellular Cy5 and Dox signals, indicating that aptamer-mediated membrane interactions remain sufficient to trigger some degree of internalization even in the absence of cholesterol anchoring. In stark contrast, DNO@Dox exhibited intracellular fluorescence and cytotoxic activity exclusively in CEM cells that co-express all three target receptors, with negligible uptake or toxicity in non-target cells. This functional distinction directly establishes the specific necessity of the cholesterol anchor. This cholesterol modification strategy provides stable, multivalent membrane anchoring that physically confines the intact DNO to the cell surface, preventing premature endocytosis of the nanostructure before AND-gate computation is complete, as previously documented for cholesterol-modified DNA (J. Am. Chem. Soc. 2019, 141, 18013-18020; Anal. Chem. 2020, 92, 9273-9280; J. Am. Chem. Soc. 2025, 147, 9800-9809). This membrane-resident state is critical for enabling the parallel, multi-input recognition process to occur. These new control data have been incorporated into the revised manuscript: "To further examine the structural basis of this selectivity, we compared DNO@Dox with two related control constructs, a scaffold-free 3apt-tFNA@Dox and a non-cholesterol 0chol-DNO@Dox (Figure S25). In comparison, only cholesterol-anchored DNO@Dox showed clear cell selectivity, with uptake mainly observed in cells expressing all three target receptors." (**Page 23, Lines 397–401**).

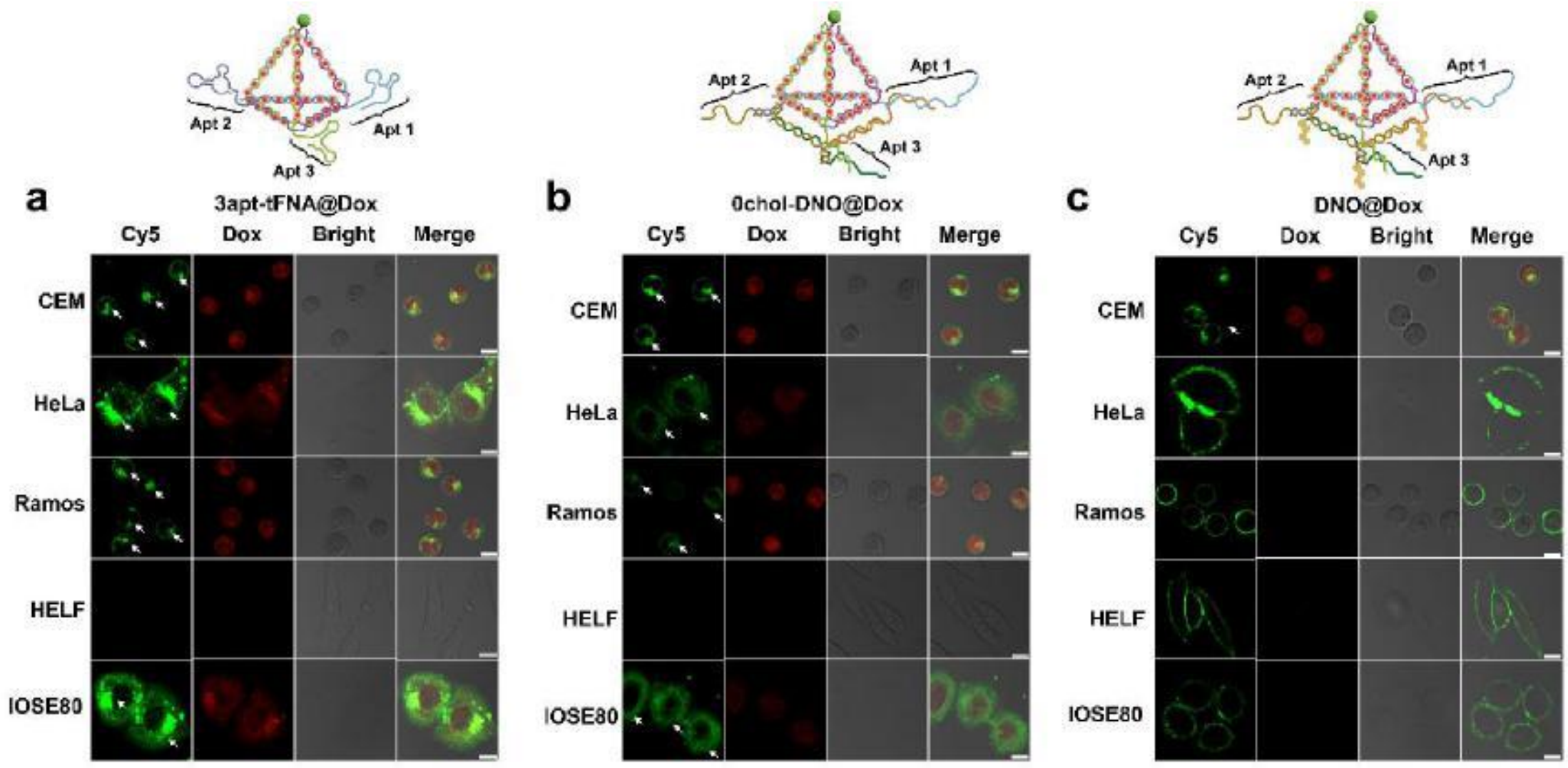

Figure S25. Comparative study of the cellular localization and uptake of DNO@Dox and related control constructs in different cell lines. **(a)** 3apt-tFNA@Dox, **(b)** 0chol-DNO@Dox, and **(c)** DNO@Dox. Confocal images were taken after 2 h incubation at 37 °C. Cy5 tracks the nanostructure, while intrinsic Dox fluorescence monitors drug delivery. Scale bars, 10 μm.

6. In Figure 1i, the initial fluorescence intensities across the three images are inconsistent. This discrepancy makes it difficult to quantitatively compare the samples before and after bleaching or to assess the recovery rate at 140s.

Response: We sincerely appreciate the reviewer's careful observation. The inconsistent fluorescence intensities across the three images were primarily due to continuous image recording during the experiment combined with the use of an excessively high excitation laser power, which led to severe photobleaching.

To resolve this issue and align the image presentation with our rigorous quantitative analysis, we reduced the laser intensity to minimize background quenching, re-performed the FRAP experiment, and replaced the images in the revised Figure 1i with these newly acquired images (Before bleaching, Bleaching at 5 s, and Recovery at 155 s). Although optimizing the laser power significantly improved visual consistency, a minor degree of background photobleaching is inherently unavoidable during continuous confocal scanning of live cells, especially since antifading agents cannot be used in live-cell imaging. Therefore, the normalization procedure remains indispensable. Accordingly, we have corrected the y-axis label of Figure 1j to "Normalized intensity (%)" to accurately reflect the data processing method, and have detailed this normalization procedure in the revised figure legend. We thank the reviewer for their rigorous scrutiny, which has greatly helped us improve the accuracy of our data presentation.

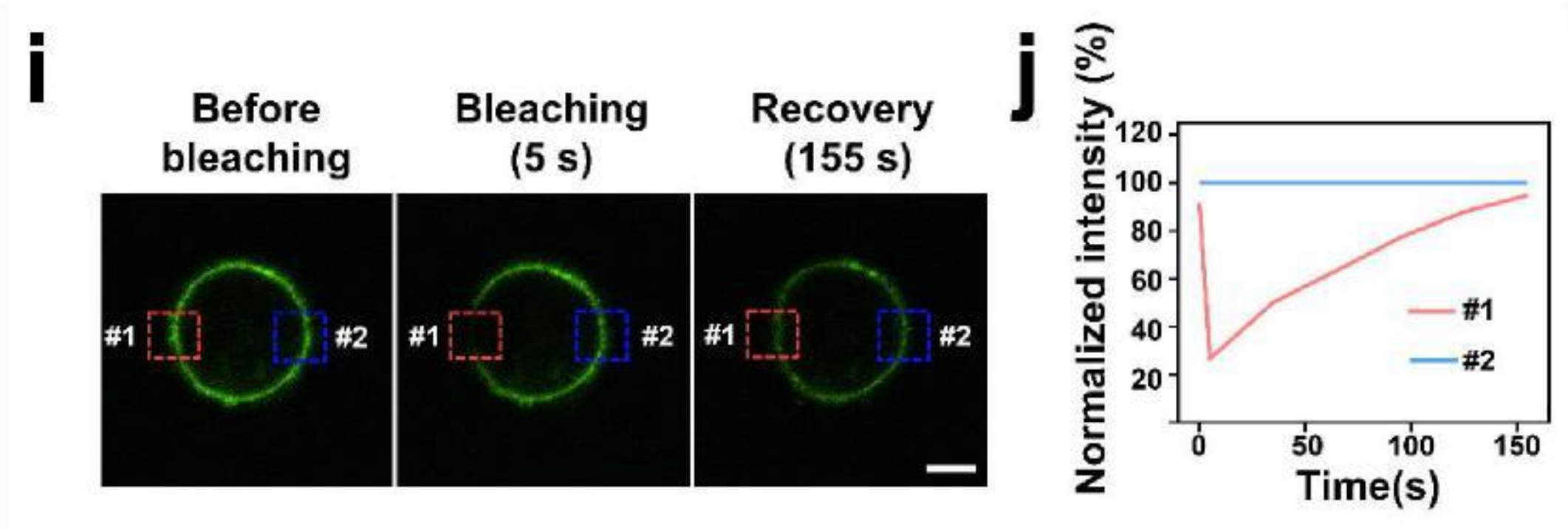

Figure 1. (i) Representative CLSM images acquired fluorescence before bleaching, immediately after bleaching (5 s), and during recovery (155 s). Region #1 indicates the bleached area, whereas Region #2 serves as the control. Scale bars, 5 μm. **(j)** Normalized fluorescence recovery curves for regions #1 (red) and #2 (blue), indicating lateral diffusion of the DNO and dynamic membrane anchoring.

7. In Figure 2c, the DNA band in lane 1 is significantly diffused. Please provide an explanation for this lack of stability or poor resolution.

Response: We thank the reviewer for this careful observation. Lane 1 in the Figure 2c corresponds to the tFNA control, which in this experiment carries terminal fluorescent labels (Cy3 and Cy5) for fluorescence readout. We agree that the band appears more diffuse than expected. This diffuse appearance does not necessarily indicate intrinsic instability of the tFNA structure. Instead, we hypothesize that it may result from band broadening associated with the fluorescently labeled DNA in this assay. Specifically, cyanine dyes contain extended ring structures that could potentially introduce additional friction or weak non-covalent interactions within the polyacrylamide gel matrix

during electrophoresis. Such interactions can lead to migration heterogeneity and subsequent band smearing. This interpretation is supported by previous literature (Nucleic Acids Res. 1998, 26, 2797–2802).

In addition, in our own unlabeled gel controls (Figure 1b and Figure S10), the corresponding DNA bands are clear and well-resolved without obvious diffusion, which argues against intrinsic instability of the DNA nanostructure under the gel conditions used.

To address this concern, we have added the following sentence to the revised manuscript:" Despite the slight smearing, which likely arose from labeling-related electrophoretic broadening rather than intrinsic instability (as indicated by the clear migration of unlabeled tFNA in Figure 1b and Figure S10), fluorescence imaging afforded a direct visual readout of this logic (Figure 2c)." (**Page 12, Lines 234–236**).

8. In Figure 3b, the Cy5 signal is disproportionately strong and appears to extend beyond the cell membrane boundaries. Is this an artifact of signal saturation, or does it indicate a leakage problem?

Response: We thank the reviewer for this careful observation. We agree that the Cy5 signal in the original Figure 3b appeared overly strong and may have visually extended slightly beyond the cell membrane boundary. We believe that this effect is more likely attributable to the confocal acquisition and display settings, such as relatively high gain or display intensity in the Cy5 channel, which may have caused slight signal saturation and optical spreading, rather than probe leakage. This conclusion is consistent with our other experimental results. First, the colocalization and line-scan analysis with the membrane dye DiI showed that DNO was mainly localized on the cell membrane. Second, extracellular DNase I treatment markedly abolished the membrane-associated fluorescence signal, further indicating that the nanostructure was exposed on the outer cell surface. Collectively, these results do not support a leakage problem.

To avoid possible misunderstanding arising from the original image presentation and to further improve image quality, we reacquired the corresponding confocal images under more appropriate Cy5 channel settings and replaced the original Figure 3b with the updated image. This revision only improves the quality of image presentation and does not affect the original results or conclusions.

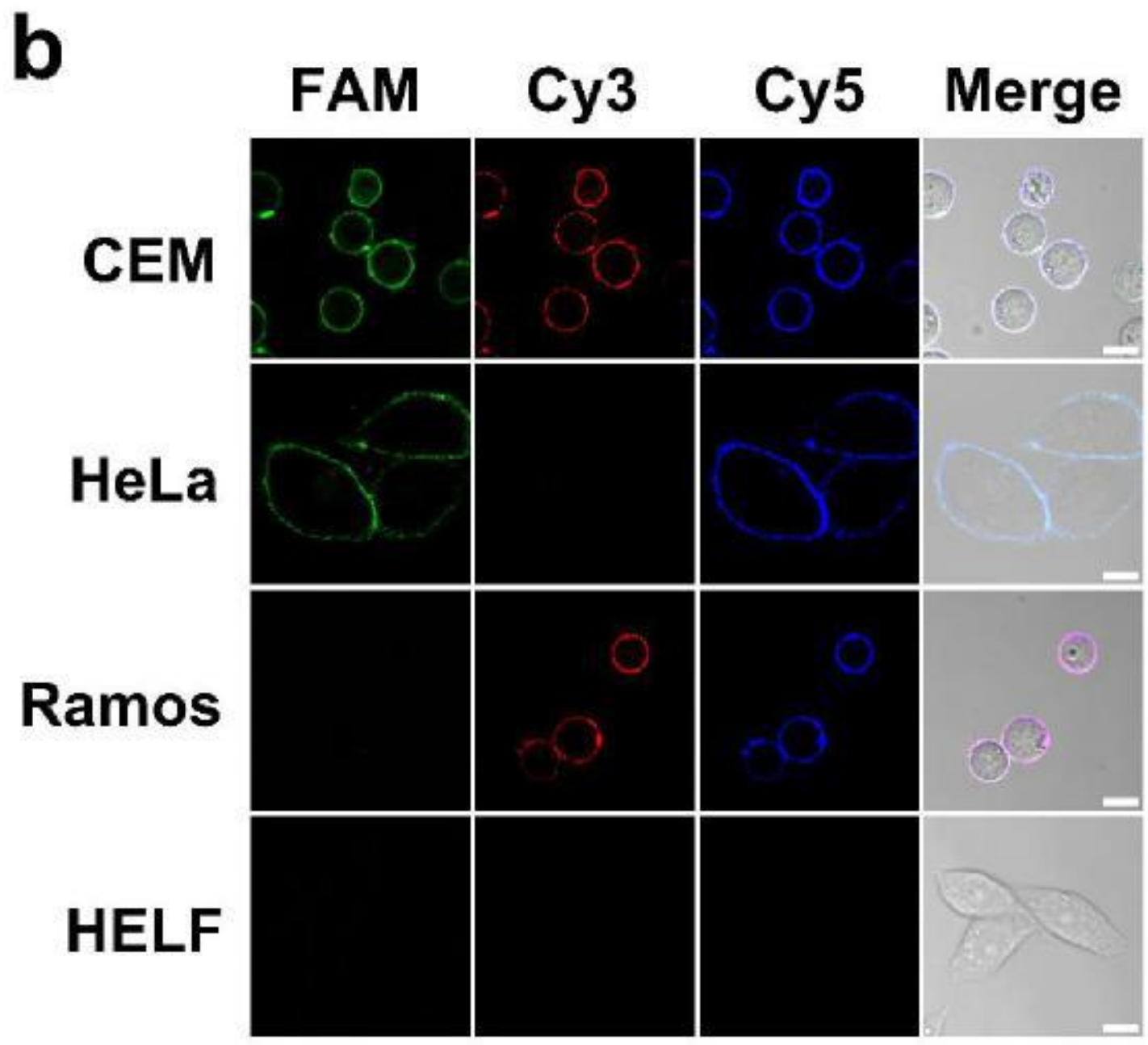

Figure 3. (b) CLSM images showing different fluorescence signals after incubating four cell types with DNO for 20 min. Scale bar, 10 μm.

9. In Figure 3d, the authors should monitor the same field of view/cell population to track the release and uptake process over time. Comparing different spots at different time points makes the data less convincing.

Response: Thank you for this insightful suggestion. We fully agree that tracking the same field of view over time would provide more convincing evidence for the release and uptake process. Accordingly, we repeated the CLSM experiment and supplemented the revised manuscript with images acquired from the same field of view at different time points.

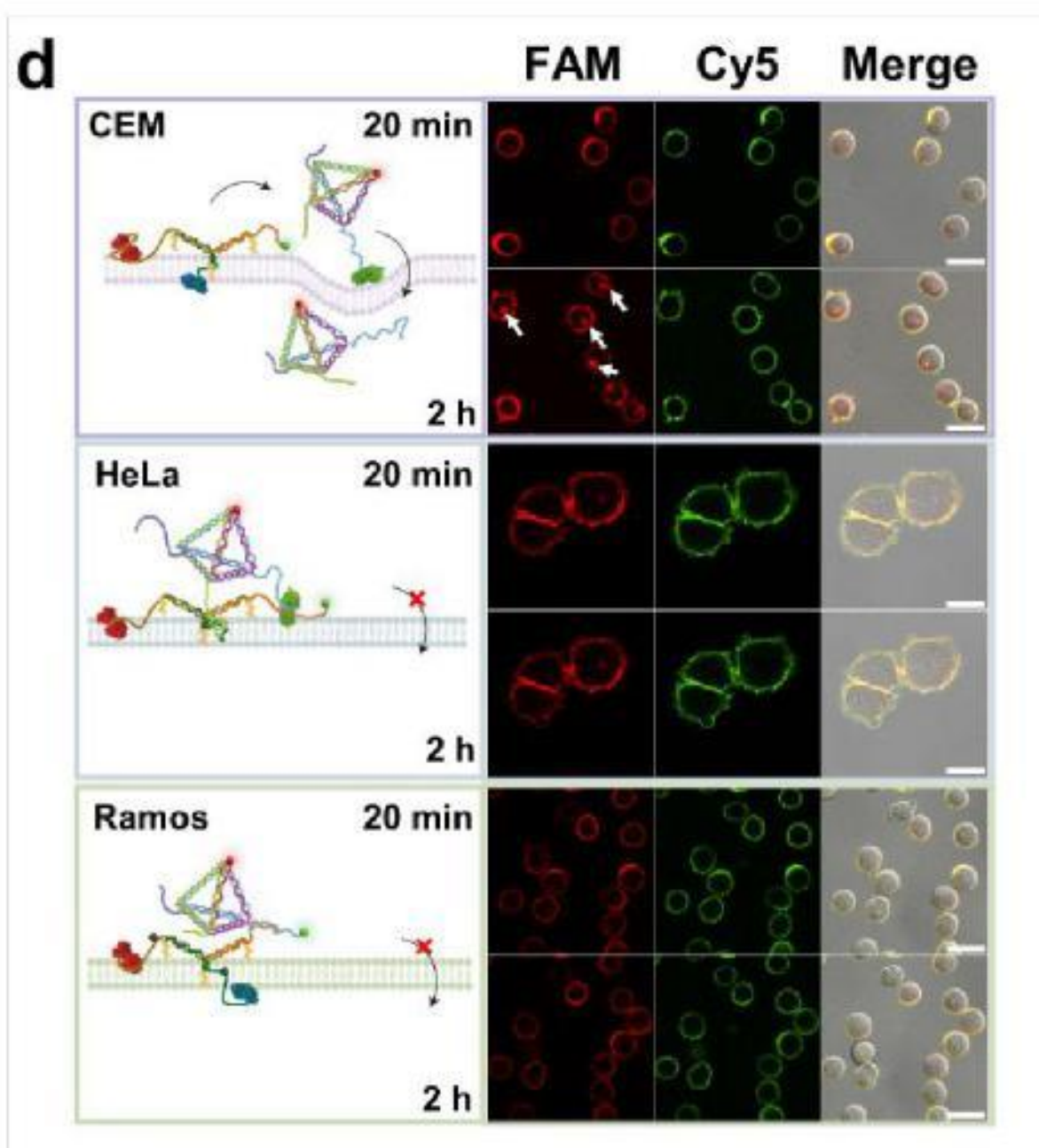

Figure 3. (d) Schematic illustration and CLSM images of the cellular distribution of Cy5-labeled

tFNA and FAM-labeled Chol-MAS in different cell types. Cells were incubated with the constructs for 20 min or 2 h before imaging. Scale bar, 20 μm.

10. Several figures mentioned in the results (Figures S11e–f and 5c–f) appear to be missing from the provided main text or supplementary sections.

Response: Thank you for this careful comment. We apologize for the lack of clarity in the original manuscript. We have now added concise explanations in the relevant sections, indicating that original Figures S11e–f (now Figures S15e–f) show the fluorescence spectra of free Dox standards and the corresponding calibration curve for Dox loading quantification (**Page 22, Lines 370–371**), whereas **Figures 5c–f** present the quantitative apoptosis analysis (Annexin V–FITC/PI) for CEM, HeLa, Ramos, and HELF cells (**Page 22, Lines 389–390**).

11. The statement on page 21 (line 382) regarding apoptosis is not supported by Figure 5c, as the difference between DNO and DNO@Dox appears negligible. Rigorous statistical analysis must be performed and indicated for all data in Figures 5c–f.

Response: Thank you very much for your careful reading and for pointing out this inconsistency. After carefully checking the original data and figure preparation files, we confirmed that while the raw flow cytometry data in Figure 5b were correct, the statistical analysis presented in **Figure 5c** in the original manuscript contained errors. We have now re-analyzed the apoptosis data, updated the figures, and revised the accompanying text. Thank you again for helping us identify and correct this issue.

12. The claim of "dramatic tumor regression" (page 24) is not supported by Figure 6c. Additionally, there is a significant discrepancy between the efficacy of peritumoral treatment versus intravenous administration that needs addressing.

Response: Thank you for this important comment. We appreciate your careful evaluation of the in vivo efficacy data and agree that the wording "dramatic tumor regression" was not sufficiently precise in light of the data shown in Figure 6c. We therefore revised the corresponding text in the manuscript and replaced "dramatic tumor regression" with the more accurate and cautious description "tumor growth inhibition" (**Page 25, Line 424**).

The enhanced efficacy observed with peritumoral administration compared with intravenous administration is expected and reflects well-documented differences in delivery efficiency between local and systemic routes. Peritumoral injection bypasses the well-established systemic delivery barriers including opsonization, mononuclear phagocyte system clearance, and renal filtration, all of which reduce nanoparticle tumor accumulation to a median of only a very small fraction of the injected dose following intravenous administration (Nat. Rev. Mater. 2016, 1, 16014). This route therefore establishes the intrinsic therapeutic potential of the DNO@Dox platform when delivery constraints are removed. Although intravenous administration yielded quantitatively lower tumor inhibition, this clinically relevant route demonstrated that the AND-gate logic and aptamer-mediated targeting remain functionally effective under systemic conditions, as DNO@Dox still achieved significant tumor growth inhibition. Such differential efficacy between local and systemic administration is well-precedented in the nanomedicine literature (Signal Transduct Target. Ther. 2023, 8, 293; Int. J. Nanomedicine 2018, 13, 7409–7426; J. Am. Chem. Soc. 2020, 142, 1265–

1277). Accordingly, both routes provide complementary and essential validation. We have also added a discussion of the difference between peritumoral and intravenous administration in the revised manuscript "The weaker tumor inhibition observed after intravenous administration relative to peritumoral treatment likely reflects the additional delivery barriers encountered under systemic administration before the nanostructures reach the tumor site. Nevertheless, DNO@Dox still retained significant anti-tumor activity after intravenous administration." (**Page 26, Lines 447–450**).

13. The data in Figures S20–S21 appear redundant, as similar experiments involving tail vein injections are already presented in Figure 6 and Figures S22–S23.

Response: We thank the reviewer for this comment. We respectfully clarify that original Figures S20–S21 (now Figures S26–S27; peritumoral administration) and original Figure 6/Figures S22–S23 (now Figure 6/Figures S28–S31; intravenous administration) are not redundant but represent distinct and complementary experimental arms. As noted in our response to the preceding comment, peritumoral administration bypasses systemic delivery barriers to establish the intrinsic therapeutic potential of the DNO@Dox platform, whereas intravenous administration tests its translational relevance under clinically realistic conditions. Both routes provide essential and independent validation. Previous studies have also examined local and intravenous administration in parallel, consistent with our experimental design (Signal Transduct. Target. Ther. 2023, 8, 293; Int. J. Nanomedicine 2018, 13, 7409–7426; J. Am. Chem. Soc. 2020, 142, 1265–1277).

14. Please correct the erroneous labels in Figure 6e.

Response: Thank you for pointing this out. We apologize for the labeling error in Figure 6e. The erroneous labels in Figure 6e have been corrected in the revised manuscript. Thank you again for your careful reading.

Response to Reviewer 2:

This manuscript reports a membrane-anchored biomimetic DNA nano-octopus featuring multiple aptamer arms that are described as allosterically regulated for parallel molecular probing and on-site drug release. The overall concept is creative and timely, integrating DNA nanotechnology with membrane-localized sensing and responsive therapeutic delivery. The work has potential interest for the broad readership of Nature Communications. However, several key aspects of the mechanistic basis, quantitative performance, and biological relevance are not yet sufficiently substantiated. Several points would benefit from clarification and additional quantitative support to further strengthen the manuscript.

Response: Thank you very much for your thoughtful and encouraging overall assessment of our work, as well as for your constructive comments. In response to your comments, we have carefully revised the manuscript and Supplementary Information to clarify the relevant issues and provide additional supporting data where possible. Detailed point-by-point responses are provided below.

1. The manuscript refers to the aptamer arms as allosterically regulated. While the presented data are consistent with target-induced functional responses, additional clarification of the underlying allosteric mechanism would improve mechanistic rigor. A clearer definition and, if possible,

supporting structural or spectroscopic evidence would strengthen this central claim.

Response: We thank the reviewer for this valuable comment. We agree that the term "allosterically regulated" should be clarified more carefully and described with more cautious wording. To provide a clearer definition, we first refer to similar design concepts established in previous studies. For example, earlier work has utilized aptamer (Sgc8c) recognition of membrane receptors (PTK7) to induce strand displacement and convert biological inputs into downstream DNA signals for cell-surface molecular computation (Nat. Commun. 2025, 16, 923). This precedent provides literature-level support for the target-responsive mechanistic interpretation proposed in our study. We then clarify the definition of "allosteric" in this context: "Specifically, 'allosteric' refers to the conformational switching behavior of the aptamer arms, wherein target binding to the aptamer recognition domain induces a structural rearrangement that displaces the complementary lock strand, thereby destabilizing the bridging duplex." (**Page 5, Lines 115–118**).

In addition, we performed exploratory molecular modeling to analyze the conformational state of the aptamer arm before and after target binding from a structural perspective. Taking the Sgc8c aptamer and its target PTK7 protein as a representative example, we constructed theoretical models of the DNO architecture both with and without PTK7 binding using literature-guided HDOCK docking (Proc. Natl. Acad. Sci. U.S.A. 2024, 121, e2404060121), structural placement onto the tFNA module, and short-timescale relaxation with GROMACS. The model of the assembled DNO structure containing the Sgc8c module is shown in Figure S2a, while the PTK7-bound model is shown in Figure S2b. The orange region indicates the PTK7 Ig3–Ig4 domain. Comparison of the PTK7-free and PTK7-bound models supports a structurally plausible allosteric rearrangement: upon receptor protein binding, the aptamer undergoes a conformational switch from an extended, partially unfolded "locked" state to a compact, folded structure, which displaces the complementary lock strand and destabilizes the corresponding bridging duplex arm.

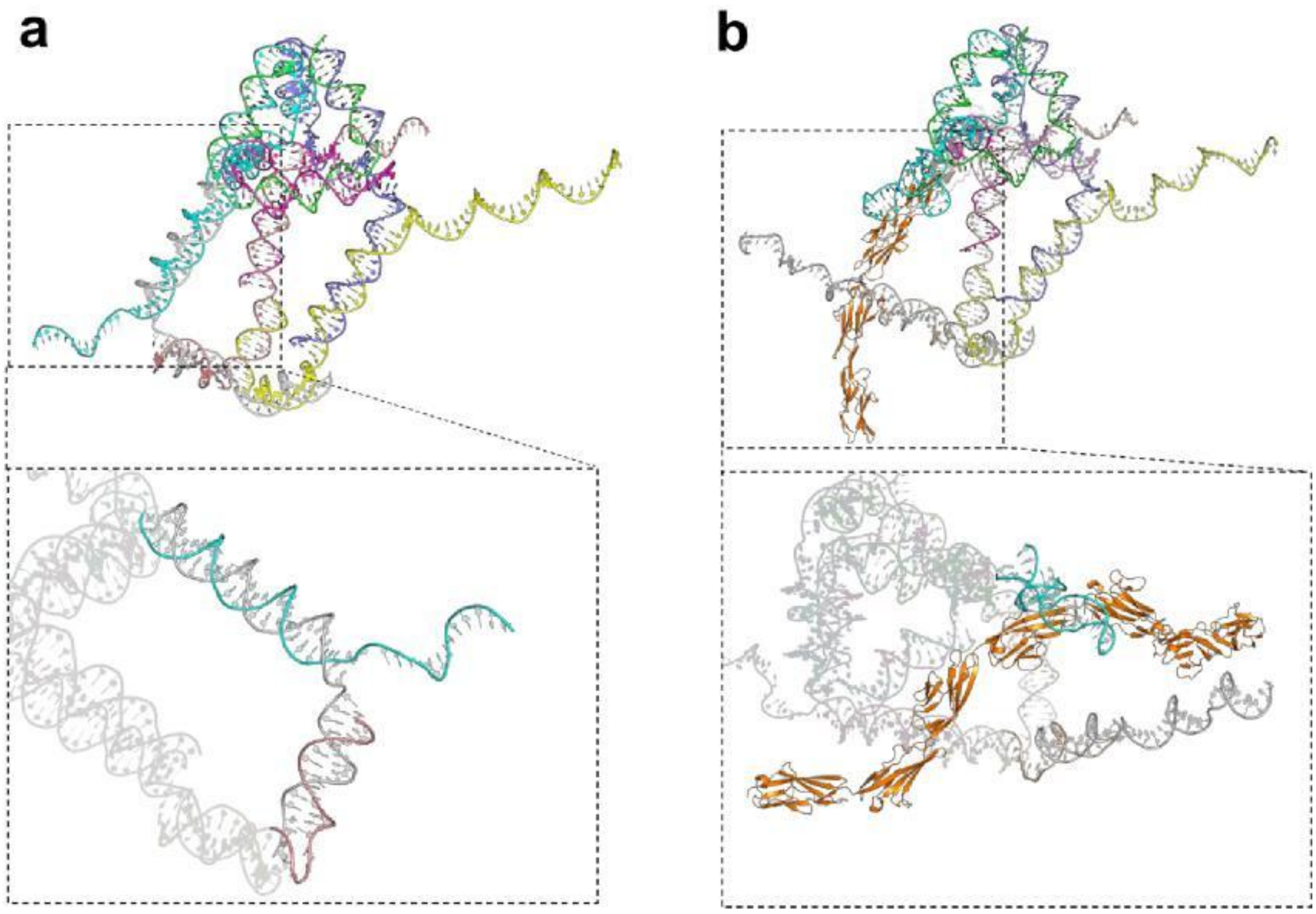

Figure S2. Exploratory structural models of the DNO before and after PTK7 docking. **(a)**

Theoretical pre-binding model of the assembled DNO containing the Sgc8c module. **(b)** Theoretical post-docking model of the DNO–PTK7 complex. The orange structure indicates the docked PTK7 Ig3–Ig4 domain. Insets show enlarged local views of the Sgc8c-containing region before and after PTK7 docking.

2. The manuscript highlights parallel probing as a key advantage of the nano-octopus architecture. However, the potential for signal interference among different aptamer arms is not systematically evaluated. The authors are encouraged to provide additional evaluations under multi-target conditions, to further support this claim.

Response: We thank the reviewer for this insightful comment. We agree that potential signal interference among the three aptamer arms should be addressed more explicitly when highlighting parallel probing as a key feature of the DNO platform. Although this point was not explicitly discussed in the original manuscript, the relevant experimental evidence is already included in the current study. In the in vitro multi-input fluorescence analysis (Figures 2d–f), each fluorescence channel mainly responds to its designated input, with no obvious non-corresponding activation or significant cross-talk. At the receptor-recognition level, the competitive binding assay in Figure S11 shows that the three aptamers bind to independent and non-competing membrane receptors. Consistently, in the fully assembled DNO system, the selective blocking experiment in Figure S1 3 shows that pre-blocking one target selectively suppresses only the corresponding fluorescence channel, while the other two channels remain largely unchanged. At the cellular level, the multichannel fluorescence patterns across different cell lines in Figures 3b and 3c, together with the mixed-cell experiment in Figure 4, further support that this three-arm system retains good channel discrimination in a heterogeneous environment. Taken together, these results support minimal observable inter-arm interference under multi-input and multi-target conditions.

3. As the sensor system showed excellent performance, I wonder if the authors could try to provide some quantitative sensing parameters (e.g., detection limits, linear response ranges, and reproducibility).

Response: We thank the reviewer for this valuable suggestion. To address this, we performed a quantitative analysis of CEM cell (target cell) detection in mouse blood-cell background, and the corresponding results are now summarized in new Supplementary Figure S14 and Table S2. Hoechst-prelabeled CEM cells were spiked into washed mouse blood-cell suspensions at predetermined levels, followed by incubation with Cy5/BHQ1-labeled DNO and flow cytometry analysis. The spiked CEM cell fractions served as the input conditions, and the quantitative output was recorded as the number of detected tumor cells per 10,000 analyzed events. As shown in Figure S14c, the detected tumor-cell count exhibited an overall linear relationship with the theoretical number of CEM cells across the tested range of 0–10,000 cells, corresponding to 0–100% of the analyzed events. To better present the low-abundance region, the detected tumor-cell numbers over the 0–100 cell range are shown separately in Figure S14d. Based on Figure S14d and Table S2, the lowest tested level that could be reliably quantified under the present assay conditions was 50 tumor cells within 10,000 analyzed events, with recovery values generally around 90%. We have added the corresponding description in the revised main text. (**Page 20, Lines 352–354**).

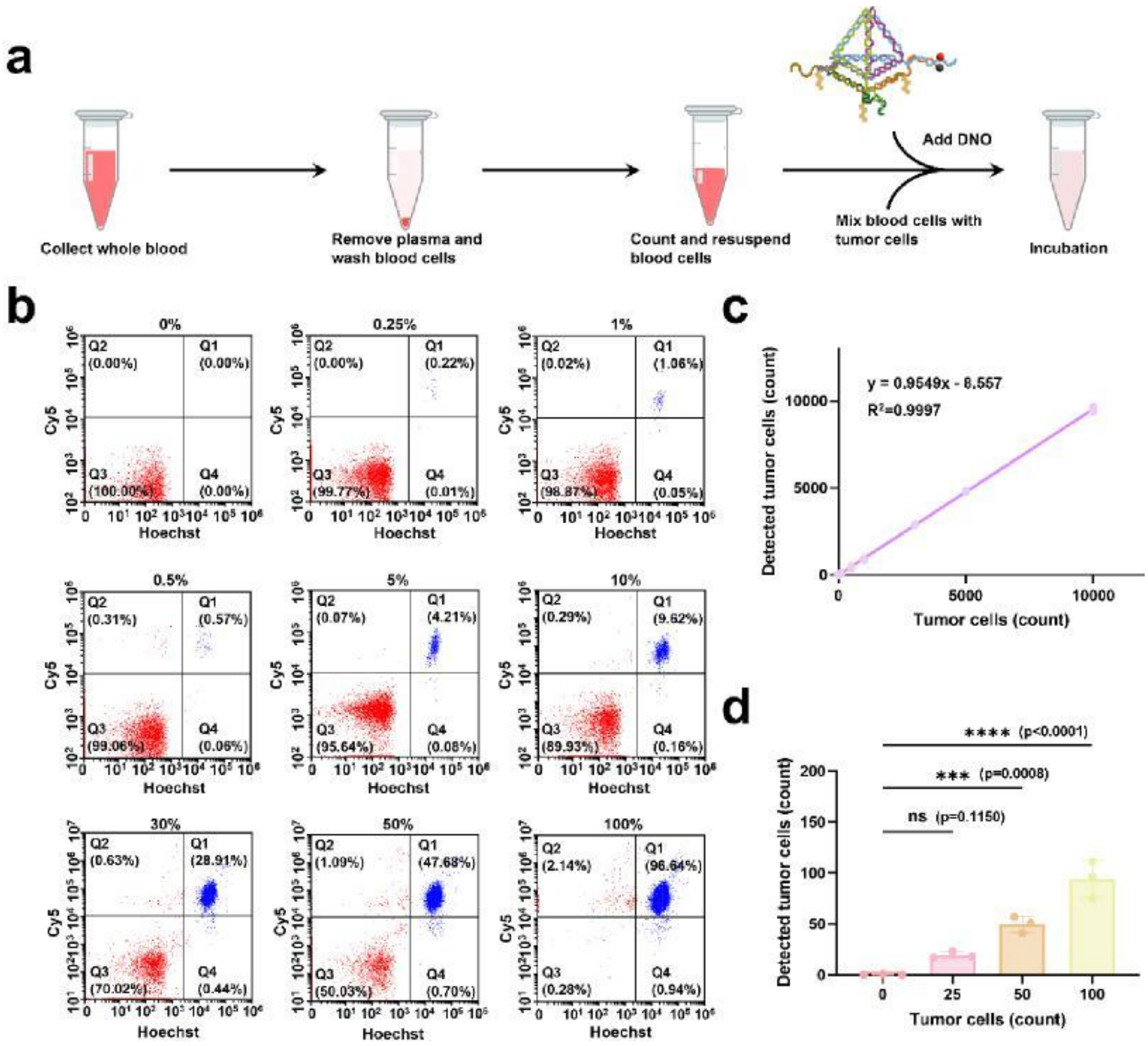

Figure S14. Quantitative analysis of CEM cell detection in a mouse blood-cell background. (a) Schematic illustration of the experimental workflow. Mouse whole blood was collected, followed by plasma removal, blood-cell washing, counting, and resuspension. Hoechst-prelabeled CEM cells were spiked into the washed mouse blood-cell suspension at predetermined levels, incubated with Cy5/BHQ1-labeled DNO for 30 min, washed, and analyzed by flow cytometry. A total of 10,000 events were collected for each sample. **(b)** Representative flow cytometry dot plots of Hoechst-prelabeled CEM cells spiked into washed mouse blood-cell suspensions at different theoretical levels. Hoechst-positive cells were detected in the PB450 channel, and DNO recognition was measured in the Cy5 channel. **(c)** Linear fitting of theoretical tumor-cell numbers and detected tumor-cell numbers over the 0–10,000 cell range, with a fitting equation of $y = 0.9549x - 8.557$ and $R^2 = 0.9997$. **(d)** Detected tumor-cell numbers at different theoretical input levels over the 0–100 cell range. Bars represent the mean ± SD, and dots indicate individual measurements. Statistical significance was determined by one-way ANOVA followed by Dunnett's multiple comparisons test, with the 0-cell group as the control. ns, $P > 0.05$; ***$P < 0.001$; ****$P < 0.0001$.

Table S2. Quantitative parameters for CEM cell detection in a mouse blood-cell background

Theoretical tumor cells	Nominal fraction (%)	Detected tumor cells[a]	Detected proportion (%)[a]	Recovery (%)[b]	RSD (%)[c]
0	0	0.33 ± 0.58	0.003 ± 0.006%	—	—
25	0.25	19.33 ± 3.21	0.193 ± 0.032%	77.33	16.63

50	0.50	49.67 ± 8.08	0.497 ± 0.081%	99.33	16.27
100	1.00	94.00 ± 18.08	0.940 ± 0.181%	94	19.24
500	5.00	426.33 ± 68.24	4.263 ± 0.682%	85.27	16.01
1000	10.00	918.00 ± 53.81	9.180 ± 0.538%	91.8	5.86
3000	30.00	2888.67 ± 3.21	28.887 ± 0.032%	96.29	0.11
5000	50.00	4791.67 ± 22.59	47.917 ± 0.226%	95.83	0.47
10000	100.00	9522.67 ± 132.99	95.227 ± 1.330%	95.23	1.4

[a] Data are presented as mean ± SD from three independent experiments.

[b] Recovery (%) = detected tumor cells / theoretical tumor cells × 100.

[c] RSD, relative standard deviation.

4. Some figures (e.g., Figure 1c–e and Figure 4c) would benefit from improved resolution and clearer annotations.

Response: We thank the reviewer for this careful observation. We have revised Figures 1c– e and Figure 4c accordingly. For Figures 1c–e, we acknowledge that the original AFM images were acquired under dry-state conditions, which can induce structural collapse of three-dimensional DNA nanostructures upon dehydration and thereby compromise both resolution and morphological accuracy. To eliminate these artifacts, we have re-characterized the relevant samples using liquid-mode AFM (tapping mode in aqueous buffer), which preserves the native hydrated conformation of the nanostructures. The original dry-state AFM panels have been replaced with the newly acquired liquid-mode AFM data in the revised figures. For Figure 4c, we have adopted a larger font size to achieve improved resolution.

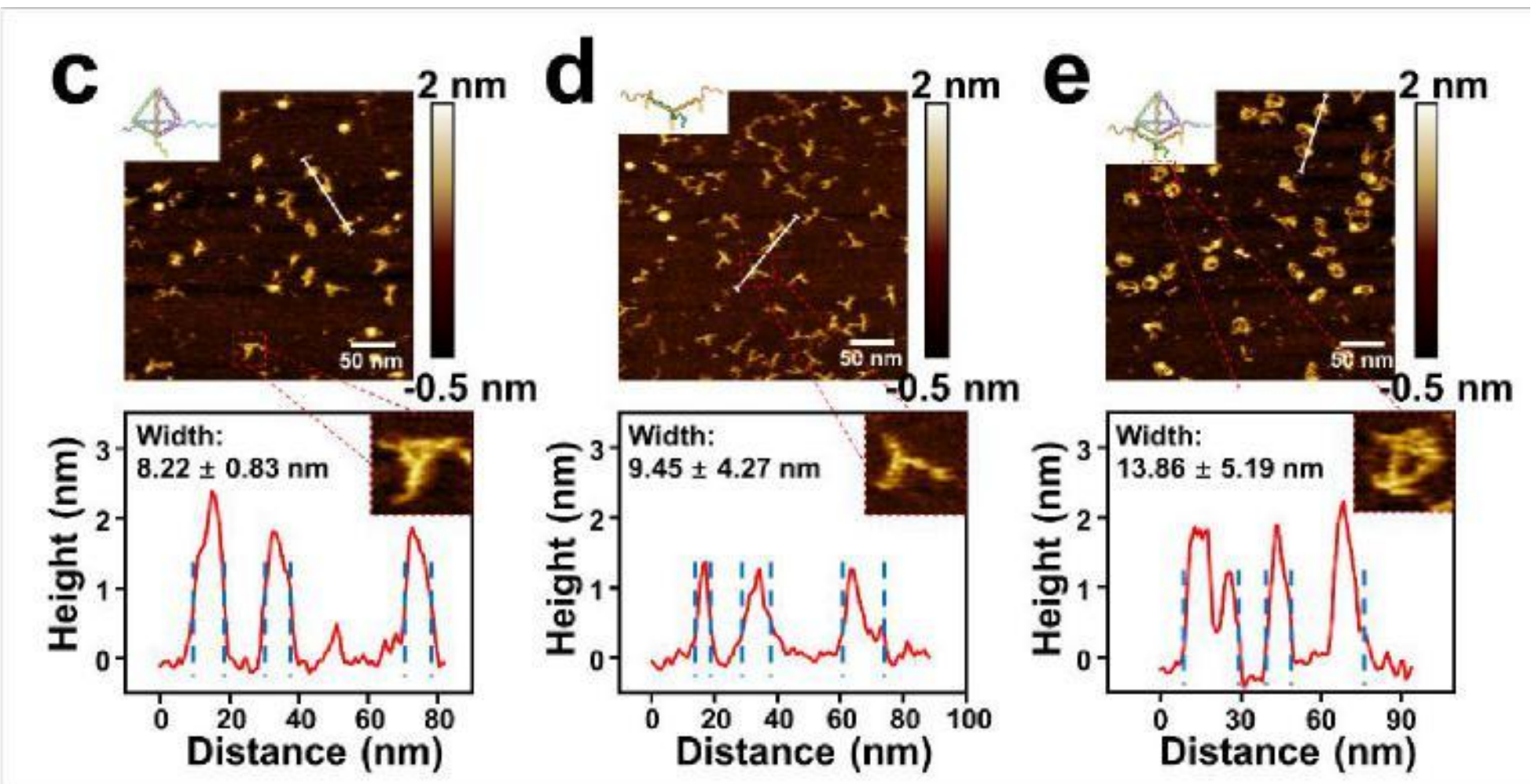

Figure 1. (c-e) Liquid-mode AFM images and corresponding cross-sectional height profiles of tFNA (c), Chol-MAS (d), and DNO (e). Scale bars, 50 nm.

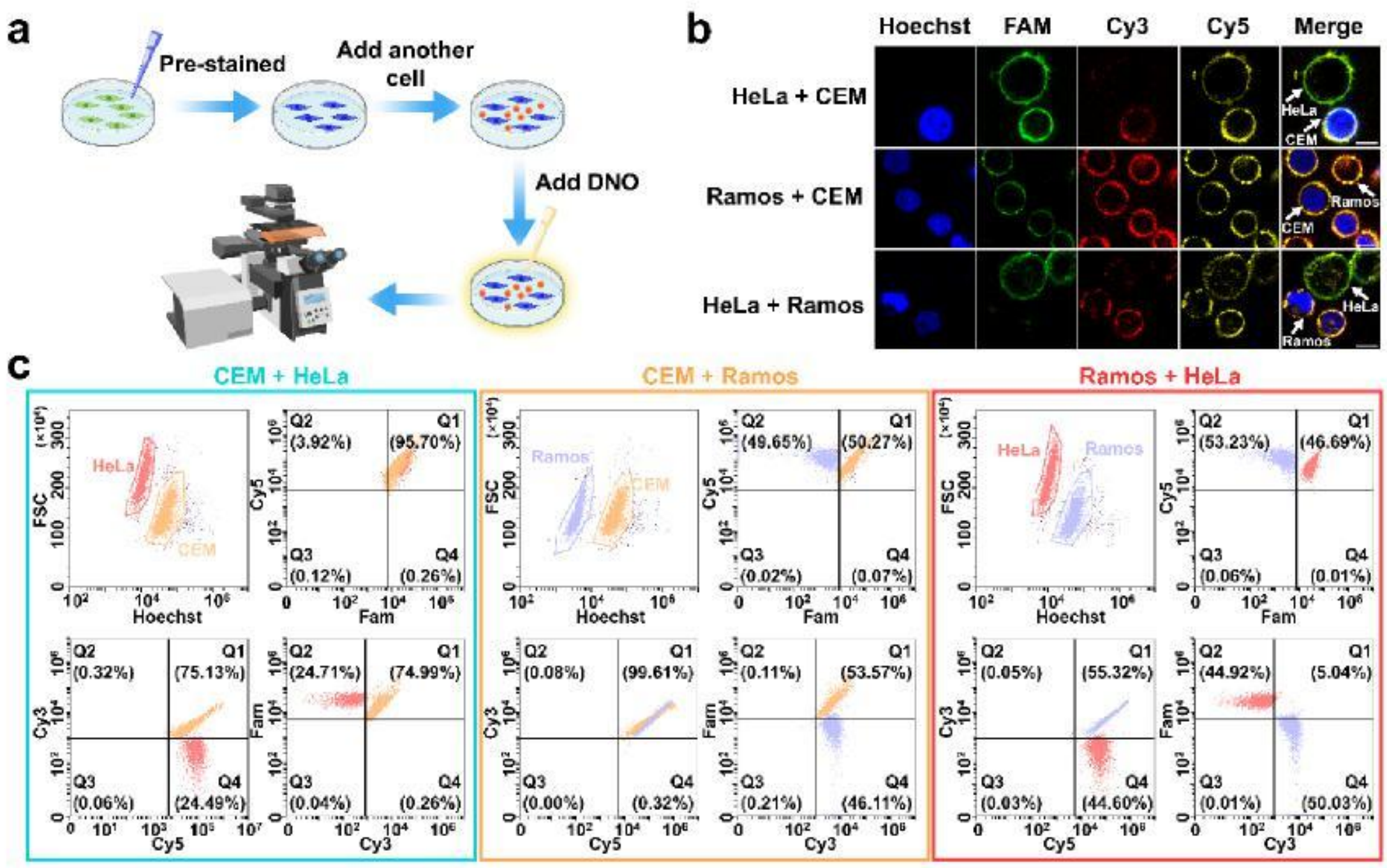

Figure 4. Performance of the DNO in complex cell mixtures. **(a)** Schematic of the confocal imaging assay. The nuclei of one cell type were prestained for identification and mixed with a second cell type, and the resulting cell mixtures were treated with the DNO and imaged by CLSM. **(b)** CLSM images and **(c)** flow cytometry of mixed cell populations incubated with the DNO at 37 °C for 20 min showed distinct fluorescence patterns, allowing for simultaneous identification of CEM, HeLa, and Ramos cells. Scale bar, 10 μm.

5. In Scheme 1, some labels are not consistently capitalized. Please unify the formatting to improve clarity and presentation consistency.

Response: Thank you for pointing this out. We have carefully checked the labels in **Scheme 1** and unified the capitalization throughout the figure in the revised manuscript to ensure consistency in formatting and presentation clarity.

6. To further support the proposed allosteric mechanism, theoretical modeling or molecular simulations (e.g., MD or coarse-grained simulations) could be highly valuable in elucidating the conformational coupling between target binding and functional responses of the aptamer arms.

Response: We thank the reviewer for this helpful comment. Because the molecular identities of the target proteins for Sgc4f and TCO1 remain uncharacterized, we focused our modeling efforts on the Sgc8c arm and its known target PTK7.

To address this point, we performed exploratory computational modeling in consultation with researchers specializing in molecular modeling to assess whether the Sgc8c arm remains structurally accessible in the assembled DNO and how its local conformation may change upon PTK7 engagement (Figure S2). For the PTK7-free DNO model, the initial DNO scaffold was generated using oxDNA according to the designed DNA sequences, base-pairing patterns, strand connectivity, and overall spatial arrangement, and was then converted into atomic coordinates and energy-minimized using GROMACS. For the PTK7-bound model, the reported NMR structure of the free Sgc8c aptamer (PDB ID: 8Y0F) was docked to the reported PTK7 binding region using HDOCK

(Proc. Natl. Acad. Sci. U.S.A. 2024, 121, e2404060121). The resulting Sgc8c–PTK7 complex was then positioned on the designated tFNA arm and subjected to structural relaxation with GROMACS. These exploratory models provide a structural basis for evaluating how PTK7 engagement may alter the local conformation of the Sgc8c arm and potentially weaken its interaction with the complementary lock strand, thereby supporting the proposed target-responsive allosteric activation mechanism. Detailed modeling procedures have been added to the Supplementary Methods.

It should also be noted that computational studies in this field have largely concentrated on the binding event between a single aptamer and its cognate target (Int. J. Biol. Macromol. 2025, 316, 144318; Biochimie 2021, 188, 111-127). For larger, aptamer-driven DNA nanostructures such as the DNO system, experimental validation has been the predominant approach for establishing mechanism and function (Nano Today 2018, 21, 86-99; Adv. Sci. 2024, 11, e2401617; Angew. Chem. Int. Ed. 2023, 62, e202307656). Therefore, after establishing these DNO-level structural models, we further performed two simplified computational analyses to examine the early recognition and local aptamer–lock coupling processes. First, we carried out exploratory free-binding simulations to examine the early encounter between Sgc8c and PTK7 before lock release (Figure S3). In three independent 100 ns unrestrained simulations, Sgc8c was initially placed near the PTK7 Ig3 –Ig4 domain. In two simulations, Sgc8c spontaneously approached a basic-residue-enriched region on the Ig3 surface within the first 20 ns (Figure S2, blue sticks). Although stable complex formation at the reported binding interface was not expected to be fully captured within this limited timescale, this recurrent transient localization suggests that the positively charged region may facilitate the initial electrostatic encounter and subsequent conformational search between Sgc8c and PTK7. Second, to more directly examine whether PTK7 engagement could influence the aptamer–lock interaction, we constructed a simplified sgc8c–lock–PTK7 model (Figure S4). This model retained the key elements required for the proposed allosteric response, including the Sgc8c recognition core, a minimal complementary lock segment, and the PTK7 Ig3–Ig4 domain, while avoiding the excessive size and flexibility of the full DNO assembly. During a 100 ns explicit-solvent simulation, the terminal A33–T9 base pair of the lock duplex showed a time-dependent decrease in hydrogen bonding after PTK7 introduction, from approximately 2 to less than 1 within 40 ns (Figures S4a and S4b). Consistently, MM/GBSA analysis showed a reduction in the calculated lock–sgc8c binding free energy from -24.8 ± 2.1 kcal/mol for the intact 6 bp state to -13.2 ± 1.8 kcal/mol for the partially dissociated 5 bp state, whereas the sgc8c–PTK7 complex remained energetically favorable, with a calculated binding free energy of -31.4 ± 3.2 kcal/mol (Figure S4c). These results suggest that, in this simplified model, PTK7 engagement is associated with local destabilization of the aptamer– lock duplex, providing supportive dynamic evidence for the proposed target-responsive allosteric activation mechanism.

Taken together, these computational analyses provide supportive, multi-level evidence for the proposed target-responsive allosteric activation mechanism. The DNO-level structural models suggest that Sgc8c–PTK7 engagement is geometrically feasible within the assembled architecture. The exploratory free-binding simulations provide additional support for the early Sgc8c–PTK7 encounter before lock release. More directly, the simplified sgc8c–lock–PTK7 model indicates that PTK7 engagement is associated with local destabilization of the aptamer–lock duplex, as reflected by the reduced terminal base-pair hydrogen bonding and decreased calculated lock–sgc8c binding free energy. Together, these results are consistent with the proposed conformational coupling between target binding and lock destabilization. We have added these results as new Supplementary

Figures S2–S4 and included the corresponding statement in the revised manuscript (**Page 5, Lines 118–119**).

We acknowledge that these computational analyses do not constitute definitive proof of the complete allosteric disassembly process, which would require much longer sampling of the full DNO system. Therefore, we have incorporated these results cautiously in the revised manuscript as supportive structural and dynamic evidence. Importantly, previous experimental studies have demonstrated that lock-containing aptamer systems can undergo target-induced conformational switching accompanied by fluorescence signal generation, providing supportive precedent for the mechanistic feasibility of our design (Nat. Commun. 2025, 16, 923; Anal. Chem. 2024, 96, 7747– 7755).

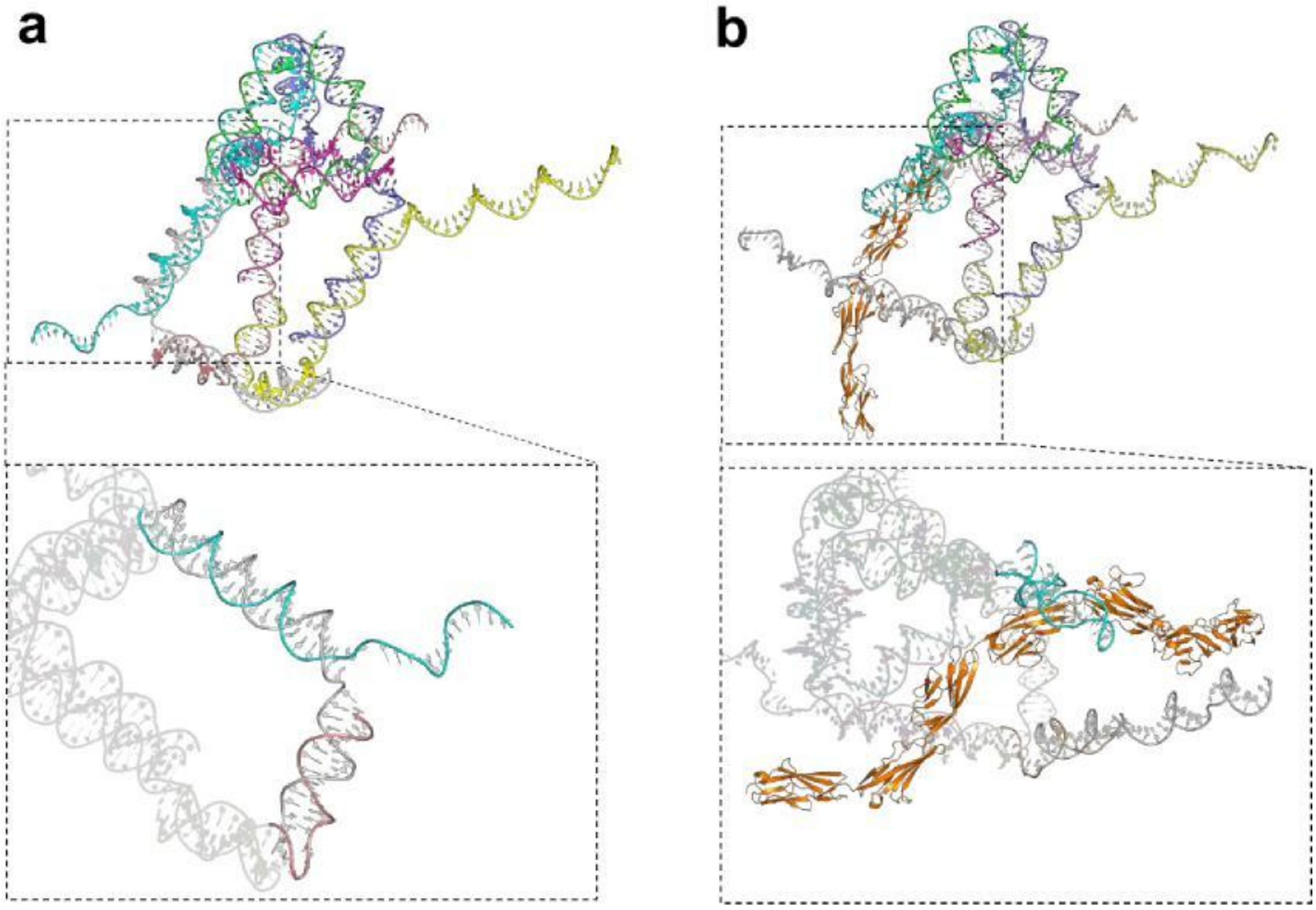

Figure S2. Exploratory structural models of the DNO before and after PTK7 docking. **(a)** Theoretical pre-binding model of the assembled DNO containing the Sgc8c module. **(b)** Theoretical post-docking model of the DNO–PTK7 complex. The orange structure indicates the docked PTK7 Ig3–Ig4 domain. Insets show enlarged local views of the Sgc8c-containing region before and after PTK7 docking.

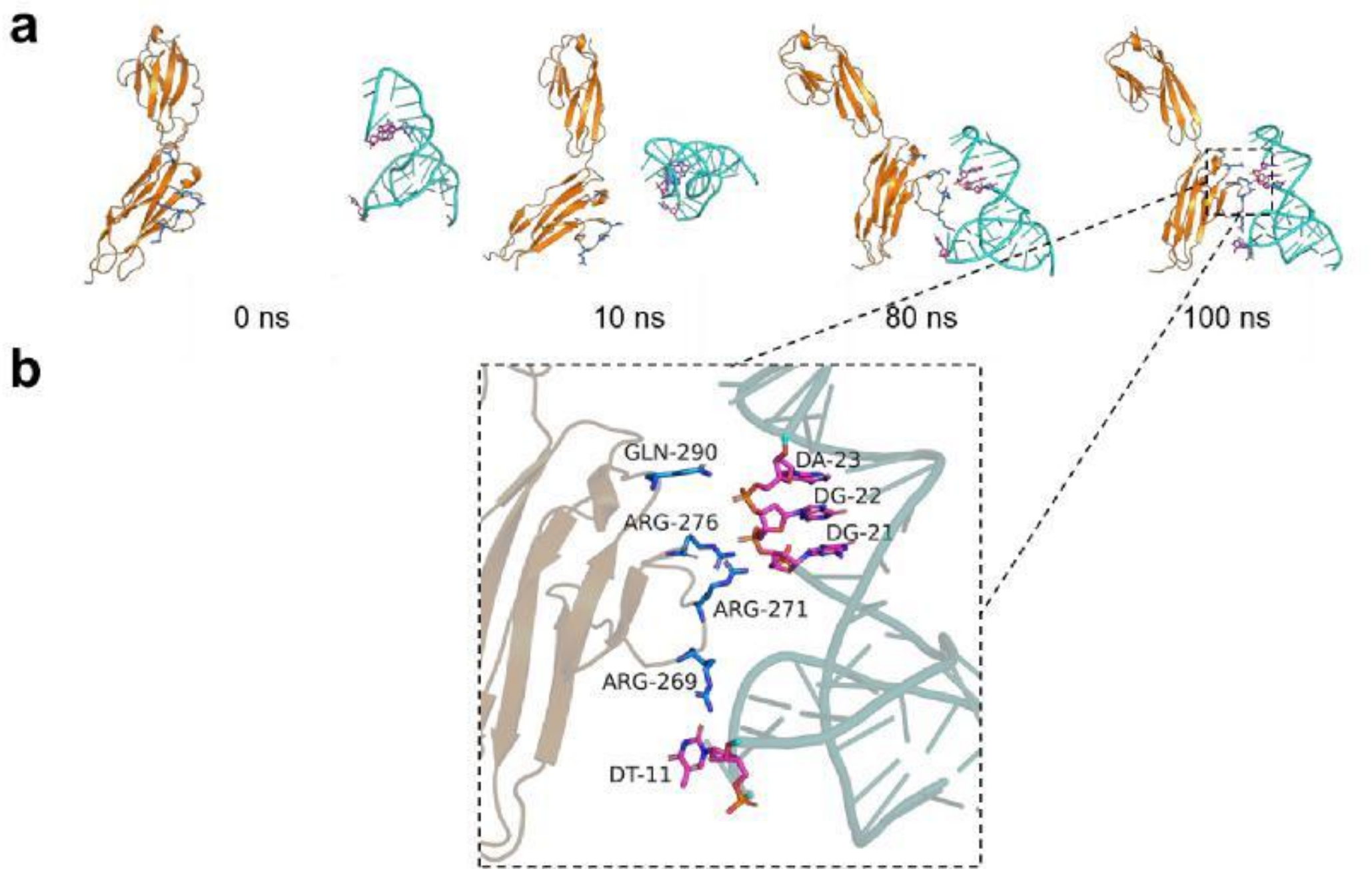

Figure S3. Exploratory free-binding simulation of Sgc8c with PTK7. **(a)** Representative snapshots of Sgc8c and the PTK7 Ig3–Ig4 domain at 0, 10, 80, and 100 ns during an unrestrained simulation. Sgc8c is shown in cyan, and PTK7 is shown in orange. **(b)** Enlarged view of the local contact region between Sgc8c and PTK7, with selected residues and nucleotides labeled.

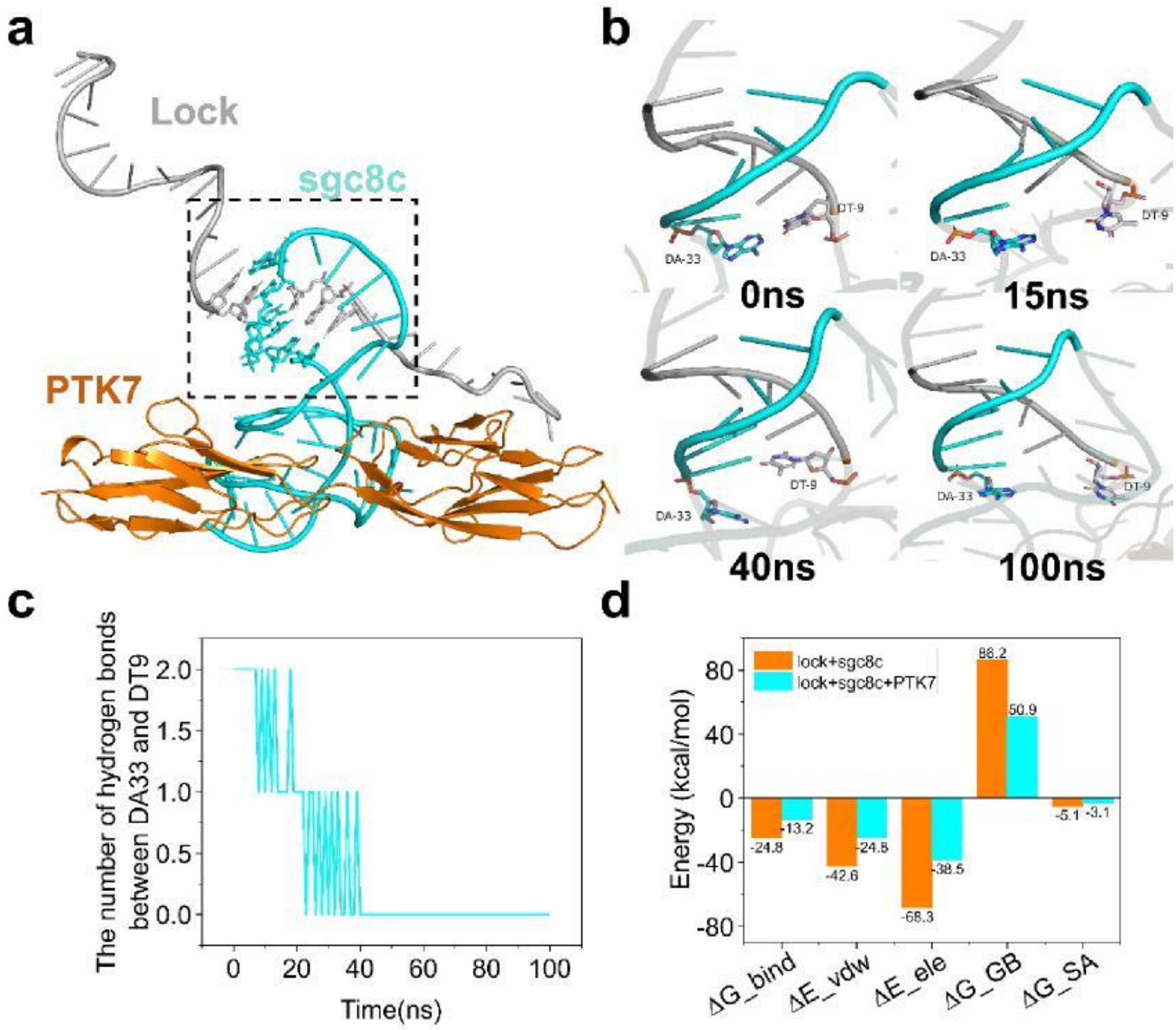

Figure S4. Simplified sgc8c–lock–PTK7 model for evaluating local aptamer–lock coupling. **(a)** Structural model of the sgc8c–lock–PTK7 system. Sgc8c is shown in cyan, the lock strand in gray, and PTK7 in orange. **(b)** Representative enlarged views of the local base-pairing region between sgc8c and the lock strand during the simulation. **(c)** Time-dependent change in the number of hydrogen bonds between DA33 and DT9 of the lock duplex during the 100 ns simulation. **(d)** MM/GBSA energy analysis of the lock–sgc8c interaction in the absence and presence of PTK7.

7. Minor language polishing is recommended.

Response: Thank you for this suggestion. We have carefully polished the language throughout the revised manuscript. To further improve the linguistic quality, the manuscript was also professionally edited by Springer Nature Author Services, and an editing certificate has been provided for reference.

SPRINGER NATURE

Author Services

Editing Certificate

This document certifies that the manuscript

A Membrane-Anchored Biomimetic DNA Nano-Octopus with Allosteric Aptamer Arms Enables Parallel Probing and On-Site Drug Release

prepared by the authors

was edited for proper English language, grammar, punctuation, spelling, and overall style by one or more of the highly qualified English speaking editors at SNAS.

This certificate was issued on **April 10, 2026** and may be verified on the SNAS website using the verification code **9CEA-F5A4-64A7-1059-F804**.

Neither the research content nor the authors' intentions were altered in any way during the editing process. Documents receiving this certification should be English-ready for publication; however, the author has the ability to accept or reject our suggestions and changes. To verify the final SNAS edited version, please visit our verification page at secure.authorservices.springernature.com/certificate/verify. If you have any questions or concerns about this edited document, please contact SNAS at support@as.springernature.com.

SNAS provides a range of editing, translation, and manuscript services for researchers and publishers around the world. For more information about our company, services, and partner discounts, please visit authorservices.springernature.com.

8. Does high concentration of DNO@Dox have cytotoxicity? What is the safe concentration range or IC50 value?

Response: Thank you for this important comment. To further address the cytotoxicity of DNO@Dox at higher doses, we extended the tested concentration range and evaluated cell viability in CEM, Ramos, HeLa, and HELF cells after 48 h of treatment. The results showed a clear concentration-dependent reduction in viability in target CEM cells, whereas the viability of Ramos, HeLa, and HELF cells remained above 70-80% throughout the tested range. Nonlinear regression of the CEM dose-response curve gave an apparent IC50 of 29.1 nM (95% CI, 24.89–33.49 nM) under the tested conditions. By contrast, an IC50 value was not reached for Ramos, HeLa, or HELF cells within the tested concentration range. Therefore, the cytotoxicity of DNO@Dox is strongly cell-dependent. These data indicate that DNO@Dox retains marked cytotoxic activity toward target CEM cells, while showing much weaker effects on non-target cells over the same tested range. We have added these results as new Supplementary Figure S23 and clarified this point in the revised manuscript: "Over an extended concentration range, DNO@Dox showed a concentration-dependent cytotoxic effect in CEM cells, with an apparent IC50 of 29.1 nM, but proved largely insensitive in Ramos, HeLa, and HELF cells under identical conditions (Figure S23)." (**page 22, lines 392–394**).

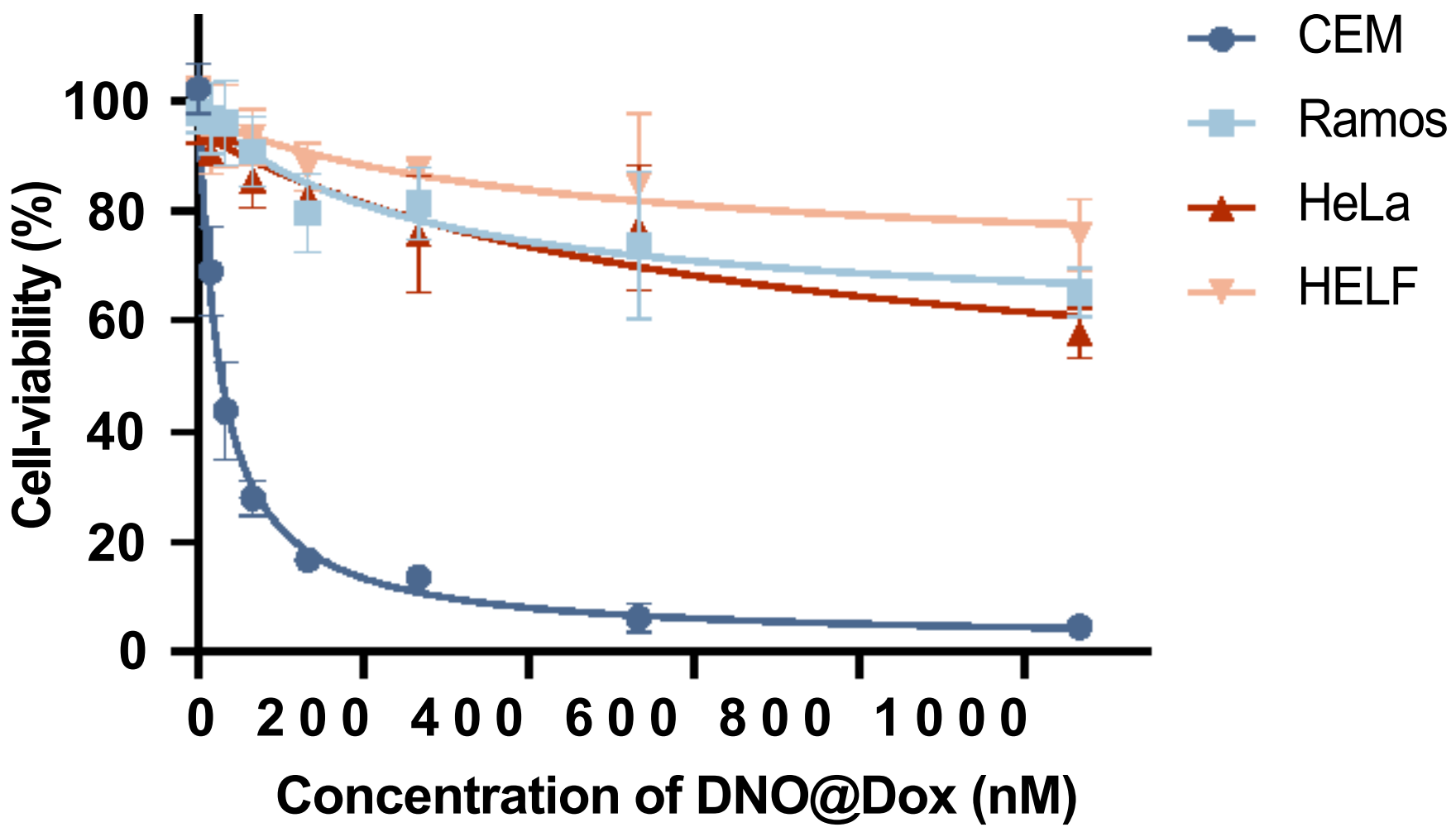

Figure S23. Cell viability over an extended concentration range of DNO@Dox. Cell viability of CEM, Ramos, HeLa, and HELF cells was measured after 48 h of treatment with DNO@Dox at the indicated concentrations. Data are presented as mean ± SD (n = 3).

9. It is highly necessary to clarify the kinetics of DNO@Dox release in vivo and the enrichment rate at the tumor site. Please supplement this part.

Response: Thank you for your great help to improve this paper! To address the release-related kinetics in vivo, we designed an additional fluorescence-reporting construct in which Cy5 was installed on the Apt1 strand and BHQ2 was placed on its corresponding lock strand (new Supplementary Figure S29). In the intact state, Cy5 fluorescence is quenched. Upon target recognition in vivo, disruption of the Apt1-lock interaction leads to fluorescence recovery, thereby providing a real-time readout associated with logic-gated activation and head-release behavior. Whole-body imaging showed that the fluorescence signal in the tumor region became detectable at 0.5 h, increased further at 1 h, reached its highest level at approximately 2 h, and then gradually declined at 4–6 h. This result provides in vivo evidence that the DNO undergoes target-associated fluorescence activation in the tumor region.

To quantify tumor enrichment, we further analyzed the ex vivo fluorescence distribution in major organs and tumors based on the biodistribution data in Figure S28. In this section, the quencher-free system was employed to show the enrichment rate at the tumor site. Based on the ex vivo fluorescence distribution in major organs and tumors, the tumor-associated fluorescence accounted for 7.21% of the total signal in the Lib-DNO group but increased significantly to 24.10% in the DNO group. These results further support that the aptamer-functionalized DNO shows rapid target-triggered fluorescence recovery in vivo and substantially enhanced tumor accumulation compared with the non-targeting control. The corresponding results and discussion have been added to the revised manuscript (**Page 25, Lines 433–439**).

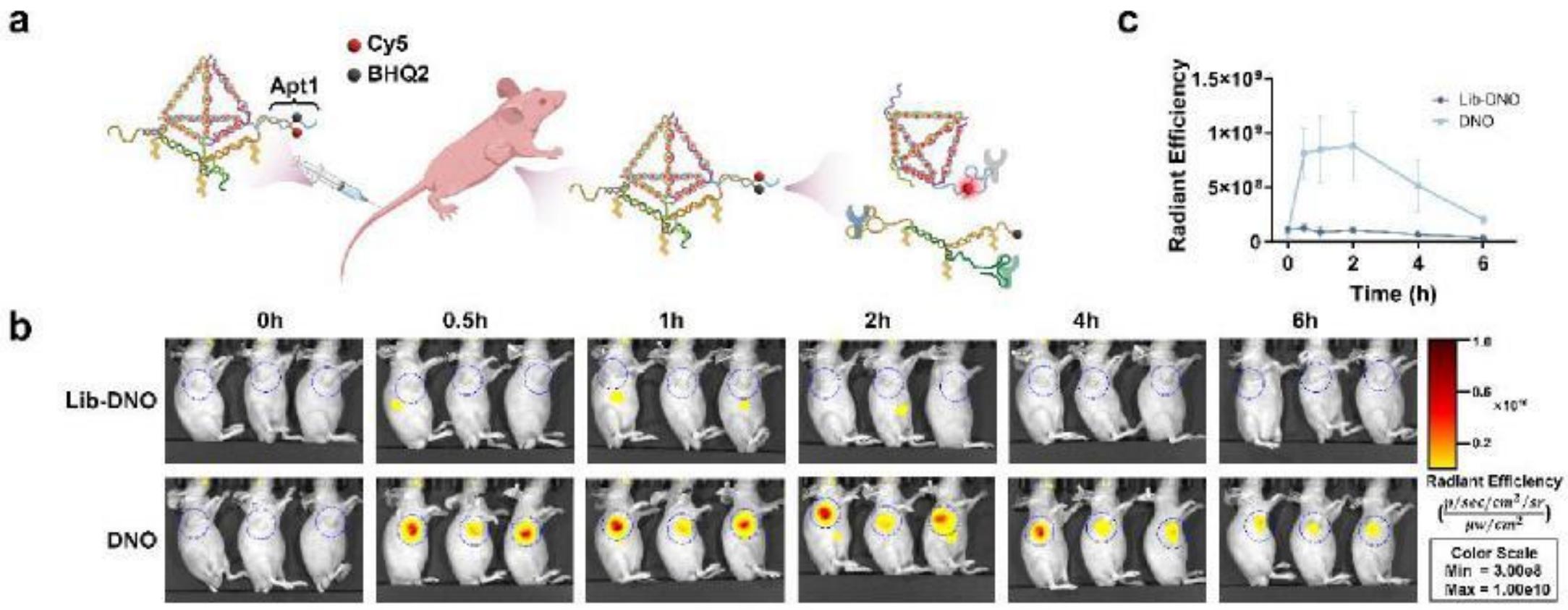

Figure S29. In vivo fluorescence reporting of tumor-associated activation behavior of DNO. **(a)** Schematic illustration of the fluorescence-reporting DNO construct. Cy5 was labeled on the Apt1 strand and BHQ2 was labeled on the corresponding lock strand. In the assembled state, the Cy5 signal was quenched, whereas fluorescence recovery was observed after DNO reached the tumor region and interacted with target cells. **(b)** Representative whole-body fluorescence images of tumor-bearing mice after intravenous injection of Lib-DNO or DNO at the indicated time points. Tumor regions are marked with blue circles. **(c)** Quantitative analysis of fluorescence intensity in the tumor region over time. Data are presented as mean ± SD (n = 3).

10. The difference in the in vivo effects between Lib-DNO@Dox and DNO@Dox is due to the targeting sequences of the aptamers or the release effect?

Response: We thank the reviewer for this insightful comment. In our view, the different in vivo effects between Lib-DNO@Dox and DNO@Dox do not result from a single factor based on the discussion and responses provided in the previous comments. Rather, they reflect the combined contribution of both aptamer-mediated tumor targeting and target-associated release and internalization—two processes that are inherently coupled because they both originate from the same aptamer recognition event. The aptamer sequences serve a dual function: first, they enable specific recognition of cell-surface receptors, thereby directing the nanocarrier to the tumor site (targeting); second, upon receptor engagement, they undergo a conformational switch that triggers strand displacement and DNO disassembly, leading to localized tFNA@Dox release and subsequent internalization (release).

11. Please check if the annotations in Figure 6e (Lib-BNO@Dox, BNO@Dox) are incorrect.

Response: Thank you for pointing this out. We have corrected the labels in Figure 6e to "Lib-DNO@Dox" and "DNO@Dox" in the revised manuscript.

12. A single aptamer is generally sufficient for target recognition. The rationale and mechanistic advantages of using three aptamer arms instead of one are not clearly explained and should be clarified.

Response: We thank the reviewer for this insightful comment. Many tumor-associated surface markers are not unique to target tumor cells. For example, IOSE80 is a normal human ovarian epithelial line that expresses the PTK7 receptor (target of the Sgc8c aptamer), as previously characterized (J. Am. Chem. Soc. 2020, 142, 1265–1277). Therefore, a strategy relying solely on a

single PTK7 aptamer such as sgc8c for recognition or delivery would still carry a risk of off-target recognition and toxicity. To address this limitation, a combinatorial marker profile can be employed by using multiple aptamers that recognize distinct receptors, thereby substantially improving recognition precision over single-receptor detection. However, multi-aptamer recognition alone is still insufficient to fully prevent off-target uptake. As shown in new supplementary Figure S25a, we synthesized a control construct, designated 3apt-tFNA@Dox, in which all three aptamers (Sgc8c, Sgc4f, and TCO1) were directly conjugated to the tetrahedral framework without the Chol-MAS module. Its cellular uptake behavior was then compared with that of the full DNO@Dox system. Cy5 labeling at the tetrahedral vertex was used to monitor the nanocarrier, whereas the intrinsic fluorescence of doxorubicin (Dox) was used to assess intracellular drug delivery. The 3apt-tFNA@Dox group still produced obvious intracellular signals in the PTK7-positive normal cell line IOSE80, indicating that even with multiple aptamers, non-specific internalization may still occur in the absence of additional gating constraints. By contrast, the present DNO design further integrates three-marker combinatorial recognition with membrane anchoring and conditional structural release, thereby imposing a much stricter criterion for target-cell identification and improving selectivity.

To address this concern, we have revised the manuscript accordingly to clarify the rationale and functional advantage of the three-aptamer design. Specifically,

1. In the Working Principle section, we now explicitly state that the role of the three aptamer arms is not merely to enhance binding, but to convert receptor recognition into a combinatorial decision process that enables conditional release of the tetrahedral module: “The three allosteric aptamer arms were introduced not simply to enhance binding, but to convert receptor recognition into a combinatorial decision process, so that release of the tetrahedral module occurs only when the predefined multi-marker condition is satisfied.” (**Page 5, Lines 112–115**).
2. In the Discussion, we further clarified that this design addresses a key limitation of single-receptor targeting, namely its limited ability to exclude nontarget cells with partially overlapping marker expression (**Page 28, Lines 483–485**).
3. In addition, we newly performed and added a comparative uptake study (Figure S25) to support this point experimentally, showing that control constructs still exhibited off-target uptake, whereas the integrated DNO design showed the clearest selectivity (**Page 23, Lines 397–401**).

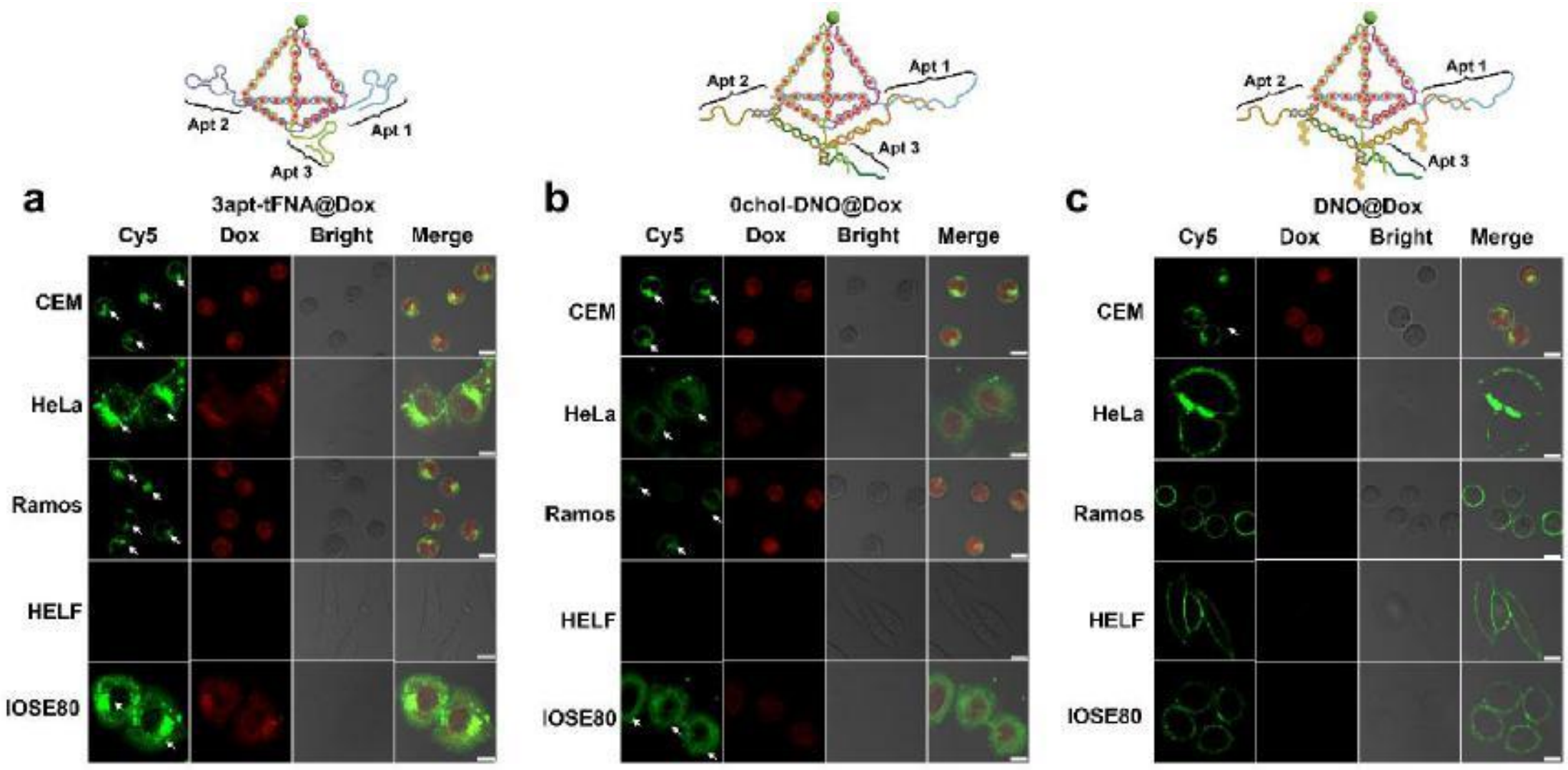

Figure S25. Comparative study of the cellular localization and uptake of DNO@Dox and related control constructs in different cell lines. **(a)** 3apt-tFNA@Dox, **(b)** 0chol-DNO@Dox, and **(c)** DNO@Dox. Confocal images were taken after 2 h incubation at 37 °C. Cy5 tracks the nanostructure, while intrinsic Dox fluorescence monitors drug delivery. Scale bars, 10 μm.

Response to Editor and reviewers

Response to the Editor:

We sincerely thank the editor for the positive editorial decision and helpful guidance. We have revised the manuscript and accompanying files according to the Author Checklist. Since the reviewers had no further substantive comments, brief responses are provided below.

Reviewer #1:

No further comments.

Response: We sincerely thank the reviewer for the positive assessment and for taking the time to evaluate our manuscript.

Reviewer #2:

I recommend acceptance of this paper. It is well written and I have no comments. Response: We sincerely thank the reviewer for recommending acceptance of our manuscript and for the positive comments.
